# A push–pull unsymmetrical subphthalocyanine dimer†
†Electronic supplementary information (ESI) available. See DOI: 10.1039/c5sc01709b


**DOI:** 10.1039/c5sc01709b

**Published:** 2015-06-17

**Authors:** Germán Zango, Johannes Zirzlmeier, Christian G. Claessens, Timothy Clark, M. Victoria Martínez-Díaz, Dirk M. Guldi, Tomás Torres

**Affiliations:** a Departamento de Química Orgánica , Universidad Autónoma de Madrid , Cantoblanco , 28049 Madrid , Spain . Email: tomas.torres@uam.es ; Email: victoria.martinez@uam.es; b Department of Chemistry and Pharmacy , Interdisciplinary Center for Molecular Materials (ICMM) , Friedrich-Alexander-Universitaet Erlangen-Nuernberg , Egerlandstraße 3 , 91058 Erlangen , Germany . Email: dirk.guldi@fau.de; c Computer Chemie Centrum , Department of Chemistry and Pharmacy , Friedrich-Alexander-Universitaet Erlangen-Nuernberg , Naegelsbachstraße 25 , 91052 Erlangen , Germany . Email: tim.clark@fau.de; d IMDEA-Nanociencia , c/Faraday 9, Cantoblanco , 28049 Madrid , Spain.

## Abstract

Unsymmetrical subphthalocyanine fused dimers have been prepared from appropriate *ortho*-dinitrile SubPc precursors. In particular, either electron-donating or electron-accepting substituents have been introduced on each SubPc constituent unit, resulting in unprecedented push–pull π-extended curved aromatic macrocycles. From fluorescence experiments in solvents of different polarity we conclude a dual fluorescence, namely a delocalized singlet excited state (1.73 eV) and a polarized charge transfer state (<1.7 eV). Pump probe experiments corroborate the dual nature of the fluorescence. On one hand, the delocalized singlet excited state gives rise to a several nanosecond lasting intersystem crossing yielding the corresponding triplet excited state. On the other hand, the polarized charge transfer state deactivates within a few picosesonds. Visualization of the charge transfer state was accomplished by means of molecular modeling with a slight polarization of the HOMO towards the electron donor and of the LUMO towards the electron acceptor.

## Abstract 2

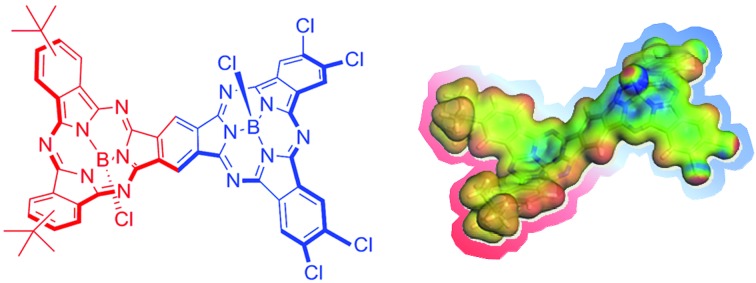
Unsymmetrical subphthalocyanine fused dimers have been prepared, resulting in unprecedented push–pull π-extended curved aromatic macrocycles.

## Introduction

The interesting electronic properties of π-extended materials such as graphene^1^ are the result of their flat two-dimensional π-conjugated systems. Curved π-conjugated systems^2^ are much rarer, despite major advantages such as enhanced solubility, decreased aggregation by π–π stacking, *etc.* Curved polyaromatics, like corannulenes, circulenes, sumanenes and their derivatives, have been prepared and studied intensively.^3^ Introducing heteroatoms into the aromatic π-extended frameworks results in molecular building blocks that exhibit unusual properties, like, for instance, metal coordination provided by the presence of nitrogen atoms. In this respect, planar porphyrins and/or phthalocyanines (Pc)^4^ are among the most relevant examples of macroheterocyclic aromatic compounds.

In contrast, the lower homologues of phthalocyanines, subphthalocyanines (SubPc)^5^ are curved aromatic macrocycles, whose geometry is imposed by the coordination of the three constituent diimino-isoindoles to a central tetrahedral boron. Dimers of subphthalocyanines (SubPc)_2_, that is, π-extended systems comprising two SubPcs fused through a common benzene ring, have been synthesized and fully characterized.^6^ The geometry of the constituent SubPcs endowed the corresponding dimers with an unusual bowl-shape topology, in which two topoisomers, the *syn* and the *anti*, are formed in equal amounts. The *syn* and *anti* SubPc dimers exhibit similar spectroscopic features and a remarkable bathochromic shift of *ca.* 120 nm of their Q-band absorptions to about 700 nm relative to their parent monomers.

SubPcs have been used successfully in evaporated planar heterojunction photovoltaic devices.^7^ Very recently, an outstanding 8.4% efficiency has been reported.^8^ Moreover, highly efficient all SubPc-based photovoltaic devices have been fabricated using an electron accepting (F_8_SubPc)_2_ dimer and a complementary absorbing SubPc monomer as electron donor.^9^

**Chart 1:**
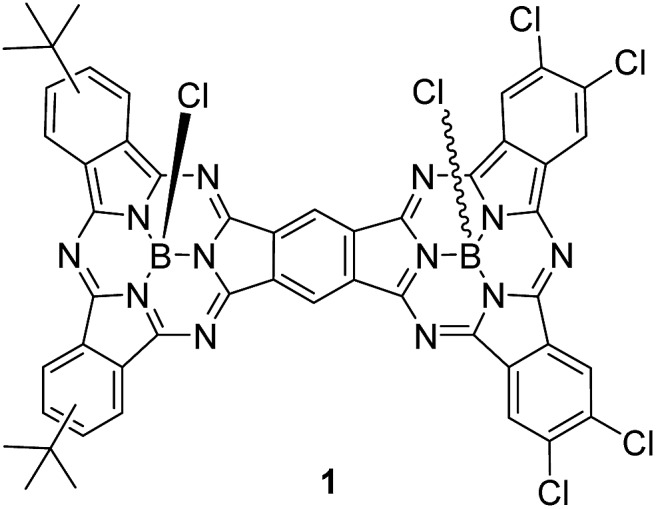
Structure of unsymmetrical SubPc–SubPc′ dimer **1** (mixture of *syn* and *anti* topoisomers).

The preparation of unsymmetrical SubPc dimers has been pursued intensively in our group with the goal of modulating the optoelectronic properties of this family of molecular building blocks further. We now describe a push–pull SubPc–SubPc′ dimer **1** – Chart 1 – that exhibits an unsymmetrical electronic distribution along the curved aromatic surface.

In particular, electron-donating *tert*-butyl and electron-withdrawing chloro groups have been introduced into each SubPc-moiety. The stepwise route described goes through an unsymmetrically substituted *ortho*-dicyano SubPc **2**, which is used as a π-extended *o*-dinitrile precursor. Recently, Kobayashi and Shibata have described a phthalocyanine-subphthalocyanine (Pc-SubPc) heterodinuclear fused dimer starting from an *ortho*-dicyano Pc.^10^ This new π-extended conjugate is a hybrid of the two original constituents, half flat–half concave, and exhibits intermediate spectroscopic properties, but no push–pull character was evident.

## Results and discussion

**Scheme 1:**
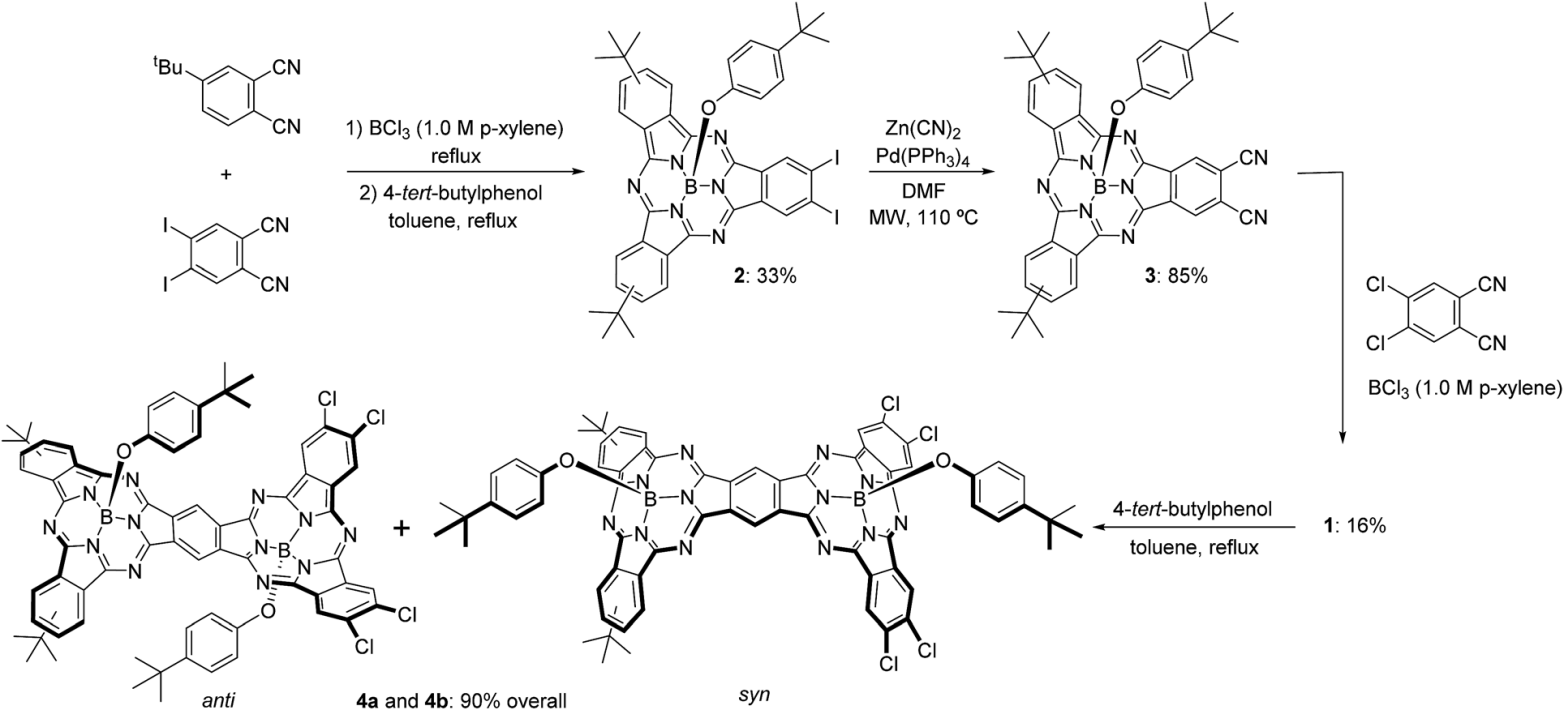
Synthesis of SubPc–SubPc′ dimer **1** and separation of topoisomers **4a** and **4b**.

The synthetic pathway for SubPc–SubPc′ dimer **1** is shown in Scheme 1. DiiodoSubPc **2** was prepared in 33% yield by standard cross condensation of 4-*tert*-butylphthalonitrile and 4,5-diiodophthalonitrile^11^ (2 : 1 molar ratio) in the presence of BCl_3_ (1 eq.) in *p*-xylene, followed by axial chlorine atom substitution with 4-*tert*-butylphenol in excess. **2** consists of an equimolar mixture of three regioisomers with different symmetries (see ESI†), which can be separated by subsequent column chromatography. Despite SubPcs being sensitive to cyanide-anion,^12^ a mild palladium-mediated coupling reaction of SubPc **2** with zinc cyanide (2.4 equivalents) under microwave irradiation (20 W) in DMF at 110 °C during 18 min furnishes the desired *ortho*-dicyano SubPc **3** in 85% yield. Without microwave irradiation, a mixture of starting material **2**, *ortho*-monocyano-monoiodo-SubPc, and SubPc **3** was obtained after 24 h of heating in DMF at 110 °C. Higher reaction temperatures and/or longer reaction times led to an increase in the degree of SubPc decomposition.

Finally, SubPc–SubPc′ dimer **1** was synthesized in 16% yield as a 1 : 1 mixture of *syn*- and *anti*-topoisomers, by reaction of SubPc **3** – either a mixture of regioisomers or a single regioisomer – with 4 equivalents of 4,5-dichlorophthalonitrile in the presence of 6 equivalents of BCl_3_ in *p*-xylene under reflux. Full substitution of the axial ligand in the starting *ortho*-dicyano SubPc **3** by a chlorine atom took place during the reaction.^13^ Separation of *syn*- and *anti*-topoisomers of dimer **1** was not possible by column chromatography on silica gel. However, replacement of the two chlorine atoms in **1** by 4-*tert*-butylphenol allowed for the separation of both topoisomers **4a** and **4b** in 90% overall yield. The structures of all compounds were confirmed by ^1^H-NMR, IR, UV-vis, and HR-MALDI-MS.

The ^1^H-NMR (500 MHz) spectrum of **1** in CDCl_3_ exhibits several diagnostic singlets between 10.40 and 10.35 ppm, corresponding to the two highly deshielded protons of the central benzene ring. The absorption spectra of **1–3** show a B/Soret band at *ca.* 320 nm and a Q band in the visible region. The maximum of the Q band absorption is subject to a bathochromic shift on going from diiodoSubPc **2** (580 nm) to dicyanoSubPc **3** (598 nm). This trend indicates the strong influence of the *ortho*-dinitriles on the π-conjugation of the SubPc aromatic structure. In contrast, the absorption spectrum of SubPc–SubPc′ dimer **1** reveals a Q band maximum at 712 nm, which is 114 nm red-shifted relative to that of SubPc **3**. It is notable that the extinction coefficients also increase as the extent of the π-system increases.

The electrochemical behaviour of **1–4** was studied by cyclic voltammetry in THF – see Table 1 and ESI, Fig. S25 and S26.† DiiodoSubPc **2** exhibits a first reversible reduction at –1481 mV followed by a second one at –2008 mV relative to Fc/Fc^+^. An irreversible oxidative process is observed at 668 mV. Incorporating two nitriles in the periphery of SubPc **3** renders the reduction much easier; SubPc **3** shows a first reversible reduction at –1221 mV, 260 mV positively shifted relative to that of SubPc **2**. A second reversible and a third irreversible reduction are seen at –1744 and –2289 mV, respectively. Compared to SubPc **2**, a more difficult irreversible oxidation is observed for SubPc **3** at 806 mV.

**Table 1:** Electrochemical potentials, optical band gaps and HOMO–LUMO energy levels

	*E* 1 1/2,red ^*a*^ (mV)	*E* 1 ox ^*a*^ ^,^ ^*b*^ (mV)	*E*_LUMO_ ^*c*^ (eV)	*E*_0–0_ ^*d*^ (eV)	*E*_HOMO_ (eV)
**1**	–1088	640	–3.71	1.73	–5.44
**2**	–1481	668	–3.32	2.13	–5.45
**3**	–1221	806	–3.58	2.07	–5.65
**4a**	–1215	589	–3.59	1.72	–5.31
**4b**	–1217	569	–3.58	1.73	–5.31

^*a*^[10^–3^ M] in THF *vs.* Fc/Fc^+^, Pt working electrode, Pt counter electrode, 20 °C, 0.1 TBAPF_6_, scan rate = 100 mV s^–1^.

^*b*^Peak oxidation potential by square wave voltammetry.

^*c*^Calculated with respect to ferrocene, *E*_HOMO_: –4.8 eV.

^*d*^Estimated from the intersection between the absorption and emission spectra.

SubPc–SubPc′ dimer **1** exhibits reduction processes at –1088, –1392, and –1801 mV. Hereby, the first reduction process is shifted anodically by 133 mV relative to SubPc **3**. An irreversible oxidation process is observed at 640 mV, shifted cathodically by 164 mV relative to **3**. As expected, dimers **4a** and **4b** present slight differences in their reduction and oxidation processes compared to **1** due to axial substitution with donor 4-*tert*-butylphenoxy groups, and both topoisomers show exactly the same electrochemical behaviour.

**Chart 2:**
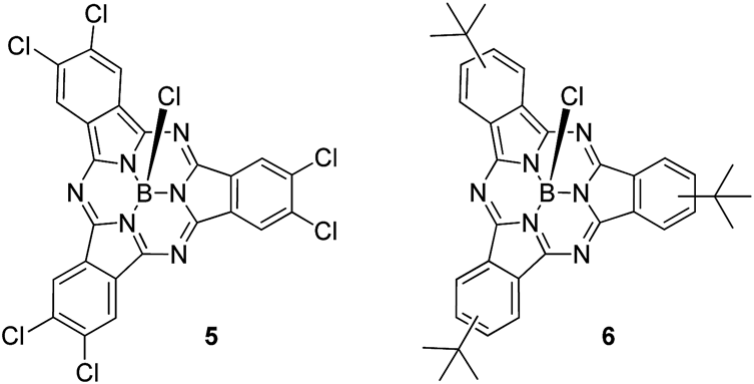
Structures of hexachloro-substituted SubPc **5** and tri-*tert*-butyl-substituted SubPc **6**.

First insights into potential push–pull interactions in the SubPc–SubPc′ dimer **1** came from absorption and fluorescence measurements. In the two references, hexachloro-substituted SubPc **5** and tri-*tert*-butyl-substituted SubPc **6** – Chart 2 – the long wavelength absorptions are seen at 574 and 569 nm in toluene – see ESI.† Importantly, changing the solvent polarity from toluene to benzonitrile exerts only a marginal impact on the vibrational progression of the absorption features, in general, and the long wavelength absorption maxima, in particular. In direct contrast to the absorption, chloro-substituted SubPc **5** (2.15 eV) and *tert*-butyl-substituted SubPc **6** (2.16 eV) exhibit fluorescence with distinct vibrational progression and with Stokes shifts of 211 and 229 cm^–1^. Here, the fluorescence maxima, fluorescence quantum yields, and fluorescence lifetimes in toluene are 581 nm, 28.6 ± 2.5%, and 3.65 ns, respectively, for the electron accepting chloro-substituted SubPc **5**, and 577 nm, 26.5 ± 2.5%, and 3.29 ns for the electron donating *tert*-butyl-substituted SubPc **6**. It is notable that none of the fluorescence features change when the solvent polarity is varied systematically.

**Fig. 1:**
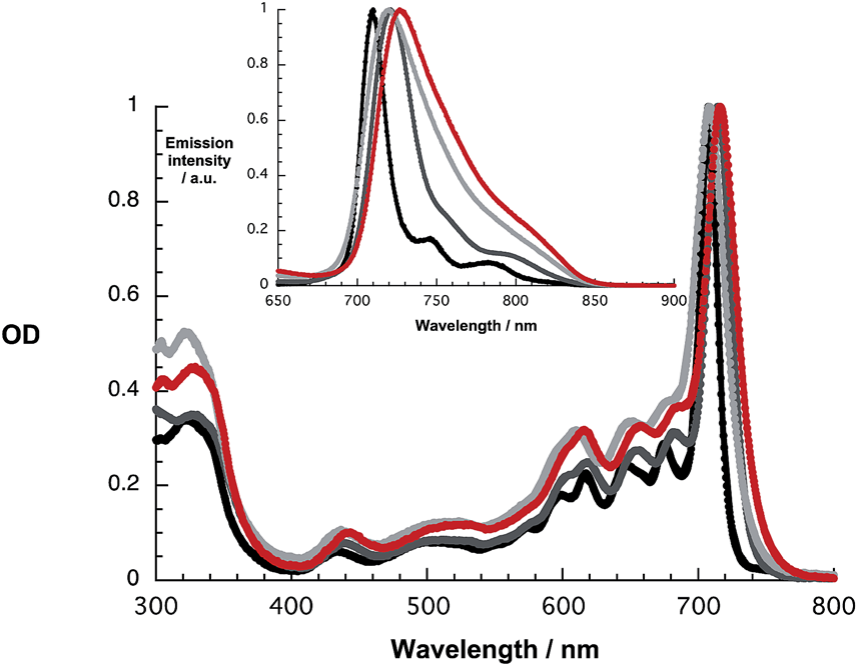
Absorption and fluorescence spectra (inset) upon 610 nm photoexcitation of **1** in cyclohexane (black), toluene (dark grey), THF (light grey), and benzonitrile (red).

At first glance, the absorption and fluorescence of SubPc–SubPc′ dimer **1** – Fig. 1 – are similar to those seen for the references described above despite a substantial red shift. For example, in toluene the vibrationally fine structured absorption and fluorescence include a long wavelength maximum at 715 nm and a short wavelength maximum at 721 nm, respectively. In THF and benzonitrile, the corresponding features peak at 708/720 and at 716/727 nm, respectively. In contrast to the invariance of the maxima, the fluorescence quantum yields drop as the solvent polarity increases from cyclohexane (19.2%) and toluene (16.1%) to THF (8.3%) and benzonitrile (7.3%). A closer look reveals, however, that the fluorescence features an additional, rather broad component, whose maximum is subject to some red shifts as the solvent polarity is increased, but determination of the exact location turned out to be difficult. We postulate that a delocalized singlet excited state (1.73 eV) is the origin of the former, while the latter is due to a polarized charge transfer state (<1.7 eV).

This hypothesis was confirmed in pump-probe experiments, in which chloro-substituted SubPc **5**, *tert*-butyl-substituted SubPc **6**, and SubPc–SubPc′ dimer **1** were photoexcited at 530 and 656 nm. For **5** and **6**, we find differential absorption spectra immediately upon photoexcitation – see ESI.† These include the features of the singlet excited states. For example, for chloro-substituted SubPc **5** in toluene, transient bleaching from 510 to 608 nm with a minimum at 578 nm reflects the ground-state absorption. Maxima at 428 and 648 nm, a shoulder at 620 nm, and a broad NIR band complete the singlet excited state features of chloro-substituted SubPc **5**. The differential absorption spectrum of *tert*-butyl-substituted SubPc **6** reveals a minimum at 574 and maxima at 446 and 620 nm, in addition to a shoulder at 645 nm. From multi-wavelength analyses, we estimate solvent-independent singlet excited state lifetimes of 2.9 ± 0.1 ns for chloro-substituted SubPc **5** and 3.0 ± 0.15 ns for *tert*-butyl-substituted SubPc **6**. These decay lifetimes imply an efficient intersystem crossing to the corresponding triplet excited states. For the triplet excited state, the most prominent features in toluene are a 574 nm minimum with a 553 nm shoulder and 444, 617 and 672 nm maxima for chloro-substituted SubPc **5** and a 571 nm minimum with a shoulder at 550 nm and 458, 610 and 670 nm maxima for the *tert*-butyl-substituted SubPc **6**. They decay with lifetimes on the order of 7.7 ± 0.2 μs and 1.2 ± 0.1 μs and give place to the singlet ground state.

**Fig. 2:**
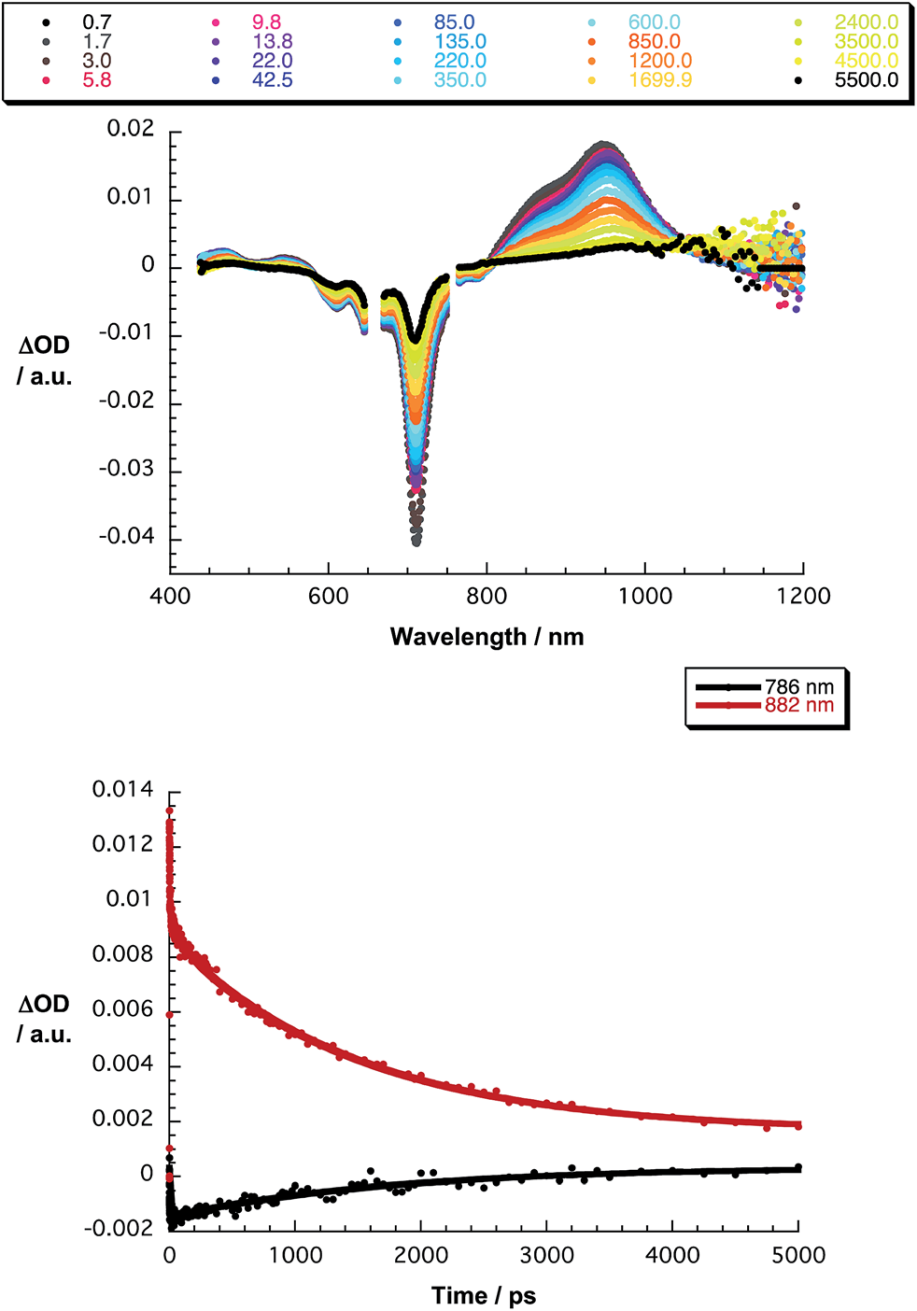
Upper part – differential absorption spectra (visible and near-infrared) obtained upon femtosecond pump probe experiments (656 nm) of SubPc–SubPc′ dimer **1** in THF with several time delays between 0.7 and 5500 ps at room temperature – see figure legend for details. Lower part – time absorption profiles of the spectra at 786 (black spectrum) and 882 nm (red spectrum) monitoring the excited state dynamics.

**Fig. 3:**
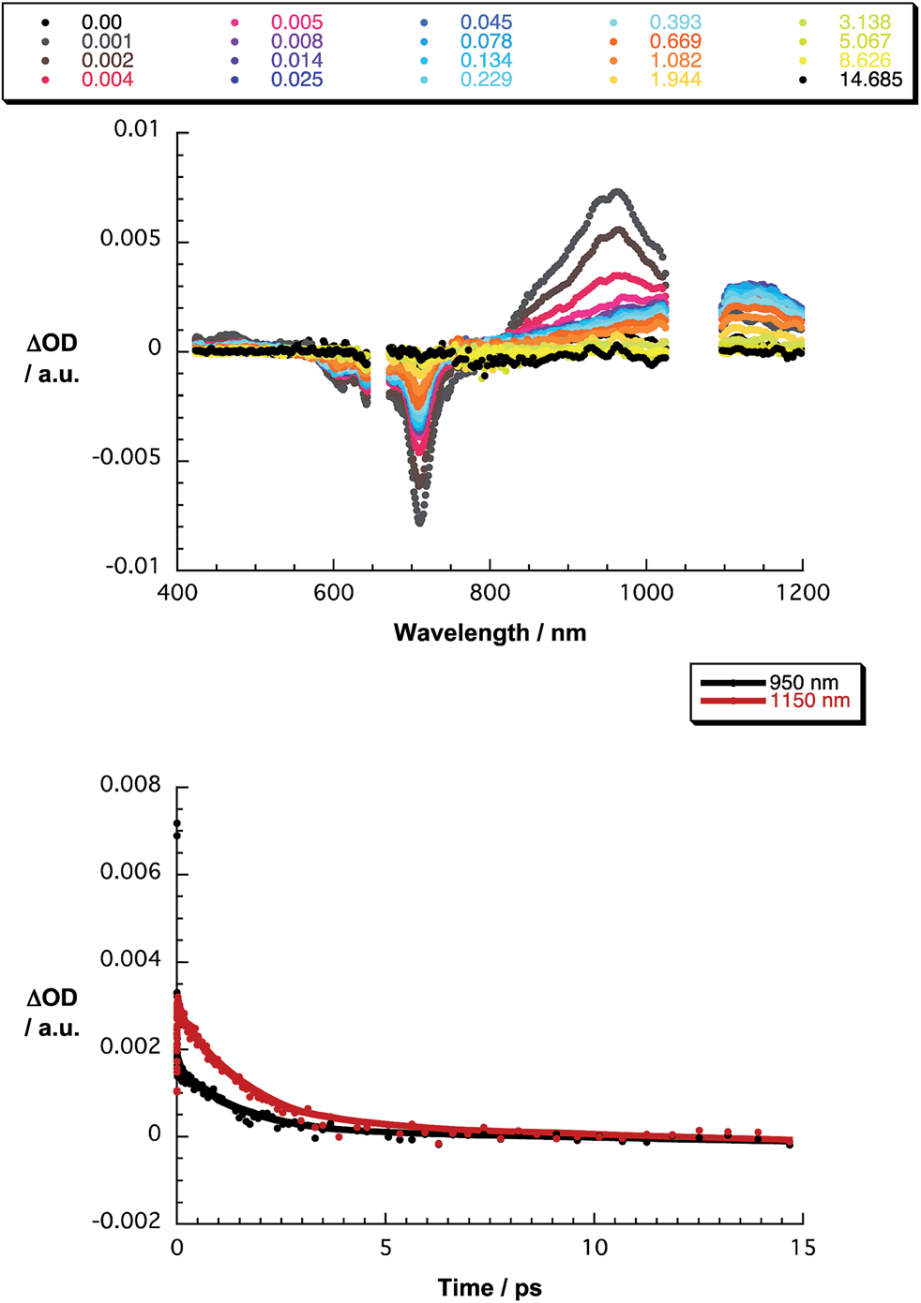
Upper part – differential absorption spectra (visible and near-infrared) obtained upon nanosecond pump probe experiments (656 nm) of SubPc–SubPc′ dimer **1** in THF with several time delays between 0 and 14.685 μs at room temperature – see figure legend for details. Lower part – time absorption profiles of the spectra at 950 (black spectrum) and 1150 nm (red spectrum) monitoring the excited state dynamics.

The final set of experiments is concerned with the SubPc–SubPc′ dimer **1** in pump-probe assays – Fig. 2 and 3. In line with the absorption spectra, the ground-state bleaching in the singlet excited state (1.73 eV) is red shifted relative to those described above for chloro-substituted SubPc **5** (2.15 eV) and *tert*-butyl-substituted SubPc **6** (2.16 eV). In particular, minima develop immediately upon photoexcitation at 656 nm in toluene at 618, 684, and 716 nm, in THF at 611 and 710 nm, and in benzonitrile at 616 and 717 nm. Additionally, maxima are found at 468, 544, and 952 nm in toluene, 470, 541, and 948 nm in THF, and in benzonitrile at 470, 547, and 955 nm. The time evolution of these differential absorption spectra allows us to derive solvent-dependent intersystem crossing lifetimes for the delocalized singlet excited state with values that range from 2.28 ± 0.01 ns in cyclohexane to 1.69 ± 0.03 ns in benzonitrile. These values are in close agreement with those determined in fluorescence lifetime measurements; 2.4 ns in cyclohexane and 1.7 ns in benzonitrile. Evidence for the shorter-lived polarized charge transfer state came in the form of ground-state bleaching at 710 nm and a 870 nm shoulder, but only in the polar solvents THF and benzonitrile. The lifetimes are 3.2 ± 0.3 ps in THF and 5.3 ± 0.5 ps in benzonitrile.

We turned to modeling to rationalize the experimental observations. Geometries were initially optimized for the *syn*- and *anti*-isomers of **1** and for a model phthalocyanine dimer **7** – the structure is shown in the ESI† – using density-functional theory (DFT) with the B3LYP hybrid functional,^14^,^15^ and the 6-31G(d) basis set.^16^–^18^ All DFT calculations used the Gaussian 09 program.^19^ The optimized geometries were confirmed to be local minima by calculating their normal vibrations within the harmonic approximation. Electronic spectra were calculated for these geometries in the gas phase using the AM1 Hamiltonian^20^,^21^ and a configuration-interaction with only single excitations (CIS). CIS calculations used 100 occupied and 100 virtual orbitals in the active space. All semiempirical calculations were performed with our development version of VAMP.^22^ The optimized structures of these three compounds are given in the ESI.† *Syn*- and *anti*-**1** are strongly non-planar, as expected, whereas **7** optimizes to a planar structure. **7** was included in the study in order to quantify the effects of non-planarity on the subphthalocyanine dimers by comparison with a planar model compound.

The calculated spectra of *syn*- and *anti*-**1** are very similar, so that only that for the *anti*-isomer will be discussed in the following. The data for the *syn*-isomer are given in the ESI.† A simulation of the calculated UV/vis spectrum for *anti*-**1** is shown in Fig. S38 of the ESI.† The calculated spectrum shows respectable agreement with the experiment, giving us confidence that AM1/CIS is able to reproduce the major features of the excited states of **1** and **7**.

**Fig. 4:**
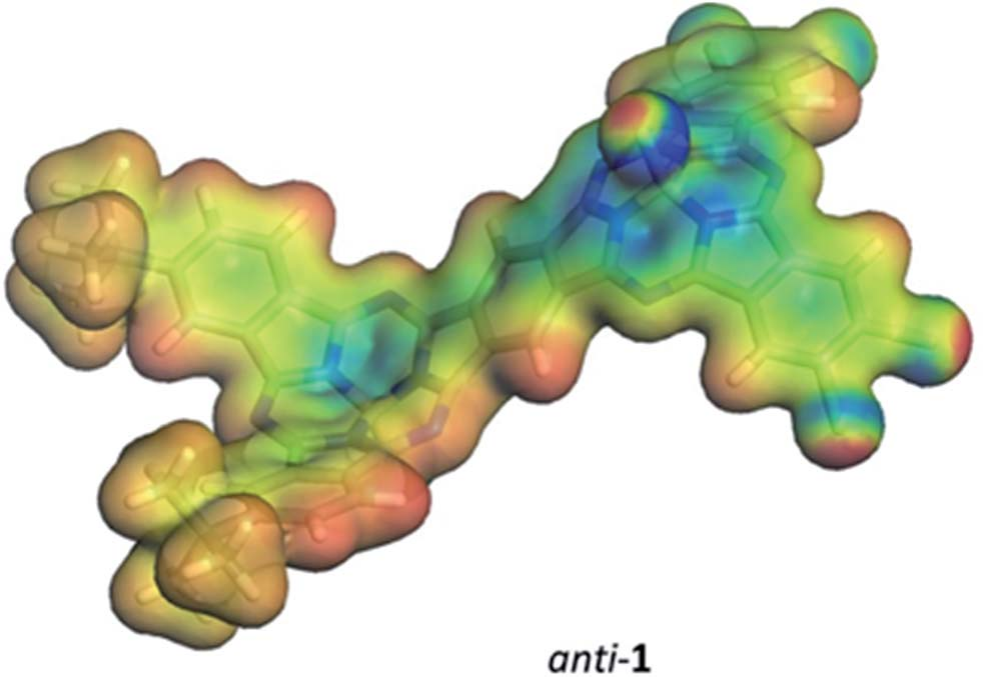
The molecular electrostatic potential calculated at the 0.001 a.u. isodensity surfaces of the S^5^ states of *anti*-**1**. The color scale ranges from –0.3 a.u. (blue) to 0.2 a.u (red) (approximately –190 to +125 kcal mol^–1^).

A detailed analysis of the states involved in the excitations reveals that the S^1^ to S^4^ states have dipole moments that differ by less than 1.5 Debye from the ground state for both *syn*- and *anti*-**1**. A charge-separated state is found in both isomers for S^5^, which occurs close to 410 nm in both cases and gives an excited state with a dipole moment of 19.4 or 18.2 Debyes for *syn* and *anti*, respectively. The planar phthalocyanine dimer **7** shows similar behavior. S^1^ to S^4^ are π–π local excitations and S^5^ (475 nm) is a charge-separated state with a dipole moment of 36.5 Debyes. As the charge-separated states are embedded energetically in a plethora of locally excited ones, strong solvent shifts of the charge-separated state will not necessarily be evident in the experimental spectra. Fig. 4 shows the calculated molecular electrostatic potentials on the 0.001 a.u. isodensity surface of the charge-separated (S^5^) states of *anti*-**1**. It is evident that charge separation is far more pronounced in the planar phthalocyanine dimer **7**. The oscillator strength calculated for the S^0^ → S^5^ transition in **7** (0.27) is also slightly lower than those found for *syn*- (0.31) and *anti*-**1** (0.36), which is consistent with lower charge-separation in the non-planar species **1**.

**Fig. 5:**
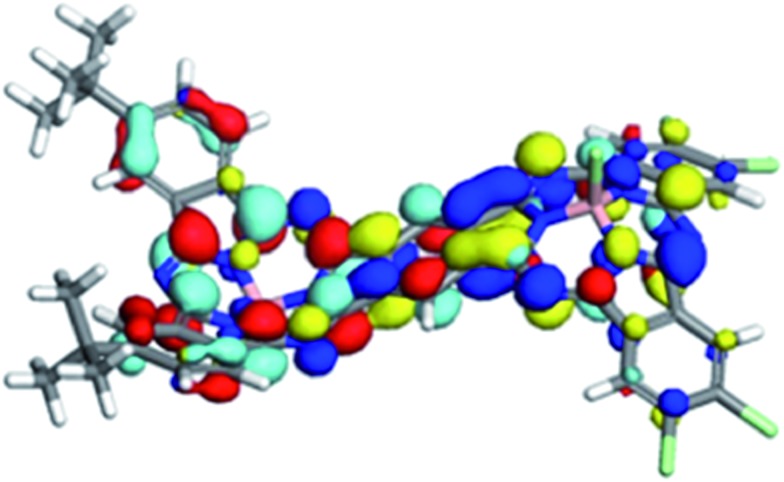
HOMO (red/cyan) and LUMO (blue/yellow) of *anti*-**1**.

The ground states of both isomers of **1** show a small amount of charge separation between the two unsymmetrical halves of the dimer. Fig. 5 shows the calculated (DFT) HOMO and LUMO of *anti*-**1**.

The slight polarization of the HOMO towards the donor and of the LUMO towards the acceptor demonstrate nicely the effect of the donor and acceptor substituents. Self-consistent reaction field (SCRF) AM1-CIS calculations^23^ in water suggest that the locally excite S^1^ and a polar charge-shifted state close in energy are the first two excited states in solution.

## Conclusions

Unsymmetrically substituted SubPc fused dimers have been prepared for the first time by a stepwise methodology, which implies the preparation of *ortho*-dicyano SubPcs, used as π-extended phthalonitrile precursor. The successful key transformation of *o*-diiodo SubPcs into *o*-dicyano derivatives by a palladium-mediated reaction required two equivalents of zinc cyanide and the use of microwave irradiation. Subsequent cross-condensation between *ortho*-dicyano SubPcs and a differently substituted phthalonitrile allowed the preparation of unsymmetrical SubPc dimers as equimolar mixture of *syn* and *anti* isomers, which exhibit similar spectroscopic features. Separation of *syn* and *anti* isomers was achieved by further axial functionalization.

Physico-chemical studies allowed us to probe the push–pull character of SubPc–SubPc′ dimer **1**, bearing *tert*-butyl and chlorine substituents on each SubPc half, and have provided evidence for a charge-polarized curved π-extended system. For example, from fluorescence experiments in solvents of different polarity we conclude a dual fluorescence, namely a delocalized singlet excited state (1.73 eV) and a polarized charge transfer state (<1.7 eV). Further corroboration for the dual nature of the fluorescence came from pump-probe experiments. The delocalized singlet excited state gives rise to intersystem crossing that lasts several nanoseconds to yield the corresponding triplet excited state, while the polarized charge-transfer state deactivates within a few picosesonds. The nature of the charge-transfer state was visualized using the results of DFT calculations. They show a slight polarization of the HOMO towards the electron donor and of the LUMO towards the electron acceptor. Semiempirical CIS calculations confirm the existence of charge-transfer states above low-lying locally excited singlets in vacuum. These charge-transfer states are stabilized relative to the non-polar singlets in SCRF calculations.

## Supplementary Material

Supplementary information
