# Supplementary material for: A push–pull unsymmetrical subphthalocyanine dimer
Source: Chem Sci. 2015 Jun 17;6(10):5571–7. doi: 10.1039/c5sc01709b (PMC5949859; doi:10.1039/c5sc01709b)
Supplement: Supplementary file 1 [file SC-006-C5SC01709B-s001.pdf]

Electronic Supplementary Material (ESI) for Chemical Science.
This journal is © The Royal Society of Chemistry 2015

Supporting Information

A push-pull unsymmetrical subphthalocyanine dimer

Germán Zango,[a] Johannes Zirzlmeier,[b] Christian G. Claessens,[a] Timothy Clark,*[c] M. Victoria Martínez-Díaz,*[a] Dirk M. Guldi*[b] and Tomás Torres*[a,d]

[a] *Departamento de Química Orgánica, Universidad Autónoma de Madrid, Cantoblanco, 28049 Madrid, Spain. E-mail: tomas.torres@uam.es, victoria.martinez@uam.es*
[b] *Department of Chemistry and Pharmacy, Interdisciplinary Center for Molecular Materials (ICMM), Friedrich-Alexander-Universitaet Erlangen-Nuernberg, Egerlandstraße 3, 91058 Erlangen, Germany. E-mail: dirk.guldi@fau.de*
[c] *Computer Chemie Centrum, Department of Chemistry and Pharmacy, Friedrich-Alexander-Universitaet Erlangen-Nuernberg, Naegelsbachstraße 25, 91052 Erlangen, Germany. E-mail: tim.clark@fau.de*
[d] *IMDEA-Nanociencia, c/ Faraday, 9, Cantoblanco, 28049 Madrid, Spain.*

TABLE OF CONTENTS:

1. **General Methods** **2**
2. **Synthetic scheme of compounds 1-3 with determining step for each regioisomer separation** **3**
3. **Synthetic procedures and molecular characterization of compounds 1-4** **4**
   - Compound **2** 4
   - Compound **3** 11
   - SubPc dimer **1** 18
   - SubPc dimers **4a** and **4b** 24
4. **UV-vis absorption spectra, fluorescence emission spectra and electrochemical characterization of compounds 1-4** **27**
5. **UV-vis absorption spectra, fluorescence emission spectra and transient absorption spectra of compounds 1, 5 and 6** **31**
6. **DFT and Semiempirical Configuration-Interaction Calculations** **39**
7. **References** **44**

1. General methods.

All chemicals were purchased from Sigma-Aldrich Co., TCI Europe N.V. and Alfa Aesar and used without further purification. Solvents were purchased from Carlo Erba Reagents and Scharlab. Dry dimethylformamide was prepared using molecular sieves (irradiated with microwaves and dried under vacuum). 4,5-diiodophthalonitrile[1] and 4,5-dichlorophthalonitrile[2] were prepared using a described procedure. Column chromatography was carried out on silica gel Merck-60 and TLC was carried out on aluminium sheets percolated with silica gel 60 F254 (Merck). ^{1}H NMR and ^{13}C NMR spectra were obtained using a Bruker Avance 300 spectrometer or a Bruker DRX-500 spectrometer. Infrared spectra (IR) were recorded on a Bruker Vector 22. Matrix-assisted laser desorption/ionization time of flight (MALDI-TOF) MS and high-resolution mass spectrometry (HRMS) spectra were recorded with a Bruker Reflex III spectrometer. Microwave reactions were carried out in a Biotage Initiator+ system. For the photophysical characterization the samples were placed in fluorometric cuvettes with different pathways and, when necessary purged with argon. Steady-state UV-vis absorption spectra were recorded with a Jasco V-660 instrument and with a Lambda2 spectrometer (Perkin Elmer). Steady state fluorescence spectra were carried out at a JASCO-V8600 and at a FluoroMax3 spectrometer (Horiba). Femtosecond and nanosecond transient absorption (TA) experiments were carried out with an amplified Ti:Sapphire CPA-2110 fs laser system (Clark MXR: output 775 nm, 1 kHz, 150 fs pulse width) using transient absorption pump/probe detection systems (Helios and Eos, Ultrafast Systems). The 530 and 656 nm excitation wavelengths were generated with a noncolinear optical parametric amplifier (NOPA, Clark MXR). Fluorescence lifetimes were determined by the time correlated single photon counting technique using a FluoroLog3 emission spectrometer (Horiba JobinYvon) equipped with an R3809U-58 MCP (Hamamatsu) and a 405LH laser diode (Horiba JobinYvon) exciting at 403 nm (675 ps fwhm) as well as a 650L laser diode (Horiba JobinYvon) exciting at 647 nm (<200 ps fwhm). Electrochemical measurements were performed on an Autolab PGStat 30 equipment using a three electrode configuration system. The measurements were carried out using THF solutions containing 0.1 M tetrabutylammonium hexafluorophosphate ($TBAPF_6$). A glassy carbon electrode (3 mm diameter) was used as the working electrode, and a platinum wire and an $Ag/AgNO_3$ (in CH_3CN) electrode were employed as the counter and the reference electrodes, respectively. Ferrocene (Fc) was added as an internal reference and all the potentials were given relative to the Fc/Fc^+ couple. Scan rate was 100 mV/s.

2. Synthetic scheme of compounds 1-3, with determining step for each regioisomer separation.

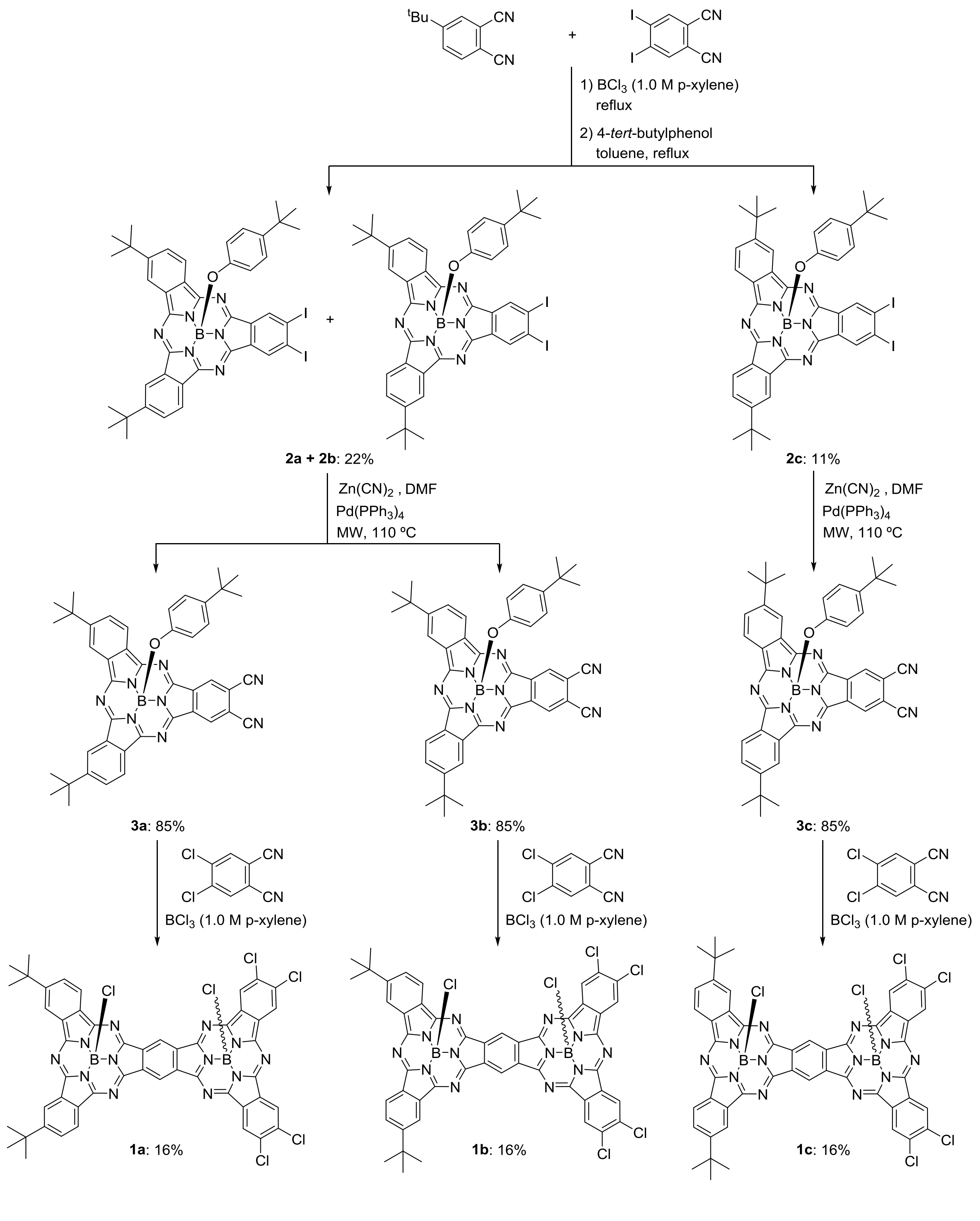

3. **Synthetic procedures and molecular characterization of compounds 1-4.**

Compound 2. To a 25 ml round-bottom two-neck flask equipped with a reflux condenser and magnetic stirrer, a 1.0 M solution of BCl_3 in *p*-xylene (3 mL, 3 mmol) was added to a mixture of 4,5-diiodophthalonitrile (380 mg, 1 mmol) and of 4-*tert*-butylphthalonitrile (368 mg, 2 mmol), under argon atmosphere. The mixture was stirred and heated to reflux (136-138 °C) for 2 h. The crude was cooled down to room temperature and flushed with argon. After evaporation of *p*-xylene, 4-*tert*-butylphenol (750 mg, 5 mmol) was added. The mixture was dissolved in *ca.* 3 mL of toluene and refluxed for 4 hours. After cooling down to room temperature, the excess of phenol was removed by washing the crude with a 3:1 methanol/water solution. The crude product was purified by column chromatography on silica gel using toluene:hexane 3:1 as eluent, yielding product **2** as a purple-red solid (298 mg, 33%).

Regioisomer **2a**:

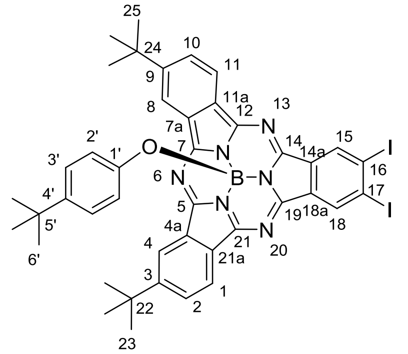

^{1}H-NMR ($CDCl_3$, 300 MHz) δ (ppm): 9.36 (s, 2H, H-15, H-18), 8.89 (d, J_m = 1.6 Hz, 2H, H-4, H-8), 8.73 (d, J_o = 8.4 Hz, 2H, H-1, H-11), 8.00 (dd, J_o = 8.4 Hz, J_m = 1.6 Hz, 2H, H-2, H-10), 6.75 (d, J_o = 8.8 Hz, 2H; H-3’), 5.29 (d, J_o = 8.8 Hz, 2H; H-2’), 1.55 (s, 18H; H-23, H-25), 1.08 (s, 9H; H-6´).
^{13}C-NMR ($CDCl_3$, 75.5 MHz) δ (ppm): 154.5 (C-3, C-9), 153.3 (C-14, C-19), 151.8 (C-5, C-7), 150.1 (C-1’), 148.3 (C-12, C-21), 143.8 (C4’), 132.5 (C-15, C-18), 131.4 (C-4a, C-7a), 130.8 (C-11a, C-21a), 129.2 (C-14a, C-18a), 128.6 (C-2, C-10), 125.8 (C3’), 122.0 (C-1, C-11), 118.9 (C-4, C-8), 117.9 (C-2’), 108.4 (C-16, C-17), 36.0 (C-22, C-24), 34.0 (C-5’), 31.8 (C-23, C-25), 31.5 (C-6’).
UV-Vis ($CHCl_3$): λmax (nm) (log *ε* ($dm^3mol^{-1}cm^{-1}$)) = 580 (4.7), 569 (sh), 541 (sh), 522 (sh), 320 (4.5), 271 (4.6).
FT-IR (film) ν (cm^{-1}): 2962, 2866, 1607, 1510, 1454, 1402, 1385, 1259, 1178, 1063 (B-O), 895, 831, 760, 708.
MS (MALDI-TOF, DCTB), m/z: 908.2 $[M]^+$ (100%), 759.1 $[M – axial group]^+$ (25%). HRMS (MALDI-TOF, DCTB): calc. for: $[M]^+$: m/z: 908.1369 found 908.1360.

Regioisomer **2c**:

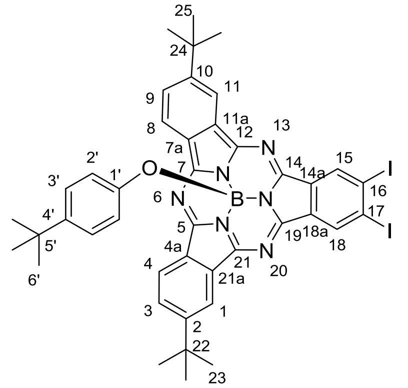

^{1}H-NMR ($CDCl_3$, 300 MHz) δ (ppm): 9.38 (s, 2H, H-15, H-18), 8.84 (d, J_m = 1.6 Hz, 2H, H-1, H-11), 8.75 (d, J_o = 8.4 Hz, 2H, H-4, H-8), 7.99 (dd, J_o = 8.4 Hz, J_m = 1.6 Hz, 2H, H-3, H-9), 6.75 (d, J_o = 8.8 Hz, 2H; H-3'), 5.30 (d, J_o = 8.8 Hz, 2H; H-2'), 1.55 (s, 18H; H-23, H-25), 1.08 (s, 9H; H-6´).
^{13}C-NMR ($CDCl_3$, 75.5 MHz) δ (ppm): 154.8 (C-2, C-10), 153.3 (C-14, C-19), 152.2 (C-5, C-7), 150.1 (C-1'), 148.0 (C-12, C-21), 143.8 (C4'), 132.5 (C-15, C-18), 131.8 (C-4a, C-7a), 130.7 (C-11a, C-21a), 128.9 (C-14a, C-18a), 128.4 (C-3, C-9), 125.8 (C3'), 122.1 (C-4, C-8), 118.7 (C-1, C-11), 117.9 (C-2'), 108.3 (C-16, C-17), 36.0 (C-22, C-24), 34.0 (C-5'), 31.8 (C-23, C-25), 31.5 (C-6').
UV-Vis ($CHCl_3$): λmax (nm) (log ε ($dm^3mol^{-1}cm^{-1}$)): 580 (4.7), 569 (sh), 541 (sh), 522 (sh), 320 (4.5), 271 (4.6).
FT-IR (film) ν (cm^{-1}): 2962, 2866, 1607, 1510, 1454, 1402, 1385, 1259, 1178, 1063 (B-O), 895, 831, 760, 708.
MS (MALDI-TOF, DCTB), m/z: 908.2 $[M]^+$ (100%), 759.1 $[M – axial group]^+$ (90%). HRMS (MALDI-TOF, DCTB): calc. for: $[M]^+$: m/z: 908.1369 found 908.1360.

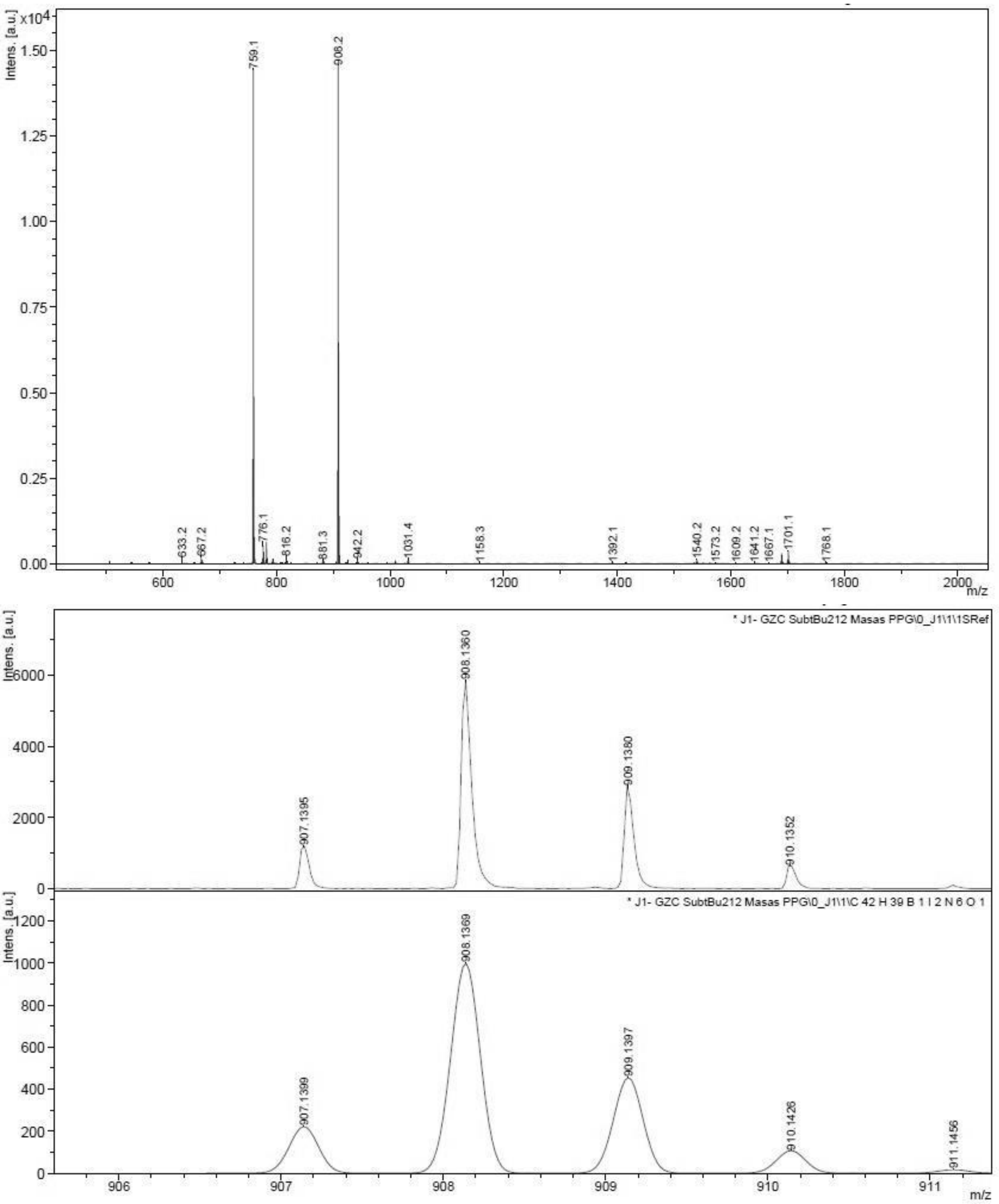

Figure S1. Upper part: MALDI-TOF mass spectrum for **2**. Lower part: A) experimental isotopic pattern and B) calculated isotopic pattern of $[M]^+$ peak of **2**.

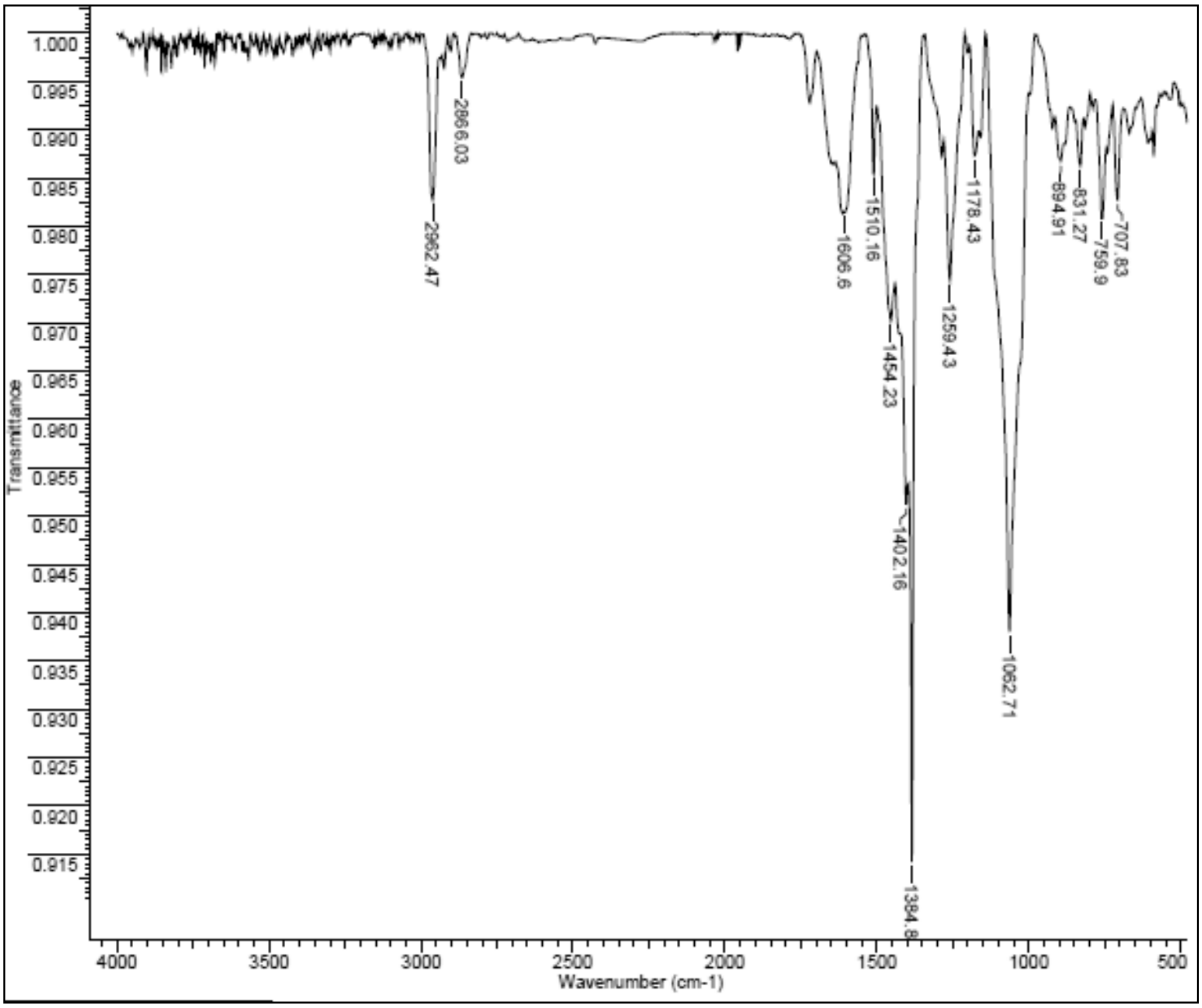

Figure S2. Infrared spectrum for compound **2**.

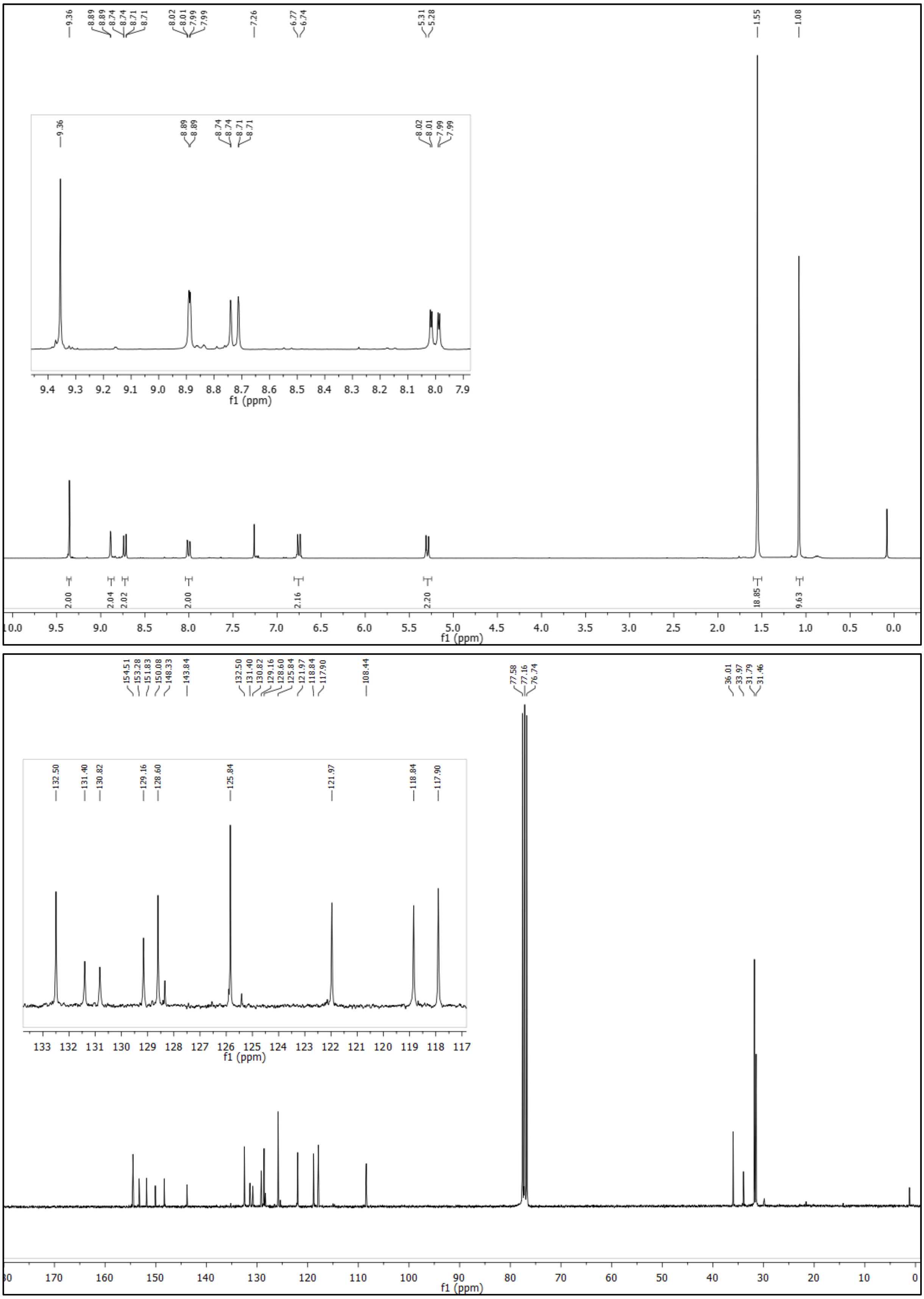

Figure S3. ^{1}H-NMR and ^{13}C-NMR spectra for **2** (regioisomer **a**) in $CDCl_3$ (300 MHz, rt).

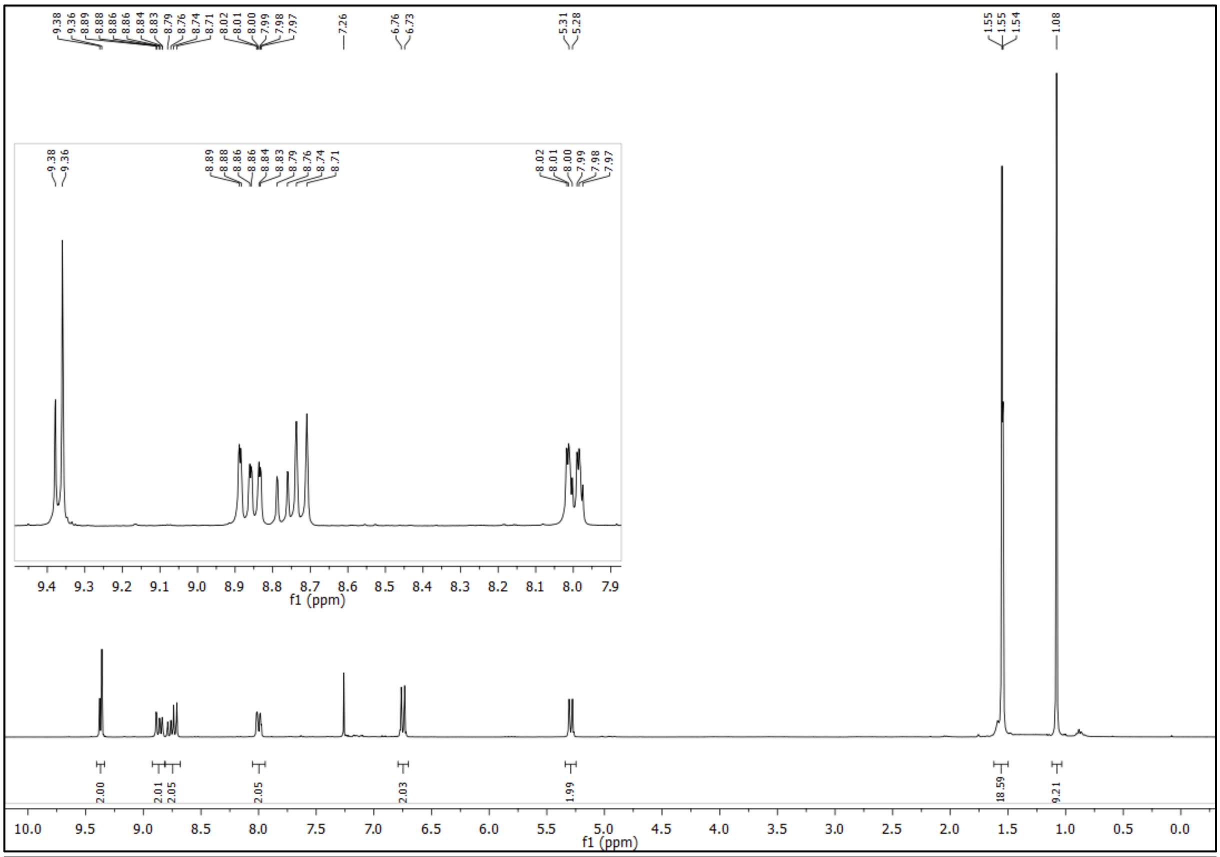

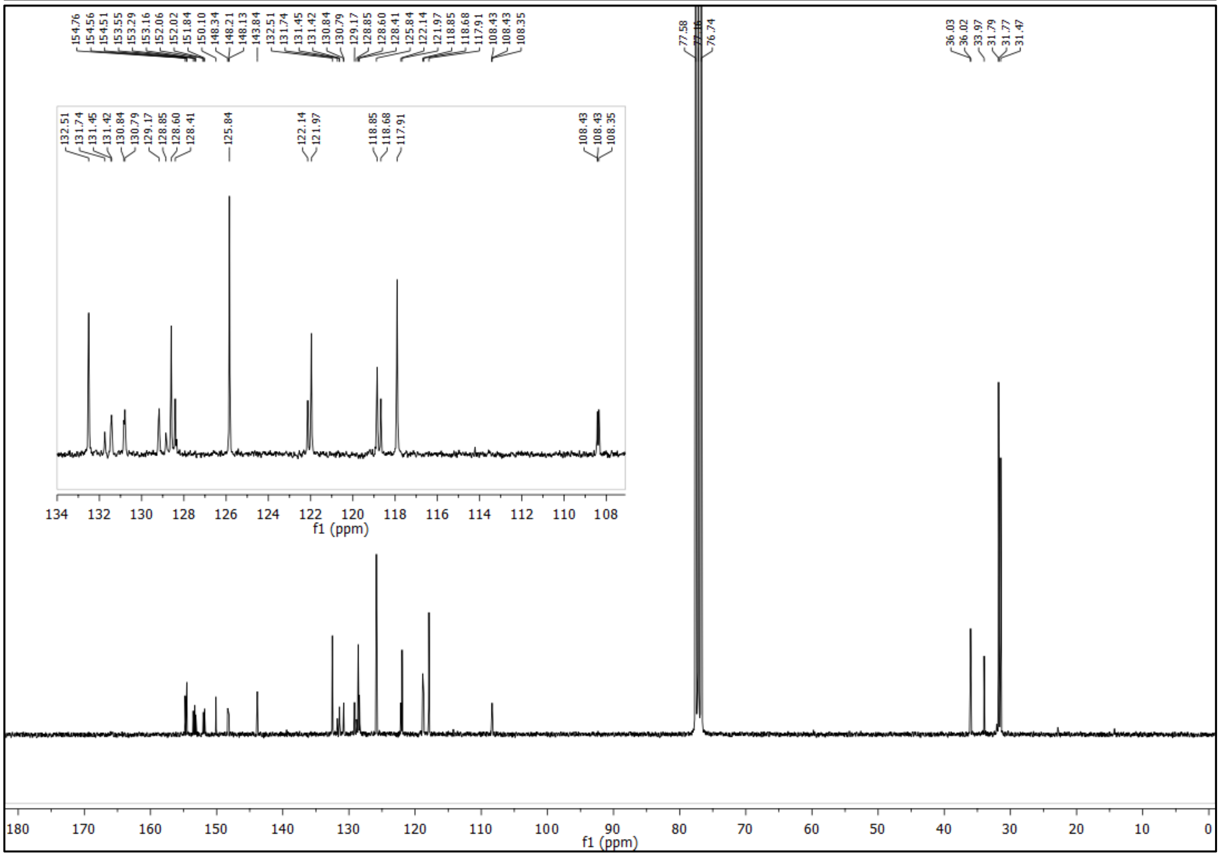

Figure S4. ^{1}H-NMR and ^{13}C-NMR spectra for **2** (mixture of regioisomers **a** and **b**) in $CDCl_3$ (300 MHz, rt).

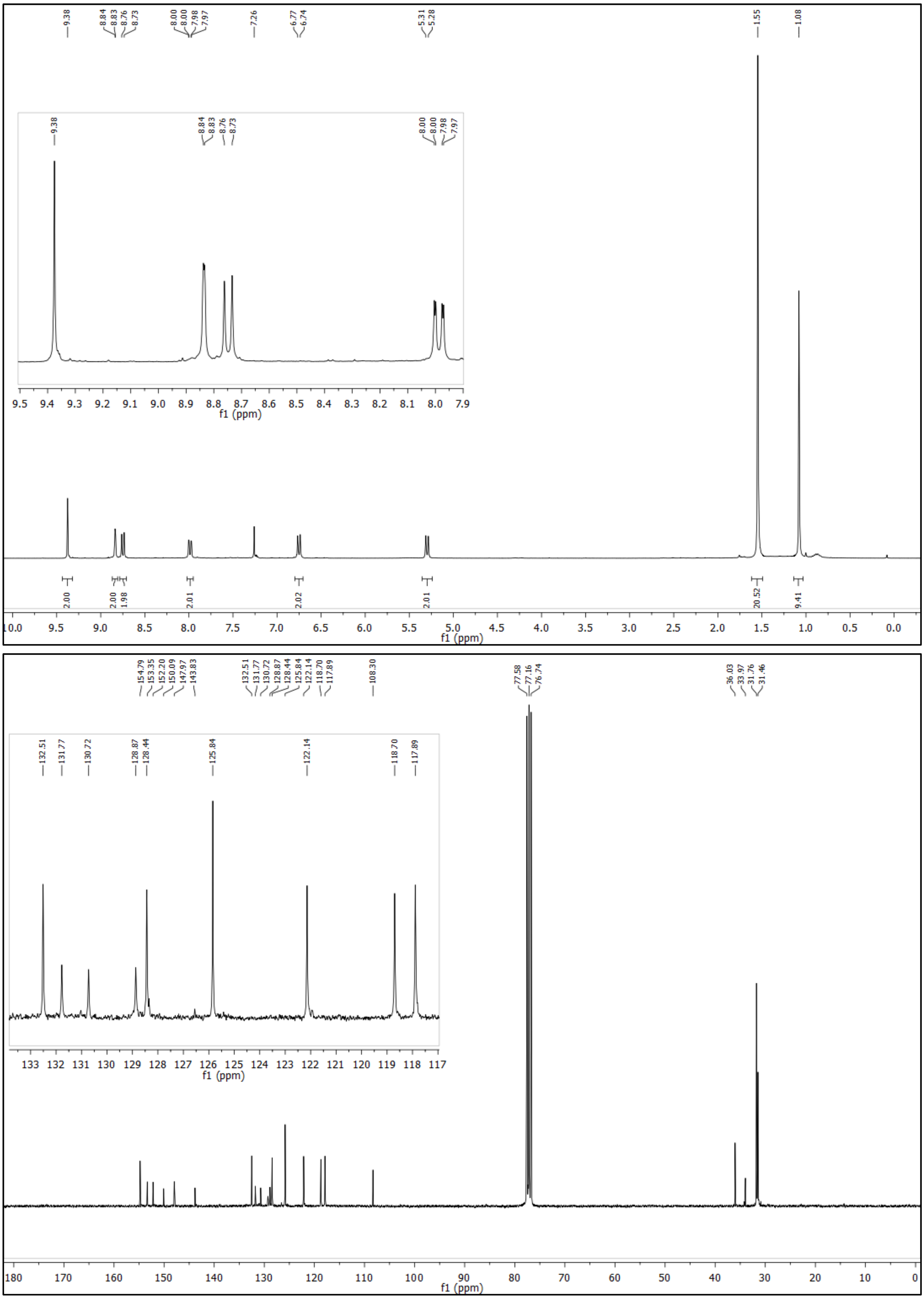

Figure S5. ^{1}H-NMR and ^{13}C-NMR spectra for **2** (regioisomer **c**) in $CDCl_3$ (300 MHz, rt).

Compound 3. To a 5 mL microwave vial, a solution of compound **2** (150 mg, 0.165 mmol) in dry DMF (4 mL), $Zn(CN)_2$ (46 mg, 0.396 mmol) and tetrakis(triphenylphosphine)palladium(0) (38 mg, 0.033 mmol) were added. The reaction tube was sealed and heated to 110 °C under microwave irradiation with a 18 min hold time, and 20 W maximum power input. Water (20 mL) was added and the product was extracted with dichloromethane (3×15 mL). The combined organic extracts were dried over $MgSO_4$ and the solvent was removed by rotary evaporation. The solid residue was purified by column chromatography on silica gel using toluene:THF 200:1 as eluent, yielding product **3** as a purple solid (99 mg, 85%).

Regioisomer **3a**:

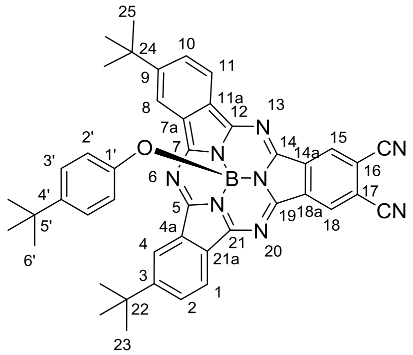

^{1}H-NMR (CDCl$_3$, 300 MHz) δ (ppm): 9.23 (s, 2H, H-15, H-18), 8.87 (d, J_m = 1.6 Hz, 2H, H-4, H-8), 8.72 (d, J_o = 8.3 Hz, 2H, H-1, H-11), 8.06 (dd, J_o = 8.3 Hz, J_m = 1.6 Hz, 2H, H-2, H-10), 6.77 (d, J_o = 8.8 Hz, 2H; H-3'), 5.33 (d, J_o = 8.8 Hz, 2H; H-2'), 1.56 (s, 18H; H-23, H-25), 1.09 (s, 9H; H-6′).
^{13}C-NMR (CDCl$_3$, 75.5 MHz) δ (ppm): 157.1 (C-5, C-7), 155.6 (C-3, C-9), 152.8 (C-12, C-21), 149.8 (C-1'), 145.9 (C-14, C-19), 144.3 (C-4'), 131.4 (C-11a, C-21a), 130.0 (C-4a, C-7a), 129.8 (C-2, C-10), 129.6 (C-14a, C-18a), 128.8 (C-15, C-18), 126.0 (C-3'), 122.4 (C-1, C-11), 119.7 (C-4, C-8), 117.9 (C-2'), 116.3 (C-16, C-17), 112.6 (C≡N), 36.1 (C-22, C-24), 34.0 (C-5'), 31.7 (C-23, C-25), 31.5 (C-6').
UV-Vis (CHCl$_3$): λmax (nm) (log ε (dm^{3}mol^{-1}cm^{-1})) = 598 (4.9), 576 (4.5), 530 (4.3), 514 (4.3), 298 (4.6), 259 (4.6).
FT-IR (film) ν (cm^{-1}): 2961, 2926, 2854, 2230 (C≡N), 1618, 1514, 1464, 1437, 1281, 1257, 1217, 1134, 1107, 1065 (B-O), 833, 762, 712.
MS (MALDI-TOF, DCTB), m/z: 706.4 [M]$^+$ (100%), 557.3 [M – axial group]$^+$ (20%). HRMS (MALDI-TOF, DCTB): calc. for: [M]$^+$: m/z: 706.3342 found 706.3352.

Regioisomer **3b**:

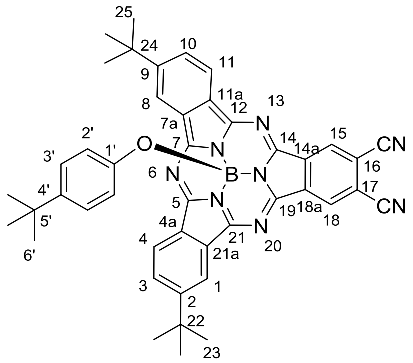

^{1}H-NMR (CDCl$_3$, 300 MHz) δ (ppm): 9.22 (s, 1H, H-18), 9.20 (s, 2H, H-15), 8.86 (d, J_m = 1.6 Hz, 1H, H-8), 8.84 (d, J_m = 1.6 Hz, 1H, H-1), 8.77 (d, J_o = 8.3 Hz, 1H, H-4), 8.77 (d, J_o = 8.3 Hz, 1H, H-11), 8.07 (dd, J_o = 8.3 Hz, J_m = 1.6 Hz, 1H, H-10), 8.03 (dd, J_o = 8.3 Hz, J_m = 1.6 Hz, 1H, H-3), 6.80 (d, J_o = 8.7 Hz, 2H; H-3'), 5.37 (d, J_o = 8.7 Hz, 2H; H-2'), 1.56 (s, 9H; H-25), 1.55 (s, 9H; H-23), 1.10 (s, 9H; H-6′).

^{13}*C-NMR (CDCl$_3$, 75.5 MHz) δ (ppm):* 157.3 (C-7), 156.9 (C-5), 156.4 (C-9), 155.7 (C-2), 153.0, 152.9 (C-12, C-21), 149.8 (C-1'), 145.8, 145.7 (C-14, C-19), 144.3 (C-4'), 132.6 (C-4a), 131.4 (C-11a), 130.0 (C-7a, C-21a), 129.8 (C-10), 129.4 (C-14a, C-18a), 129.1 (C-3), 128.8 (C-18), 128.7 (C-15), 126.0 (C-3'), 122.9 (C-4), 122.4 (C-11), 119.7 (C-8), 119.3 (C-1), 117.9 (C-2'), 116.2 (C-16, C-17), 112.5 (C≡N), 36.2 (C-22), 36.1 (C-24), 34.0 (C-5'), 31.7 (C-23), 31.7 (C-25), 31.4 (C-6').
UV-Vis (CHCl$_3$): λmax (nm) (log ε (dm^{3}mol^{-1}cm^{-1})): 598 (4.9), 576 (4.5), 530 (4.3), 514 (4.3), 298 (4.6), 259 (4.6).
FT-IR (film) ν (cm^{-1}): 2961, 2926, 2854, 2230 (C≡N), 1618, 1514, 1464, 1437, 1281, 1257, 1217, 1134, 1107, 1065 (B-O), 833, 762, 712.
MS (MALDI-TOF, DCTB), m/z: 706.4 [M]$^+$ (100%), 557.3 [M – axial group]$^+$ (20%). HRMS (MALDI-TOF, DCTB): calc. for: [M]$^+$: m/z: 706.3342 found 706.3352.

Regioisomer **3c**:

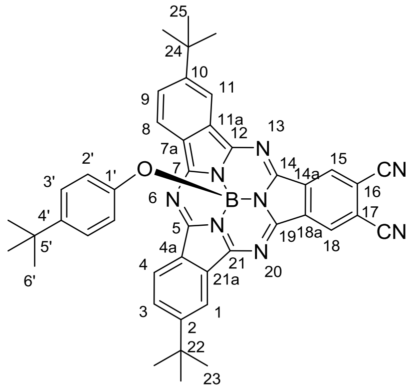

^{1}*H-NMR (CDCl$_3$, 300 MHz) δ (ppm):* 9.25 (s, 2H, H-15, H-18), 8.83 (d, J_m = 1.7 Hz, 2H, H-1, H-11), 8.73 (d, J_o = 8.3 Hz, 2H, H-4, H-8), 8.03 (dd, J_o = 8.3 Hz, J_m = 1.7 Hz, 2H, H-3, H-9), 6.78 (d, J_o = 8.7 Hz, 2H; H-3'), 5.35 (d, J_o = 8.7 Hz, 2H; H-2'), 1.56 (s, 18H; H-23, H-25), 1.09 (s, 9H; H-6′).
^{13}*C-NMR (CDCl$_3$, 75.5 MHz) δ (ppm):* 157.1 (C-12, C-21), 156.4 (C-2, C-10), 153.2 (C-5, C-7), 149.8 (C-1'), 145.6 (C-14, C-19), 144.3 (C-4'), 132.7 (C-4a, C-7a), 129.4 (C-11a, C-21a), 129.1 (C-3, C-9), 128.8 (C-15, C-18), 128.4 (C-14a, C-18a), 126.0 (C-3'), 122.8 (C-4, C-8), 119.3 (C-1, C-11), 117.9 (C-2'), 116.3 (C-16, C-17), 112.5 (C≡N), 36.2 (C-22, C-24), 34.0 (C-5'), 31.7 (C-23, C-25), 31.5 (C-6').
UV-Vis (CHCl$_3$): λmax (nm) (log *ε* (dm^{3}mol^{-1}cm^{-1})) = 598 (4.9), 576 (4.5), 530 (4.3), 514 (4.3), 298 (4.6), 259 (4.6).
FT-IR (film) ν (cm^{-1}): 2961, 2926, 2854, 2230 (C≡N), 1618, 1514, 1464, 1437, 1281, 1257, 1217, 1134, 1107, 1065 (B-O), 833, 762, 712.
MS (MALDI-TOF, DCTB), m/z: 706.4 [M]$^+$ (100%), 557.3 [M – axial group]$^+$ (20%). HRMS (MALDI-TOF, DCTB): calc. for: [M]$^+$: m/z: 706.3342 found 706.3352.

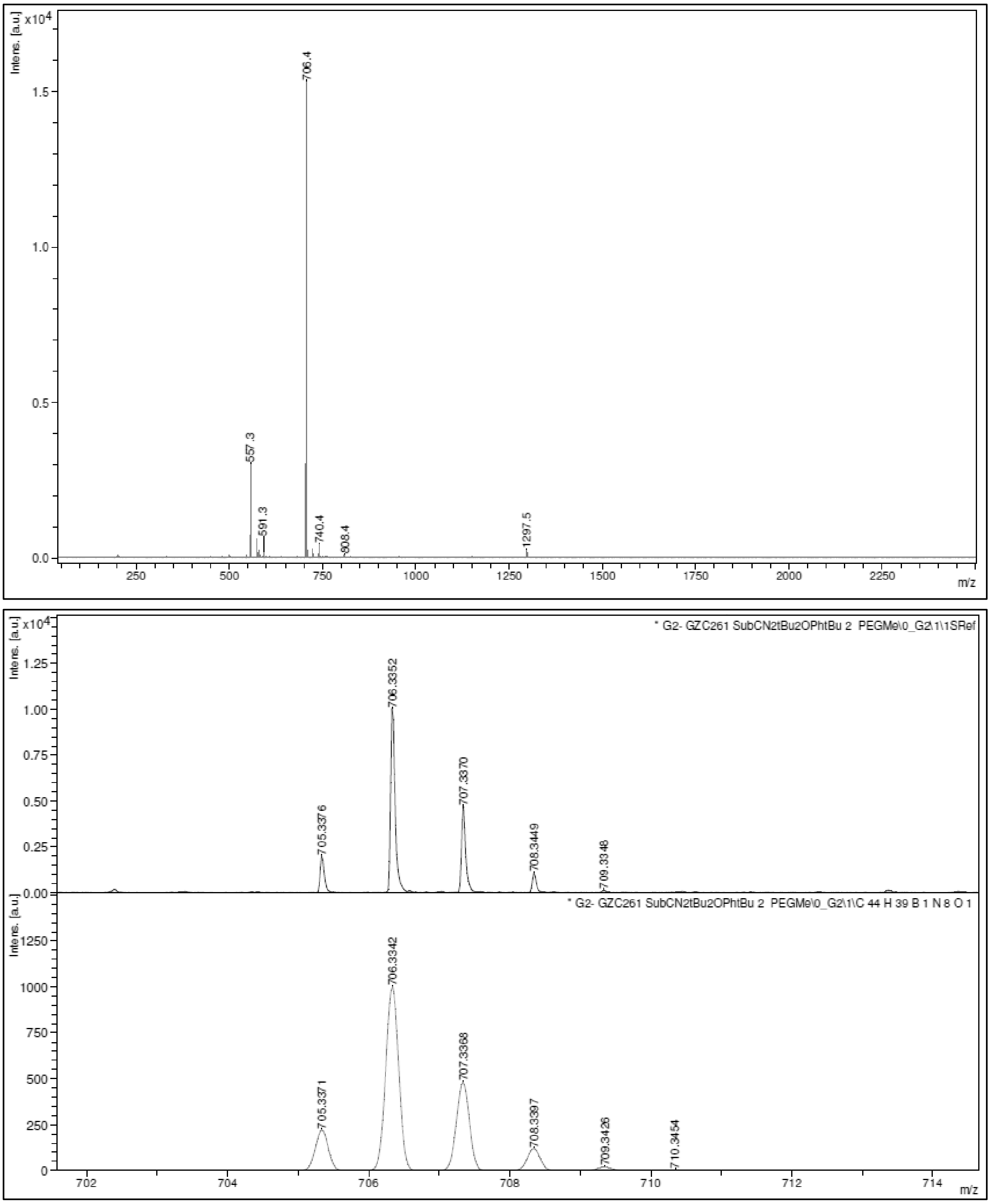

Figure S6. Upper part: MALDI-TOF mass spectrum for **3**. Lower part: A) experimental isotopic pattern and B) calculated isotopic pattern of $[M]^+$ peak of **3**.

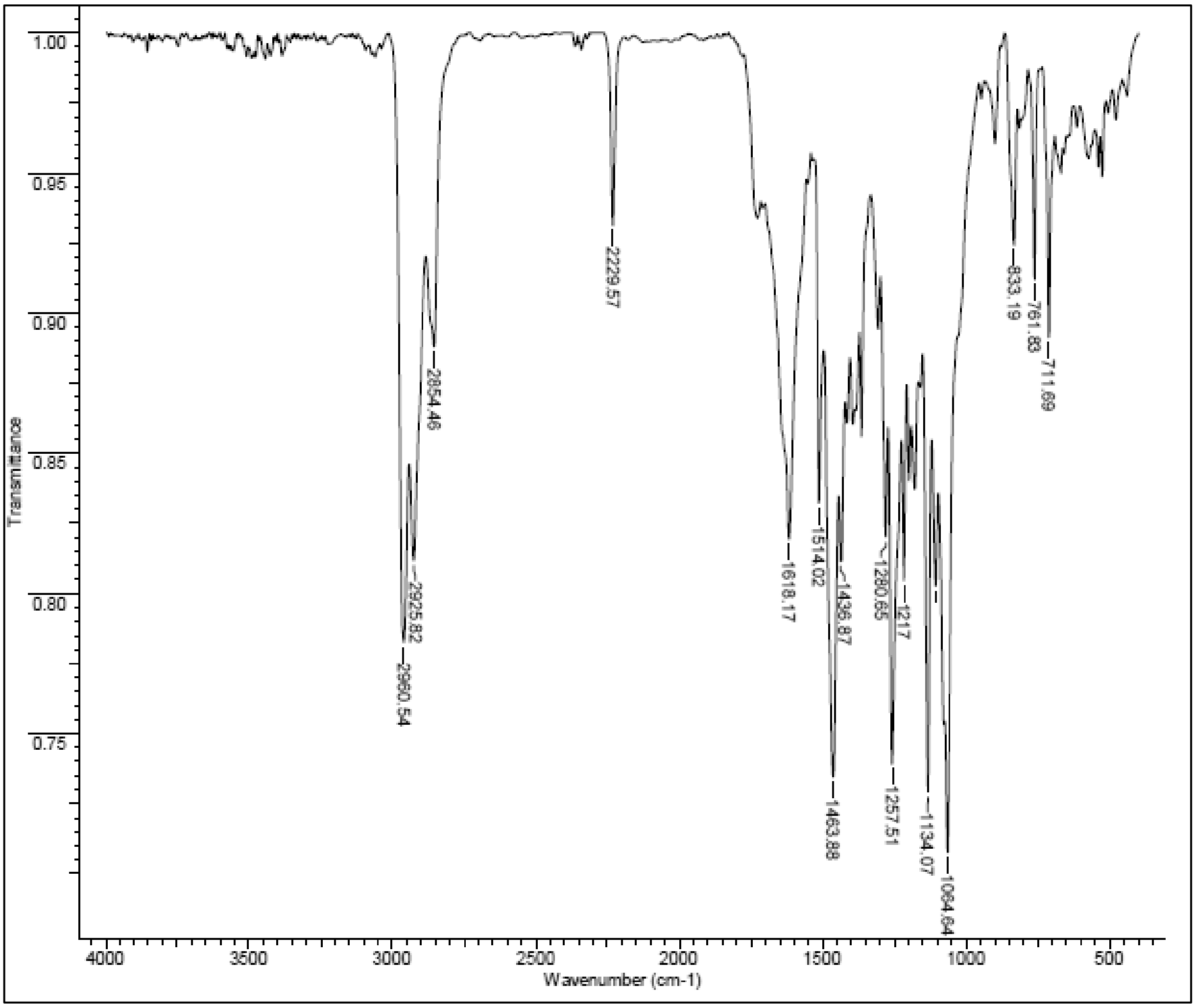

Figure S7. Infrared spectrum for compound **3**.

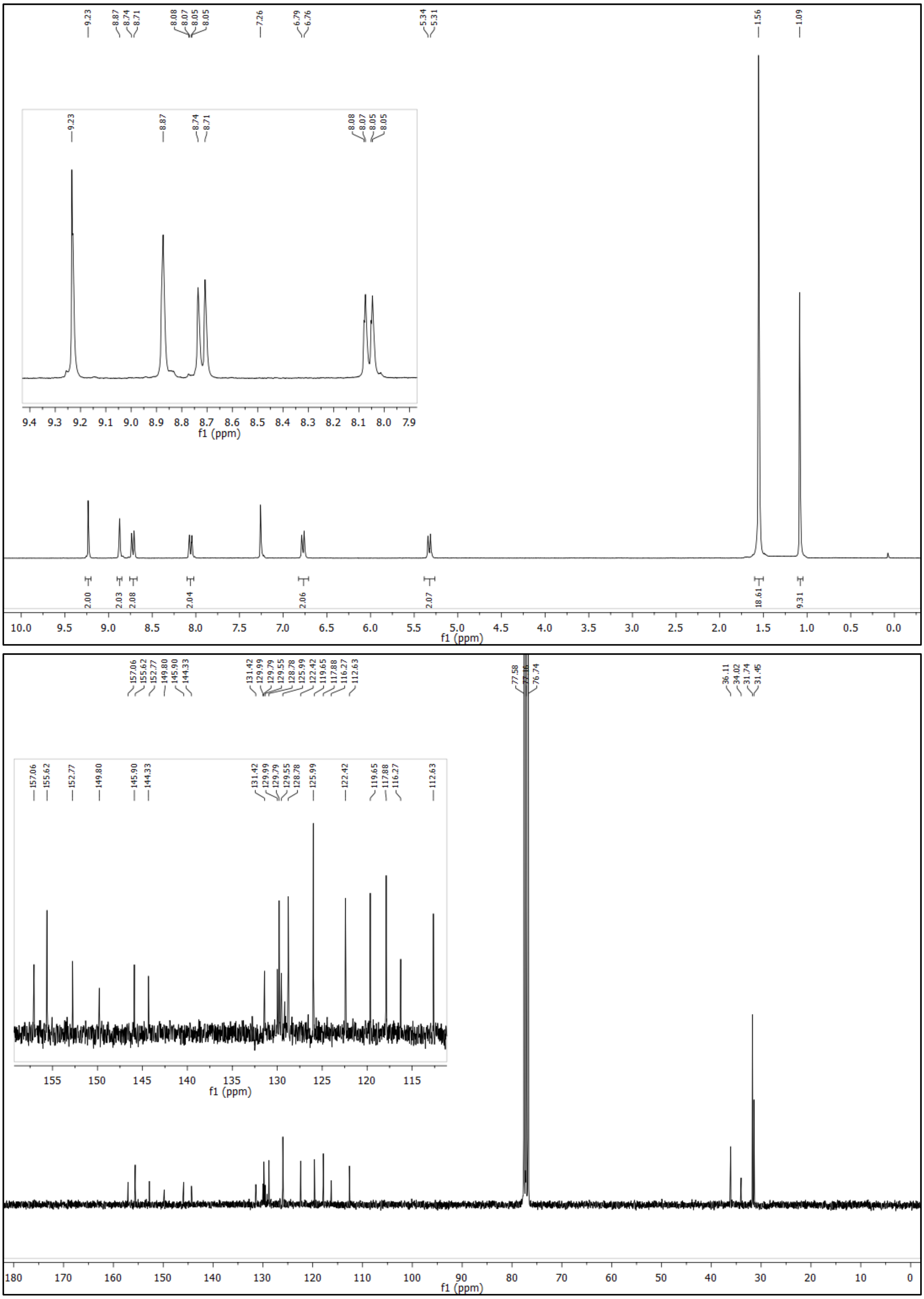

Figure S8. ^{1}H-NMR and ^{13}C-NMR spectra for **3** (regioisomer **a**) in $CDCl_3$ (300 MHz, rt).

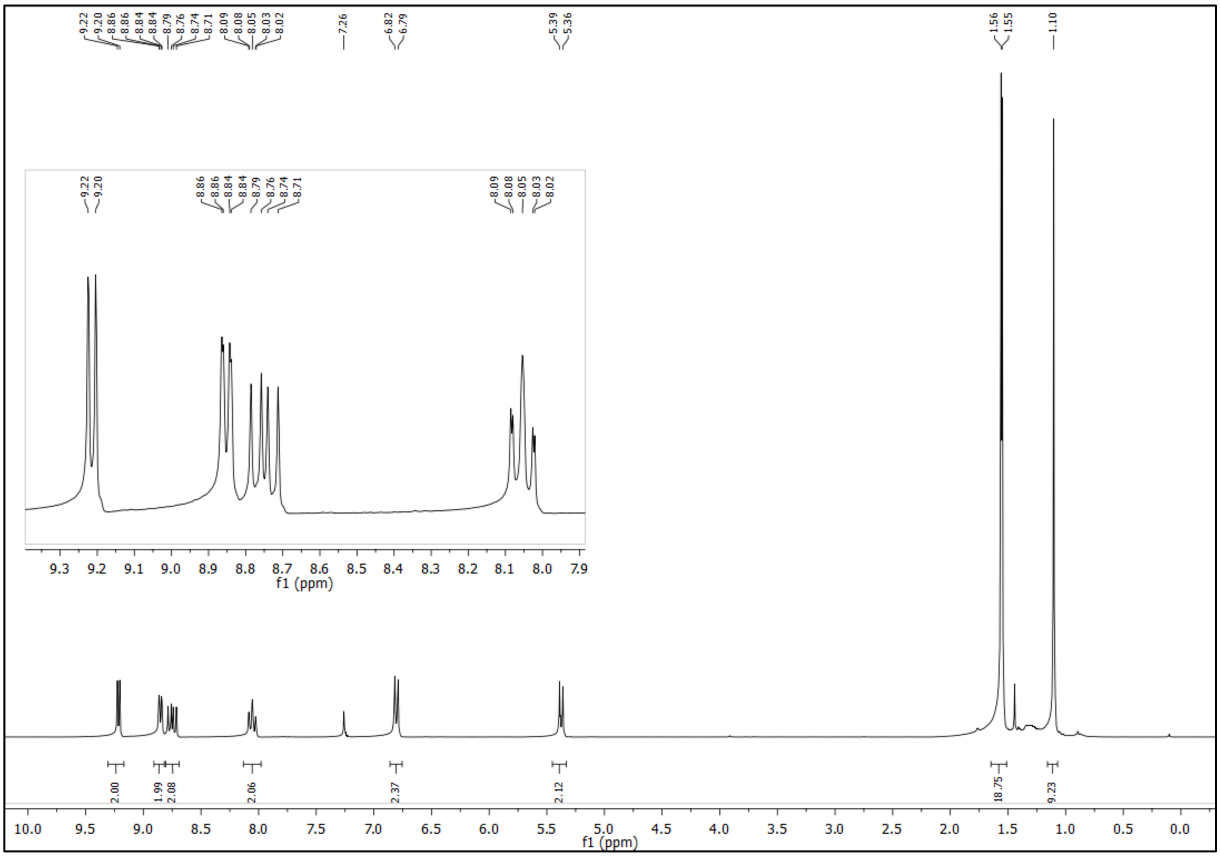

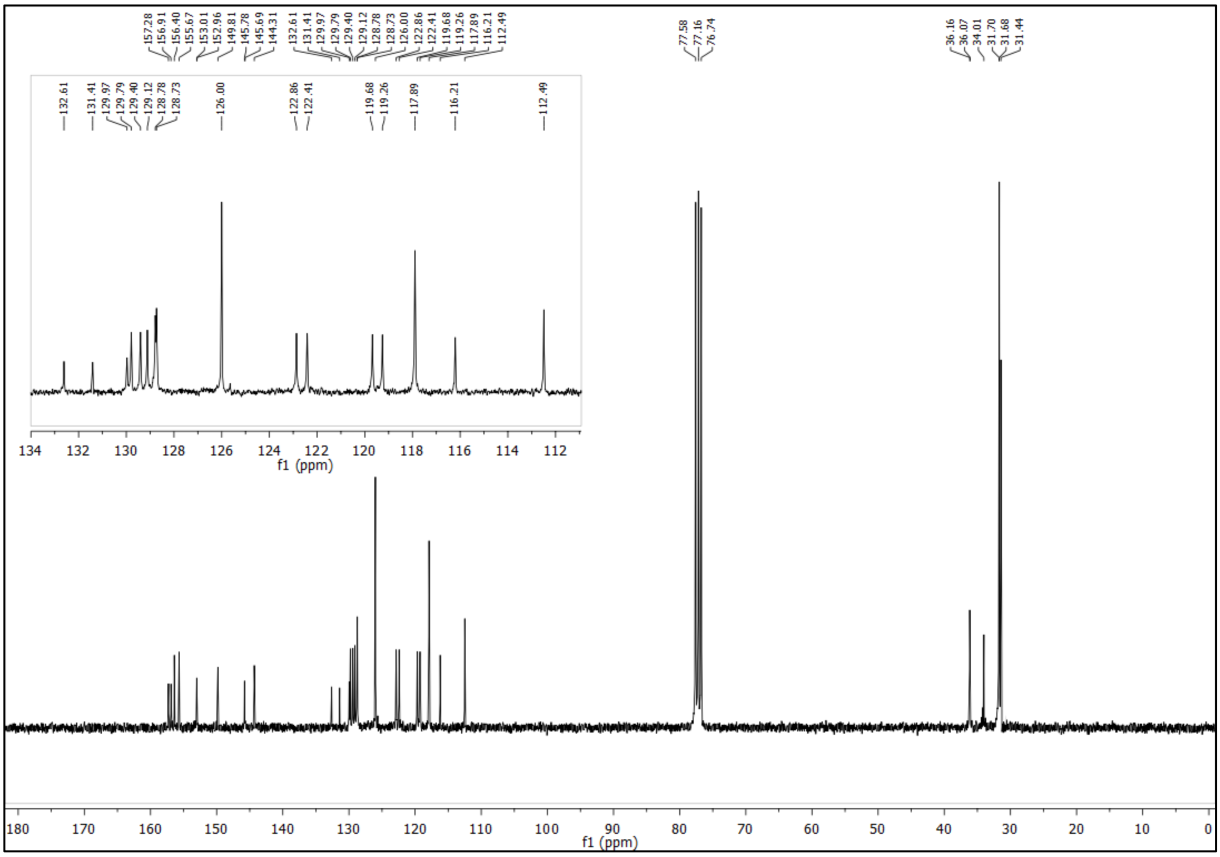

Figure S9. ^{1}H-NMR and ^{13}C-NMR spectra for **3** (regioisomer **b**) in $CDCl_3$ (300 MHz, rt).

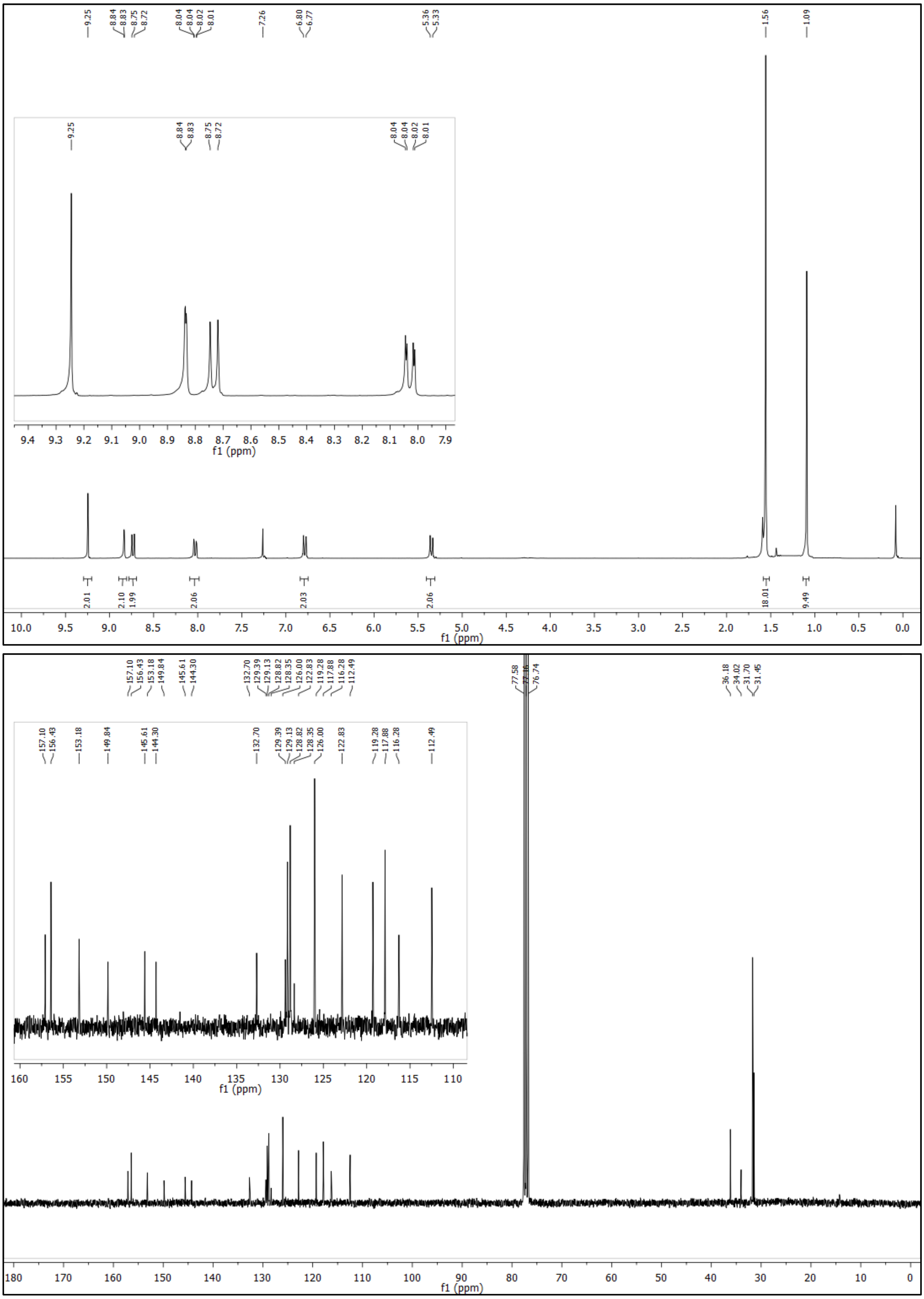

Figure S10. ^{1}H-NMR and ^{13}C-NMR spectra for **3** (regioisomer **c**) in $CDCl_3$ (300 MHz, rt).

SubPc dimer 1. To a 25 ml round-bottom two-neck flask equipped with a reflux condenser and magnetic stirrer, a 1.0 M solution of BCl_3 in p-xylene (600 µL, 0.600 mmol) was added to 4,5-dichlorophthalonitrile (79 mg, 0.400 mmol), under argon atmosphere. The slurry was stirred at room temperature for 5 min. Then, a solution of compound **3** (71 mg, 0.100 mmol) in dry *p*-xylene (1.4 mL) was added and the mixture was heated to reflux (136-138 °C) for 1 h. The crude was cooled down to room temperature and flushed with argon, and solvent was removed by rotary evaporation. The solid residue was purified by column chromatography on silica gel using toluene as eluent, yielding product **1** as a blue solid (17 mg, 16%) as a mixture of *syn*- and *anti*- topoisomers.

Mixture of regio- and topoisomers:

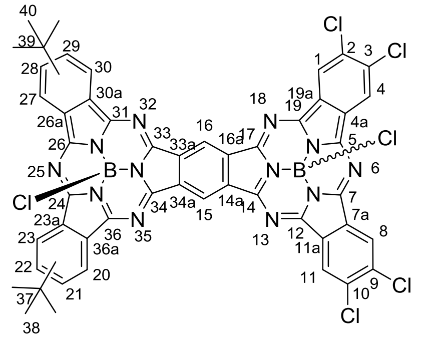

^{1}H-NMR ($CDCl_3$, 500 MHz) δ (ppm): 10.40-10.35 (m, 2H; H-15, H-16), 9.09-8.74 (m, 8H; H-1, H-4, H-8, H-11, H-20, H-23, H-27, H-30), 8.16-8.01 (m, 2H; H-21/H-22, H-28/H-29), 1.64-1.53 (6s, 18H; H-38, H-40).
UV-Vis ($CHCl_3$): λmax (nm) (log ε ($dm^3mol^{-1}cm^{-1}$)): 712 (5.2), 682 (4.7), 654 (4.6), 614 (4.6), 515 (4.1), 439 (4.0), 326 (4.8), 280 (4.7).
FT-IR (film) ν (cm^{-1}): 2963, 2924, 2855, 1725, 1611, 1459, 1385, 1281, 1225, 1175, 1127, 1096, 1025, 975, 890, 793, 703.
MS (MALDI-TOF, DCTB), m/z: 1032.1 $[M]^+$. HRMS (MALDI-TOF, DCTB): calc. for: $[M]^+$: m/z: 1032.1021 found 1032.1009.

Regioisomer **1a**, mixture of *syn*- and *anti*- topoisomers:

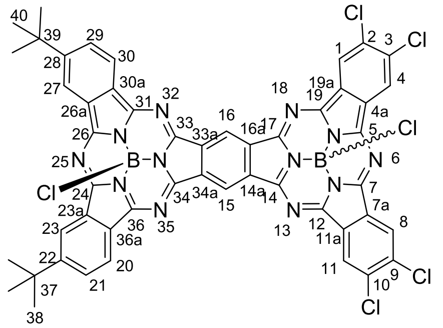

^{1}H-NMR ($CDCl_3$, 500 MHz) δ (ppm): 10.38 (s, 2H; H-15, H-16), 9.08, 9.00 (2s, 2H, H-23, H-27), 9.00, 8.93 (2s, 4H, H-1, H-4, H-8, H-11), 8.90, 8.82 (2d, J_o = 8.3 Hz, 2H, H-20, H-30), 8.14, 8.05 (2dd, J_o = 8.3 Hz, J_m = 1.7 Hz, 2H; H-21, H-29), 1.56-1.54 (m, 18H; H-38, H-40).
UV-Vis ($CHCl_3$): λmax (nm) (log ε ($dm^3mol^{-1}cm^{-1}$)): 712 (5.2), 682 (4.7), 654 (4.6), 614 (4.6), 515 (4.1), 439 (4.0), 326 (4.8), 280 (4.7).
MS (MALDI-TOF, DCTB), m/z: 1032.1 $[M]^+$. HRMS (MALDI-TOF, DCTB): calc. for: $[M]^+$: m/z: 1032.1021 found 1032.1009.

Regioisomer **1b**, mixture of *syn*- and *anti*- topoisomers:

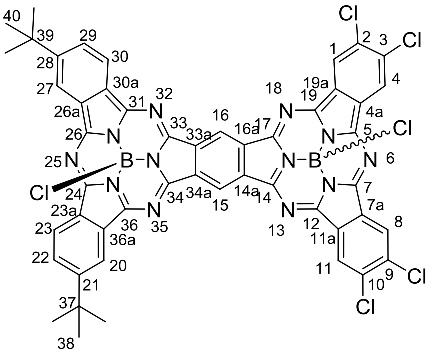

^{1}H-NMR ($CDCl_3$, 500 MHz) δ (ppm): 10.39, 10.38, 10.37, 10.36 (4d, J_p = 0.8 Hz, 2H; H-15, H-16), 9.06, 8.99, 8.89 (3s, 4H, H-1, H-4, H-8, H-11), 9.02, 8.99, 8.97, 8.95 (4d, J_m = 1.7 Hz, 2H, H-20, H-27), 8.92, 8.88, 8.81, 8.79 (4d, J_o = 8.3 Hz, 2H, H-23, H-30), 8.14, 8.10, 8.04, 8.02 (4dd, J_o = 8.3 Hz, J_m = 1.7 Hz, 2H; H-22, H-29), 1.64, 1.61, 1.57, 1.53 (4s, 18H; H-38, H-40).
UV-Vis ($CHCl_3$): λmax (nm) (log ε ($dm^3mol^{-1}cm^{-1}$)): 712 (5.2), 682 (4.7), 654 (4.6), 614 (4.6), 515 (4.1), 439 (4.0), 326 (4.8), 280 (4.7).
MS (MALDI-TOF, DCTB), m/z: 1032.1 $[M]^+$. HRMS (MALDI-TOF, DCTB): calc. for: $[M]^+$: m/z: 1032.1021 found 1032.1009.

Regioisomer **1c**, mixture of *syn*- and *anti*- topoisomers:

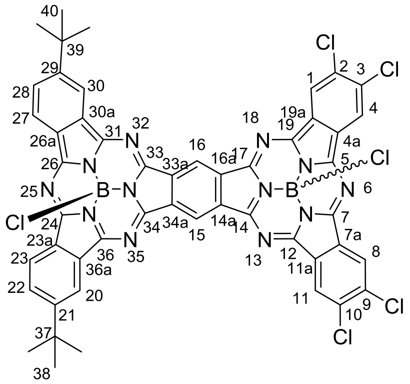

^{1}H-NMR ($CDCl_3$, 500 MHz) δ (ppm): 10.40, 10.39 (2s, 2H; H-15, H-16), 9.06, 9.04 (2d, J_m = 1.7 Hz, 2H, H-20, H-30), 8.99, 8.95, 8.90 (3s, 4H, H-1, H-4, H-8, H-11), 8.86, 8.76 (2d, J_o = 8.3 Hz, 2H, H-23, H-27), 8.10, 8.02 (2dd, J_o = 8.3 Hz, J_m = 1.7 Hz, 2H; H-22, H-28), 1.58-1.54 (m, 18H; H-38, H-40).
UV-Vis ($CHCl_3$): λmax (nm) (log ε ($dm^3mol^{-1}cm^{-1}$)): 712 (5.2), 682 (4.7), 654 (4.6), 614 (4.6), 515 (4.1), 439 (4.0), 326 (4.8), 280 (4.7).
MS (MALDI-TOF, DCTB), m/z: 1032.1 $[M]^+$. HRMS (MALDI-TOF, DCTB): calc. for: $[M]^+$: m/z: 1032.1021 found 1032.1009.

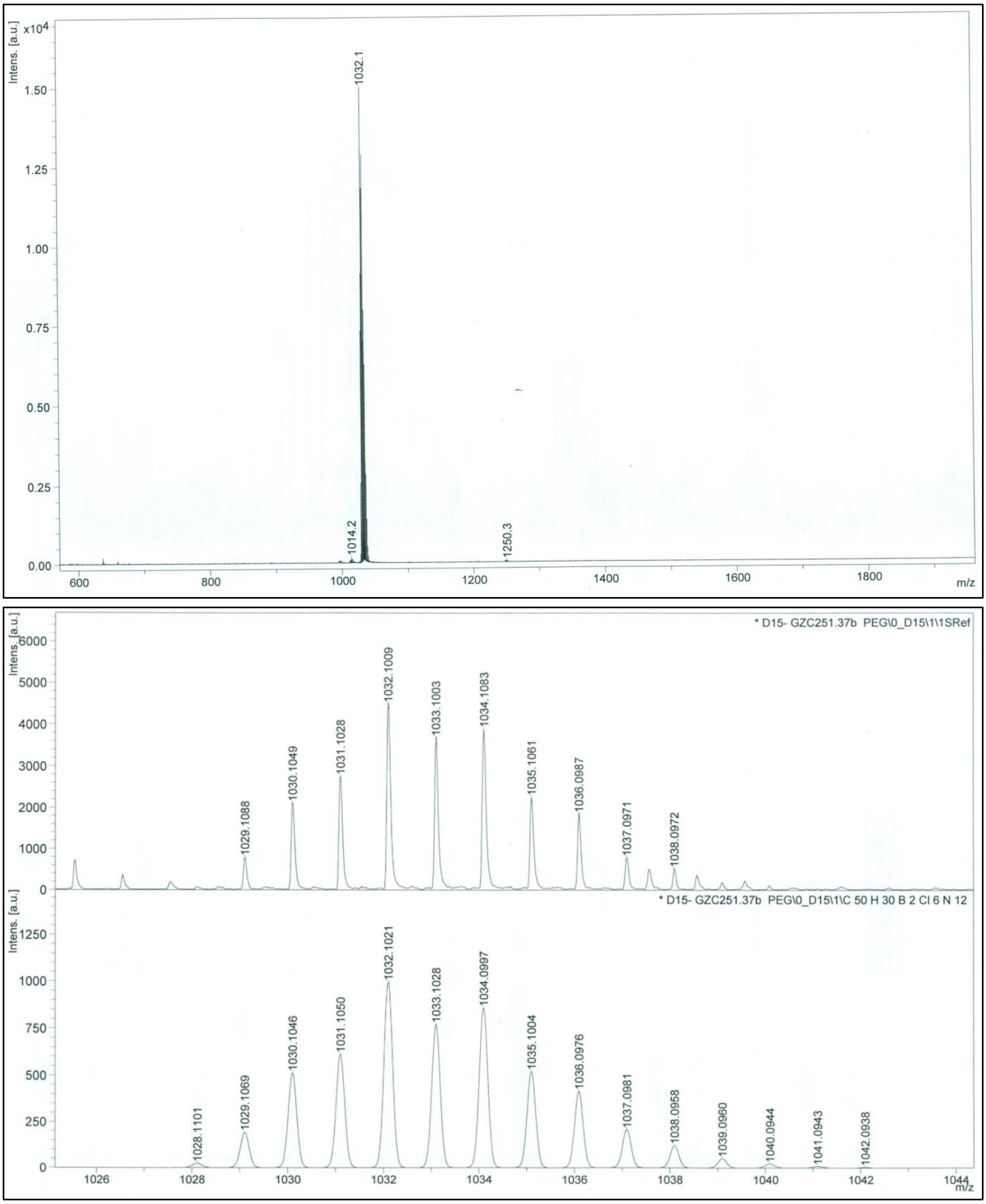

Figure S11. Upper part: MALDI-TOF mass spectrum for **1**. Lower part: A) experimental isotopic pattern and B) calculated isotopic pattern of $[M]^+$ peak of **1**.

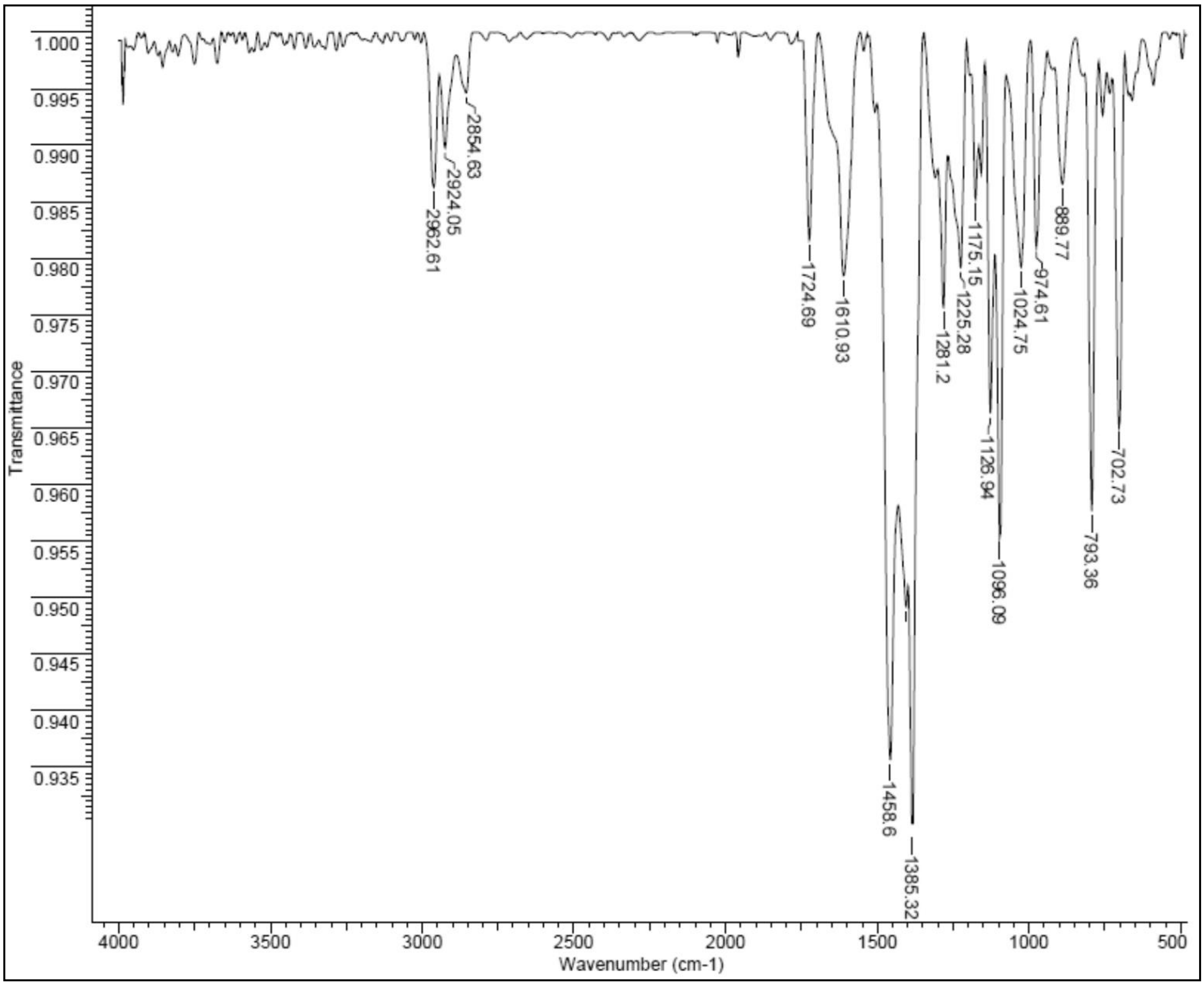

Figure S12. Infrared spectrum for compound **1**.

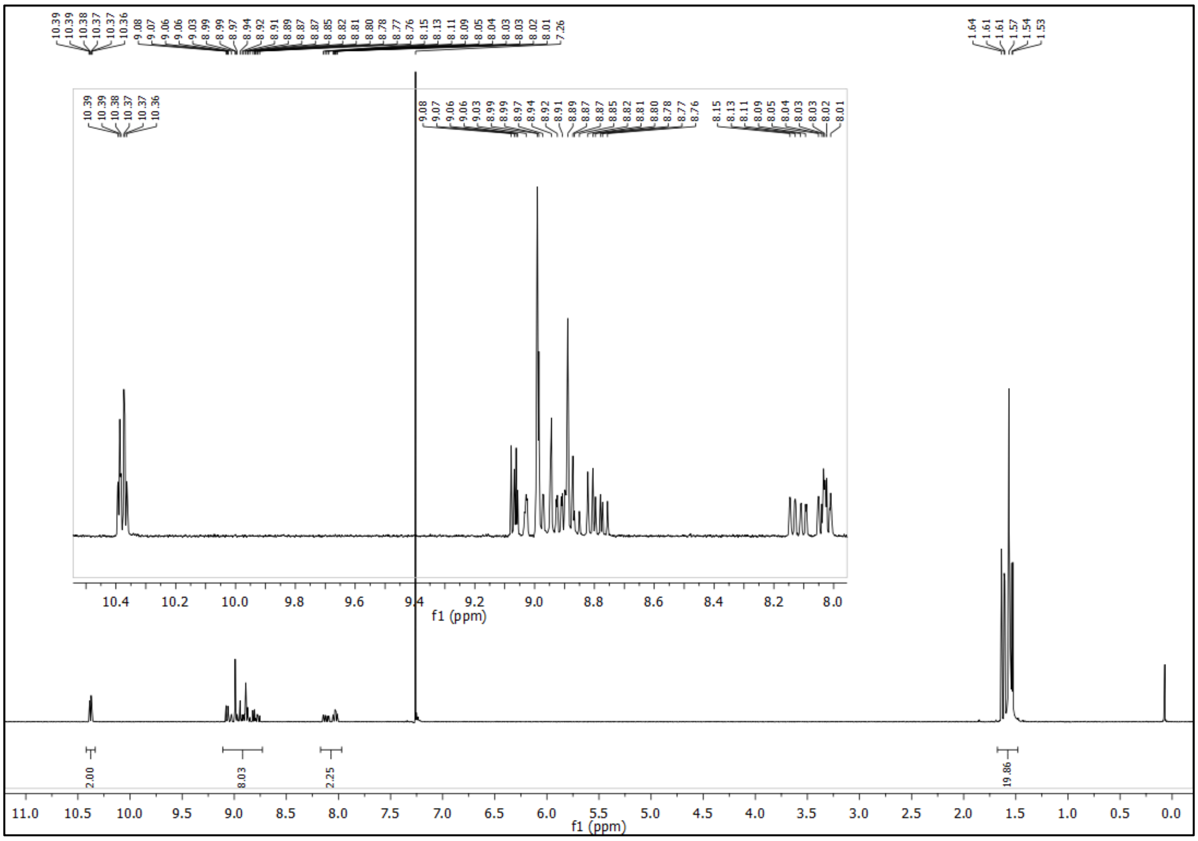

Figure S13. ^{1}H-NMR spectrum for **1** (mixture of regio- and topoisomers) in $CDCl_3$ (500 MHz, rt).

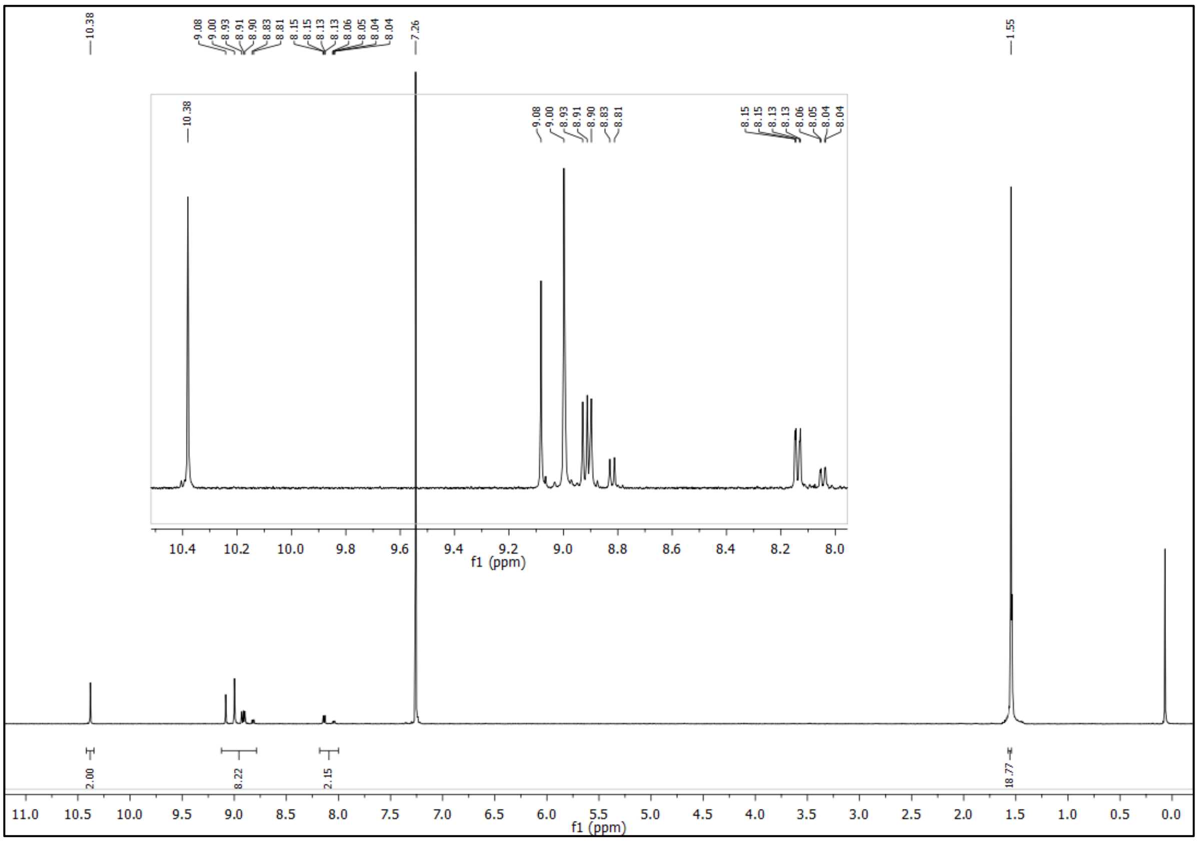

Figure S14. ^{1}H-NMR spectrum for **1** (regioisomer **a**, mixture of syn and anti topoisomers) in $CDCl_3$ (500 MHz, rt).

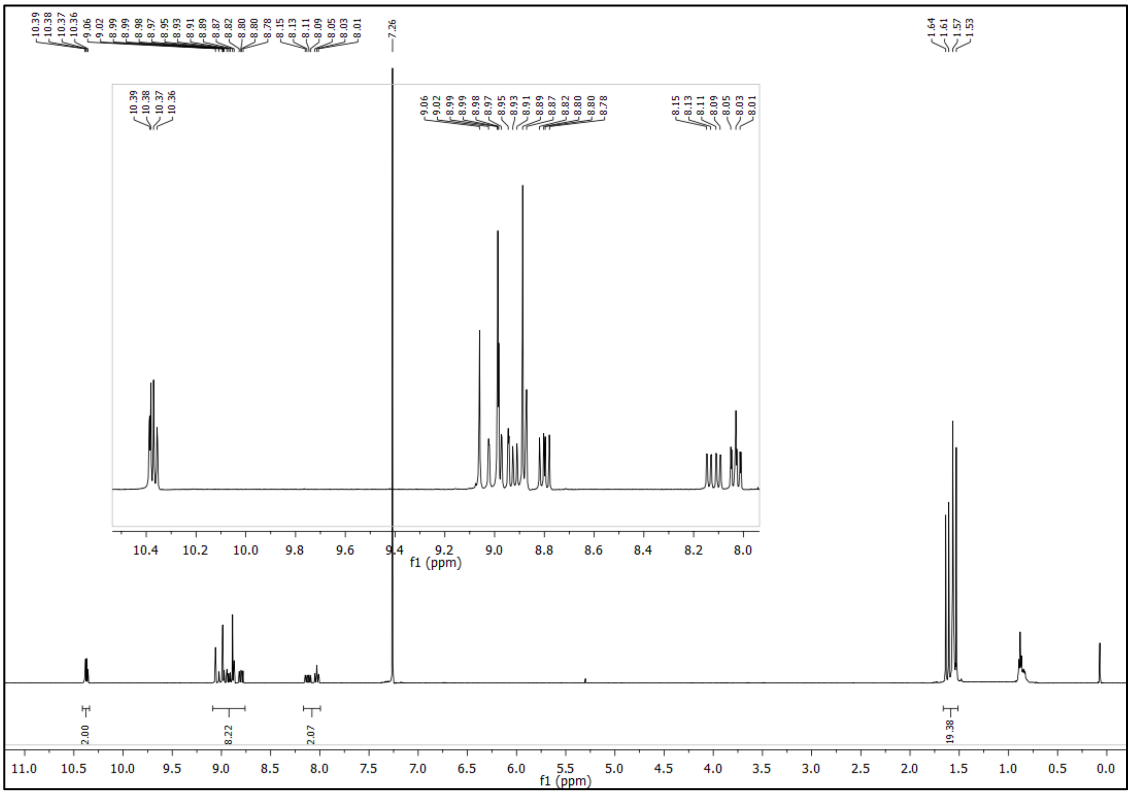

Figure S15. ^{1}H-NMR spectrum for **1** (regioisomer **b**, mixture of syn and anti topoisomers) in $CDCl_3$ (500 MHz, rt).

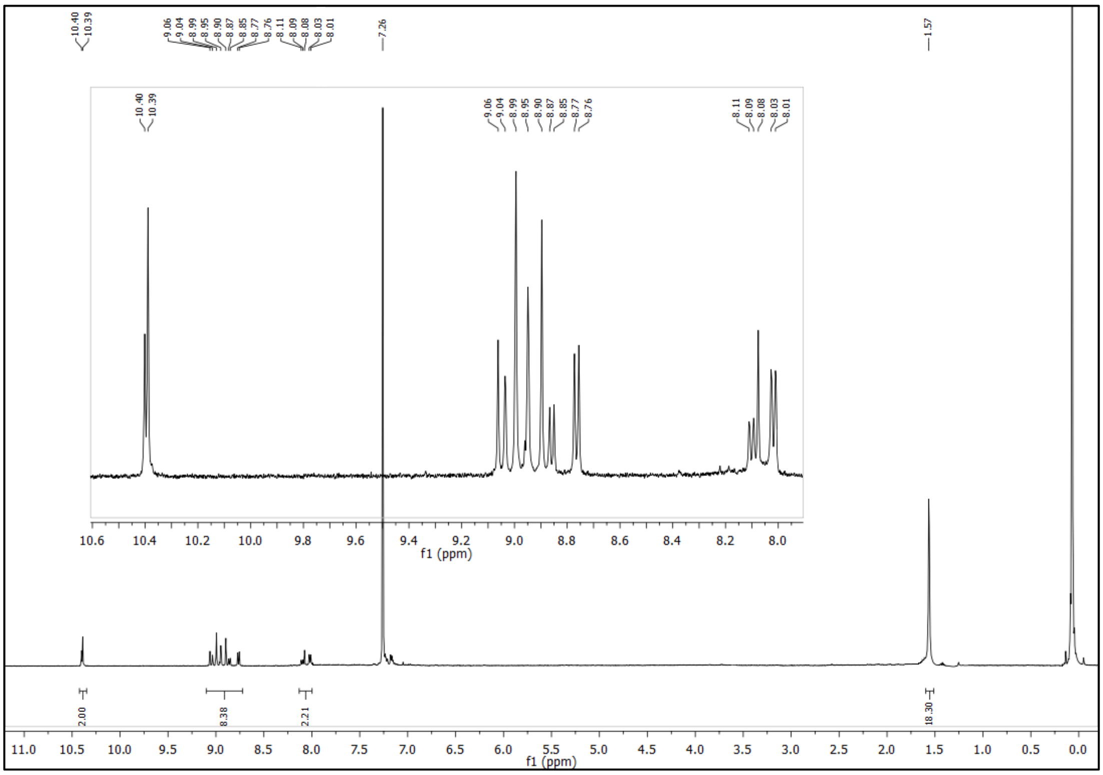

Figure S16. ^{1}H-NMR spectrum for **1** (regioisomer **c**, mixture of syn and anti topoisomers) in $CDCl_3$ (500 MHz, rt).

SubPc dimers 4a and 4b. In a 25 ml round-bottom two-neck flask equipped with a reflux condenser and magnetic stirrer, compound **1** (10 mg, 0.010 mmol) and 4-*tert*-butylphenol (15 mg, 0.100 mmol) were solved in dry toluene (1 mL). The mixture was refluxed for 2 hours. After cooling down to room temperature, the excess of phenol was removed by washing the crude with a 3:1 methanol/water solution. The crude product was purified by column chromatography on silica gel using toluene as eluent, yielding topoisomers **4a** (5 mg, 45%) and **4b** (5 mg, 45%) separately (90% overall yield).

Topoisomer **4a**, mixture of regioisomers:

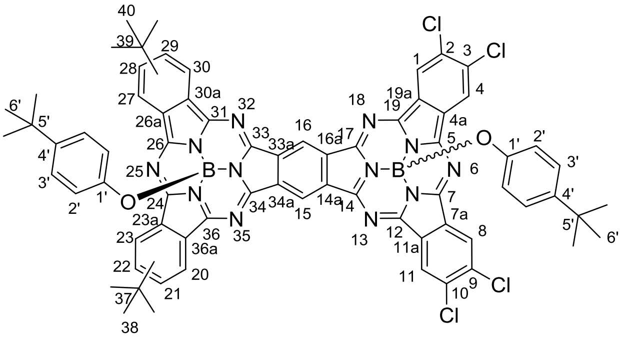

^{1}H-NMR ($CDCl_3$, 500 MHz) δ (ppm): 10.33-10.29 (m, 2H; H-15, H-16), 9.04-8.80 (m, 8H; H-1, H-4, H-8, H-11, H-20, H-23, H-27, H-30), 8.11-8.03 (m, 2H; H-21/H-22, H-28/H-29), 6.71-6.66 (2d, J_o = 8.7 Hz, $J_{o'}$ = 8.7 Hz, 4H; H-3'), 5.32-5.24 (m, 4H; H-2'), 1.63-1.57 (3s, 18H; H-38, H-40), 1.02-0.99 (m, 18H; H-6´).
UV-Vis ($CHCl_3$): λmax (nm) (log ε ($dm^3mol^{-1}cm^{-1}$)): 710 (5.2), 679 (4.7), 651 (4.6), 610 (4.6), 515 (4.1), 437 (4.0), 327 (4.8).
MS (MALDI-TOF, DCTB), m/z: 1260.3 $[M]^+$. HRMS (MALDI-TOF, DCTB): calc. for: $[M]^+$: m/z: 1260.3584 found 1260.3588.

Topoisomer **4b**, mixture of regioisomers:

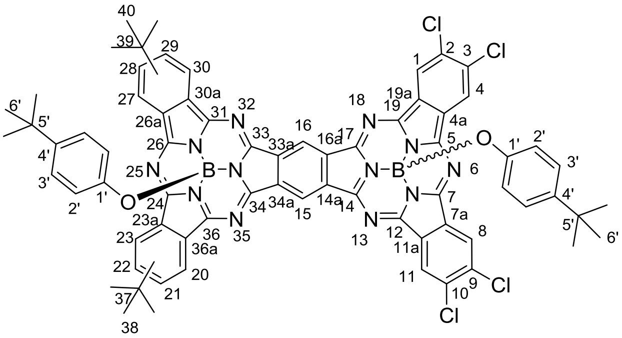

^{1}H-NMR ($CDCl_3$, 500 MHz) δ (ppm): 10.30-10.25 (m, 2H; H-15, H-16), 8.98-8.70 (m, 8H; H-1, H-4, H-8, H-11, H-20, H-23, H-27, H-30), 8.02-7.94 (m, 2H; H-21/H-22, H-28/H-29), 6.88-6.81 (2d, J_o = 8.7 Hz, $J_{o'}$ = 8.7 Hz, 4H; H-3'), 5.46-5.41 (m, 4H; H-2'), 1.57-1.50 (3s, 18H; H-38, H-40), 1.15-1.13 (2s, 18H; H-6´).
UV-Vis ($CHCl_3$): λmax (nm) (log ε ($dm^3mol^{-1}cm^{-1}$)): 710 (5.2), 679 (4.7), 651 (4.6), 610 (4.6), 515 (4.1), 437 (4.0), 327 (4.8).
MS (MALDI-TOF, DCTB), m/z: 1260.3 $[M]^+$. HRMS (MALDI-TOF, DCTB): calc. for: $[M]^+$: m/z: 1260.3584 found 1260.3588.

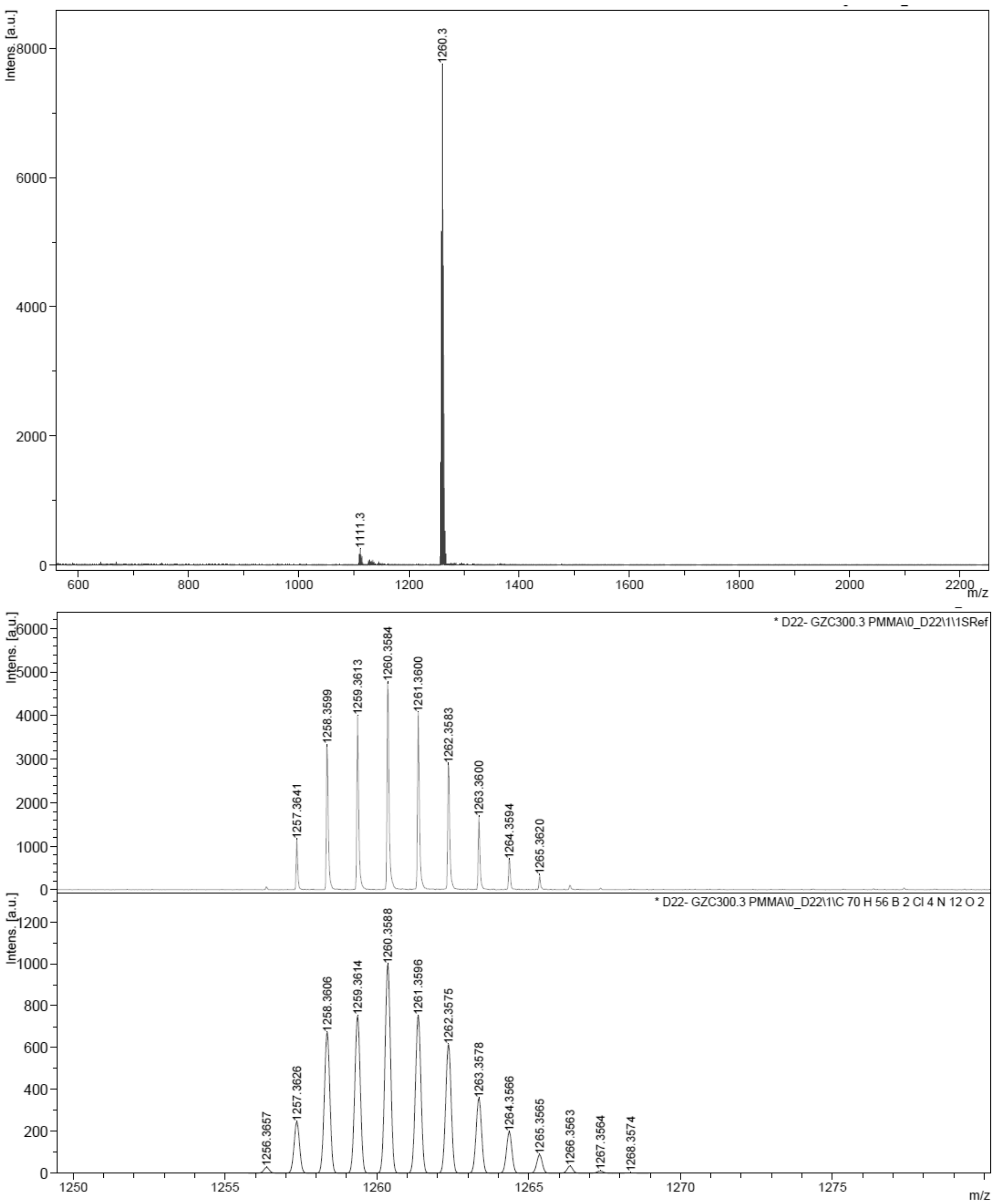

Figure S17. Upper part: MALDI-TOF mass spectrum for **4**. Lower part: A) experimental isotopic pattern and B) calculated isotopic pattern of $[M]^+$ peak of **4**.

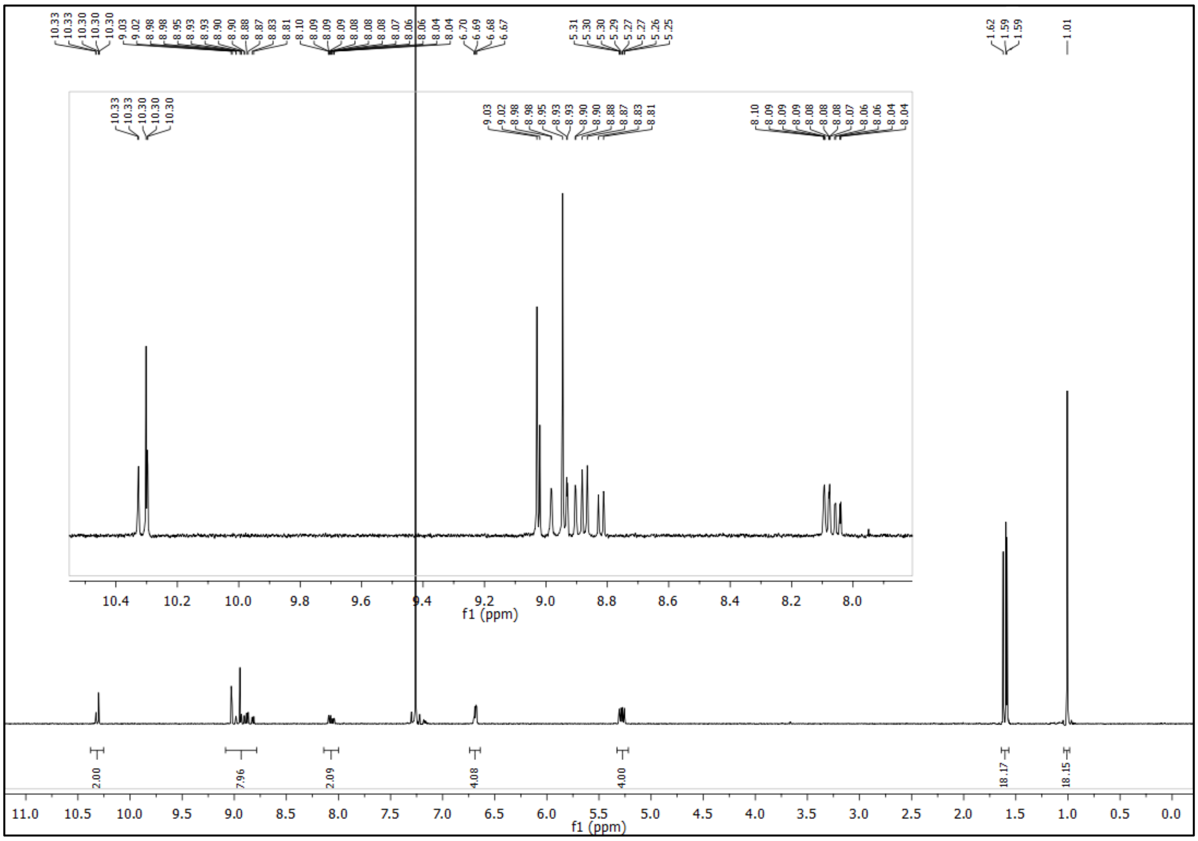

Figure S18. ^{1}H-NMR spectrum for **4a** (mixture of regioisomers) in $CDCl_3$ (500 MHz, rt).

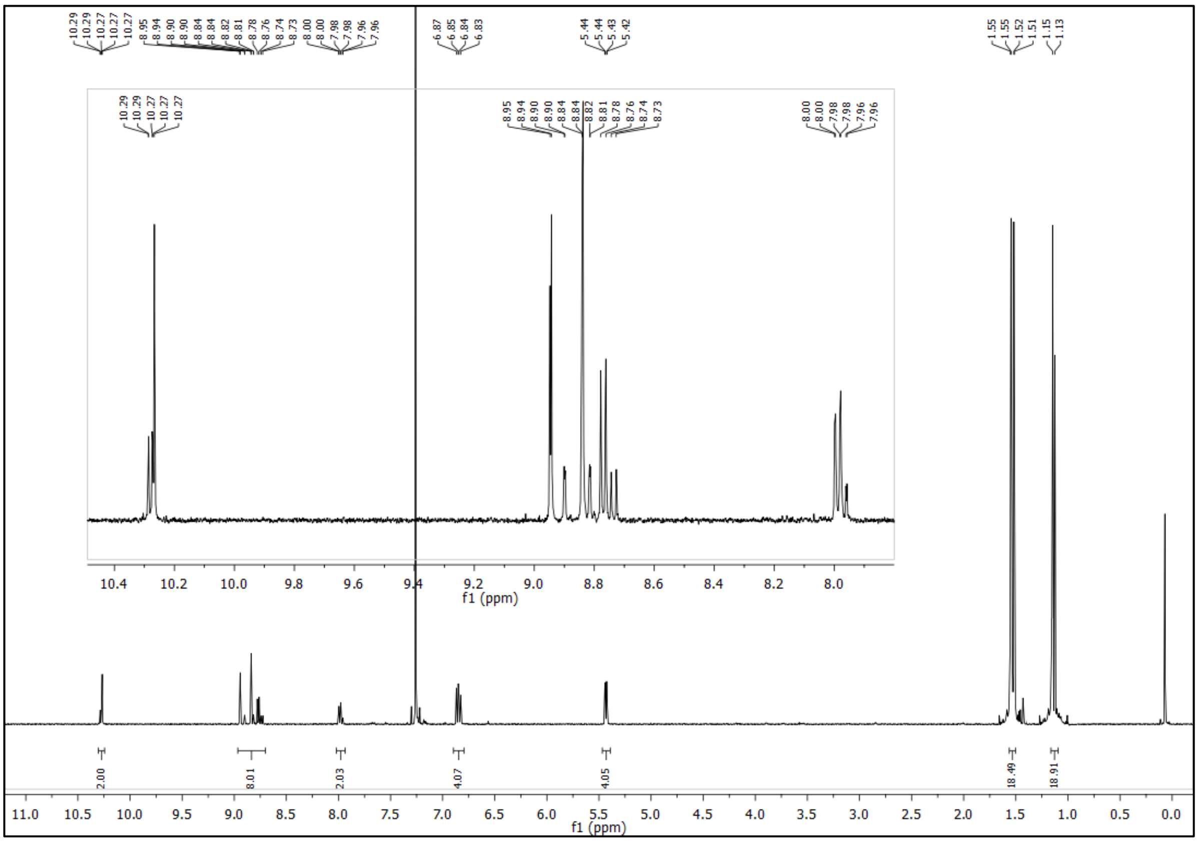

Figure S19. ^{1}H-NMR spectrum for **4b** (mixture of regioisomers) in $CDCl_3$ (500 MHz, rt).

4. **UV-vis absorption spectra, fluorescence emission spectra and electrochemical characterization of compounds 1-4.**

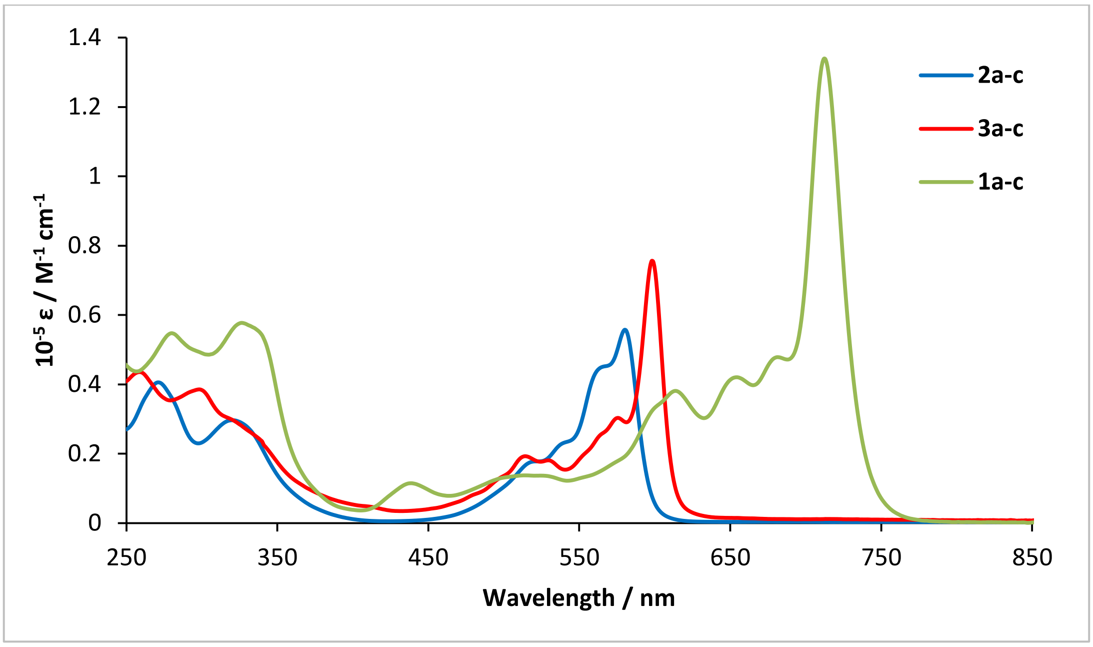

Figure S20. UV-vis absorption spectra of compounds **1-3** in chloroform.

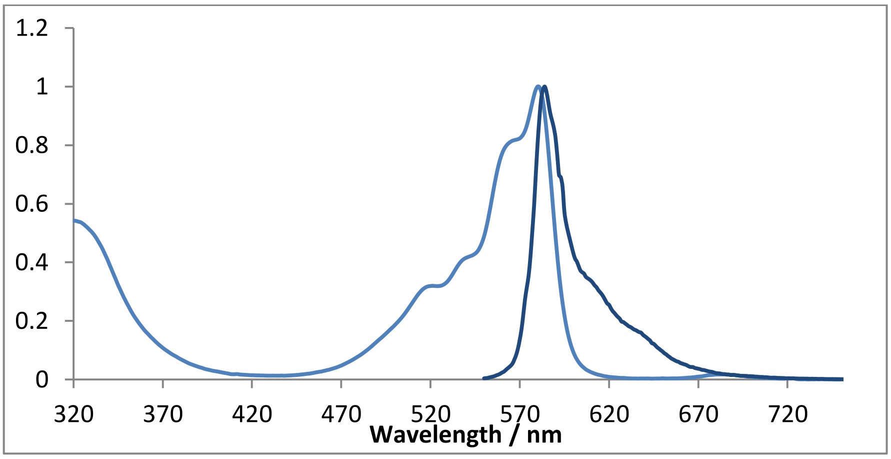

Figure S21. Normalized UV-vis absorption spectra (light blue) and normalized fluorescence emission spectra (exc. wavelength = 545 nm) (dark blue) of compound **2** in chloroform.

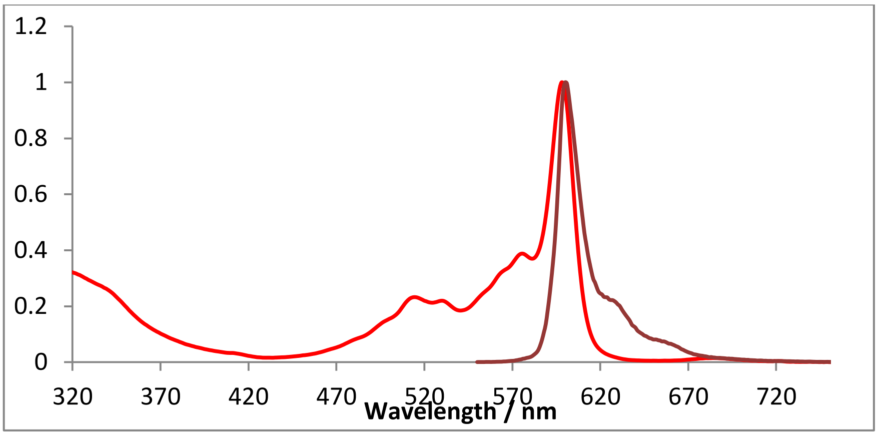

Figure S22. Normalized UV-vis absorption spectra (light red) and normalized fluorescence emission spectra (exc. wavelength = 560 nm) (dark red) of compound **3** in chloroform.

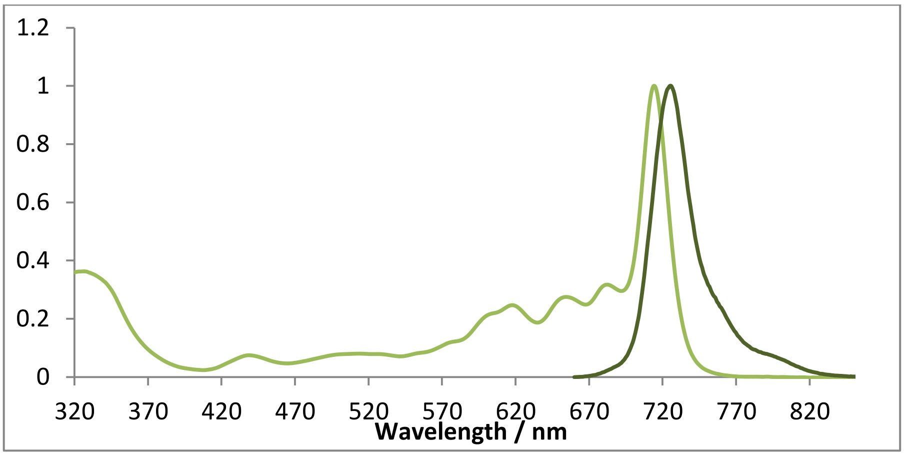

Figure S23. Normalized UV-vis absorption spectra (light green) and normalized fluorescence emission spectra (exc. wavelength = 620 nm) (dark green) of compound **1** in chloroform.

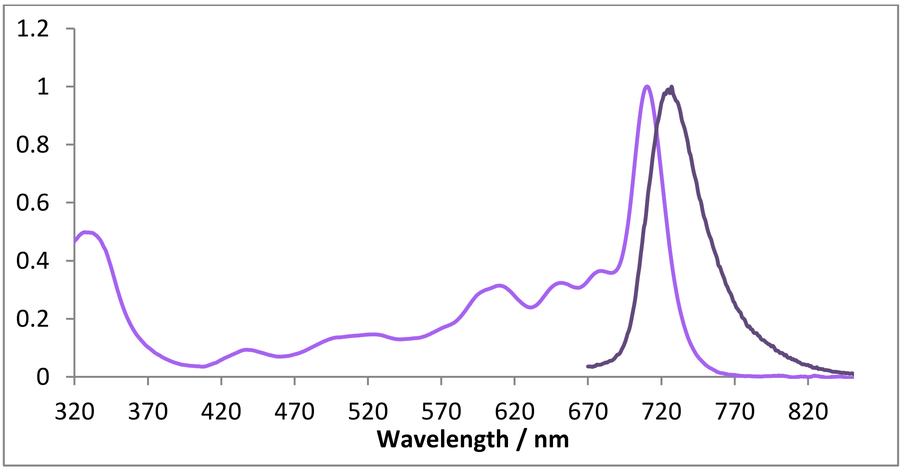

Figure S24. Normalized UV-vis absorption spectra (light green) and normalized fluorescence emission spectra (exc. wavelength = 620 nm) (dark green) of compounds **4a-b** in chloroform.

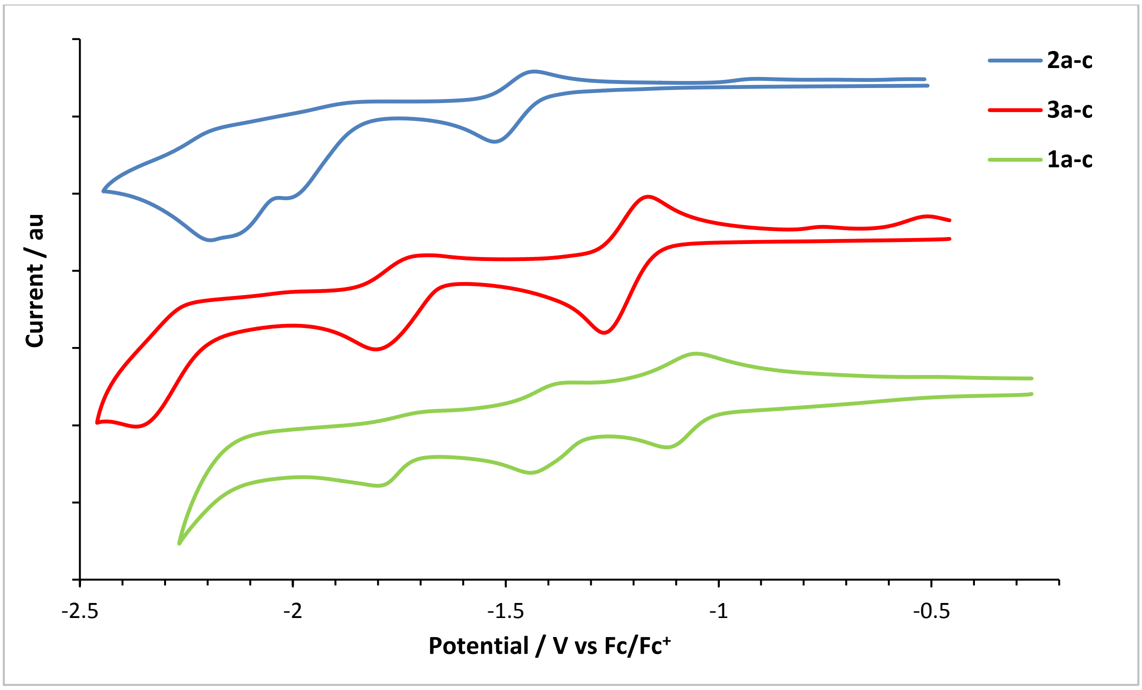

Figure S25. Cyclic voltammograms (anodic window) of compounds **1-3** (referred to Fc/Fc+) in THF.

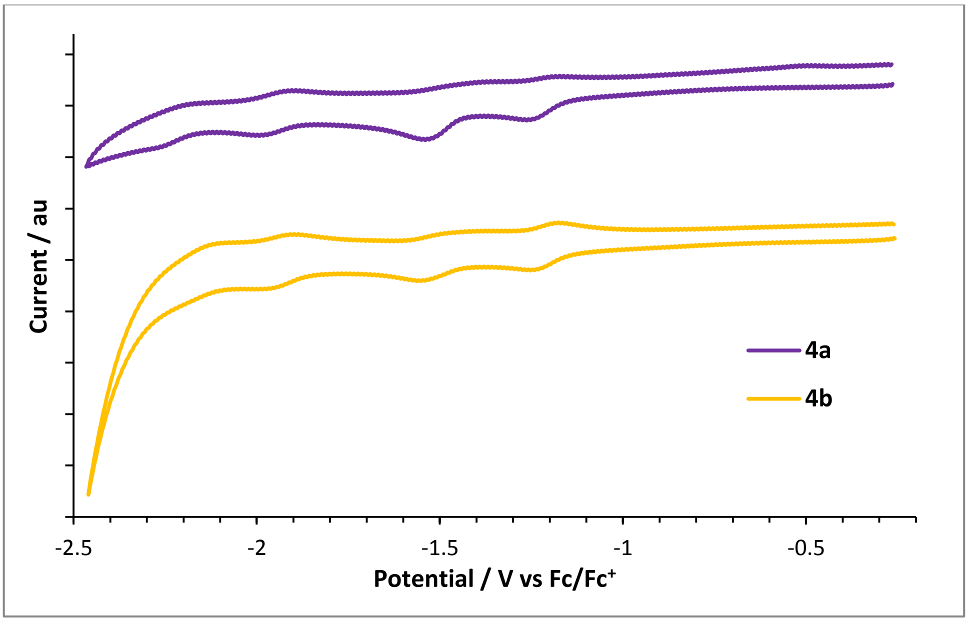

Figure S26. Cyclic voltammograms (anodic window) of compounds **4a** and **4b** (referred to Fc/Fc+) in THF.

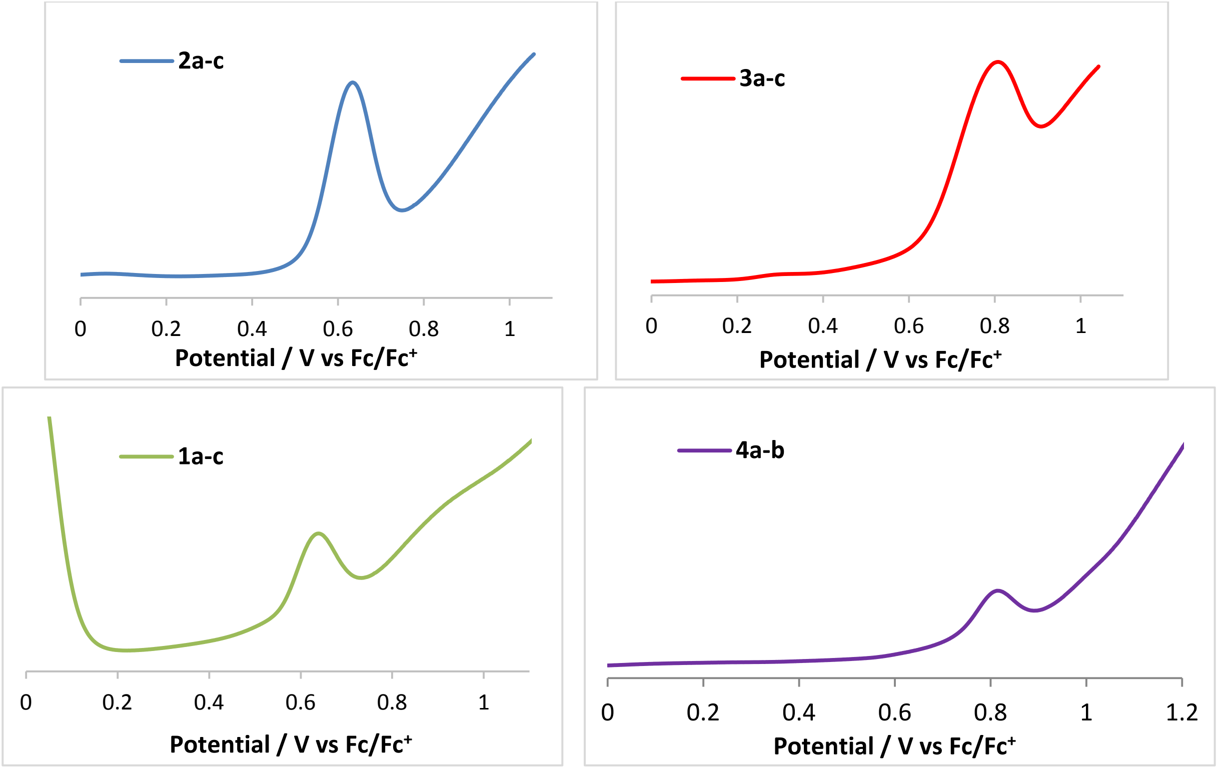

Figure S27. Square Wave Voltammetry plots (cathodic window) of compounds **1-4** (referred to Fc/Fc+) in THF.

	λ_{max} (nm)[a]	log (ε)[a]	λ_{em} (nm)[b]	$E^1_{1/2,red}$ (mV)[c]	$E^2_{1/2,red}$ (mV)[c]	$E^3_{1/2,red}$ (mV)[c]	E^1_{ox} (mV)[c,d]	E_{LUMO} (eV)[e]	$E_{0\text{-}0}$ (eV)[f]	E_{HOMO} (eV)
**1**	712	5.13	725	-1088	-1392	-1801	640	-3.71	1.73	-5.44
**2**	580	4.75	584	-1481	-2008[g]	-	668	-3.32	2.13	-5.75
**3**	598	4.88	600	-1221	-1744	-2289[g]	806	-3.58	2.07	-5.65
**4a**	710	5.13	727	-1215	-1487	-1953	589	-3.59	1.72	-5.31
**4b**	710	5.13	727	-1217	-1478	-1948	569	-3.58	1.73	-5.31

[a] 10^{-5} M, in chloroform. [b] 10^{-7} M, in chloroform. [c] [10^{-3} M] in THF vs Fc/Fc^+, Pt working electrode, Pt counter electrode, 20 ºC, 0.1 $TBAPF_6$, scan rate = 100 mVs^{-1}. [d] Peak oxidation potential by square wave voltammetry. [e] Calculated with respect to ferrocene, E_{HOMO}: -4.8 eV. [f] Estimated from the intersection between the absorption and emission spectra. [g] Since these reduction processes are irreversible, only cathodic peak potentials are reported.

Table S1. UV-vis and Fluorescence Emission Spectroscopies data, Electrochemical Reduction Potentials from Cyclic Voltammetry and Oxidation Potentials from Square Wave Voltammetry, Lowest Unoccupied Molecular Orbital Energy Level, Optical Band Gap Energy and Highest Occupied Molecular Orbital Energy Level of compounds **1-4**.

5. **UV-vis absorption spectra, fluorescence emission spectra and transient absorption spectra of compounds 1, 5 and 6.**

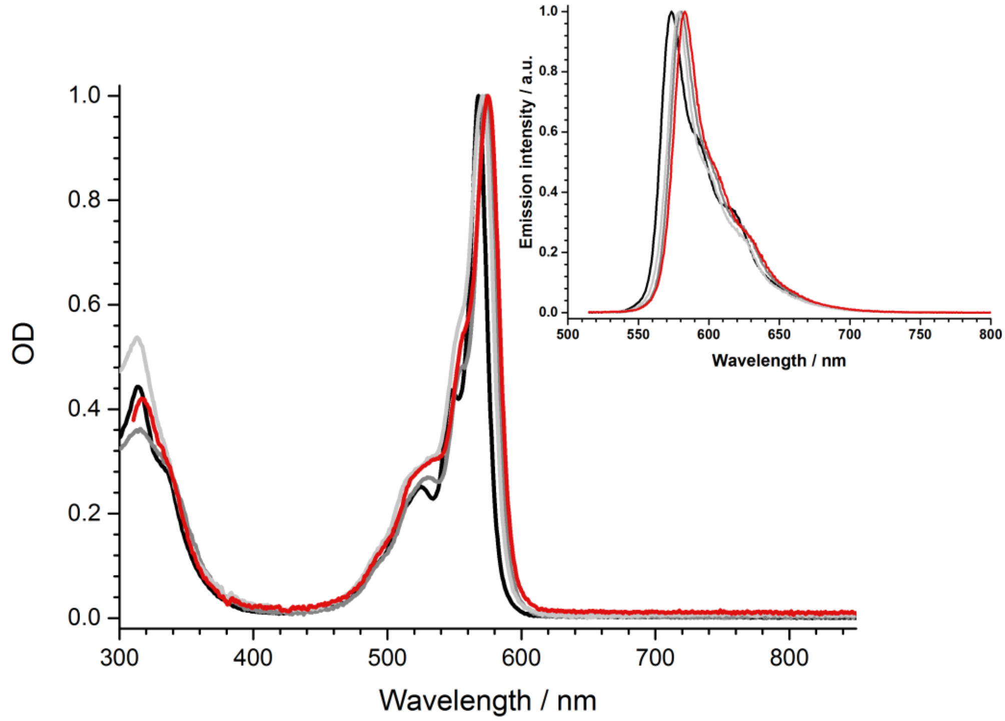

Figure S28. Absorption and fluorescence spectra (inset) upon 500 nm photoexcitation of **5** in cyclohexane (black), toluene (dark grey), THF (light grey), and benzonitrile (red).

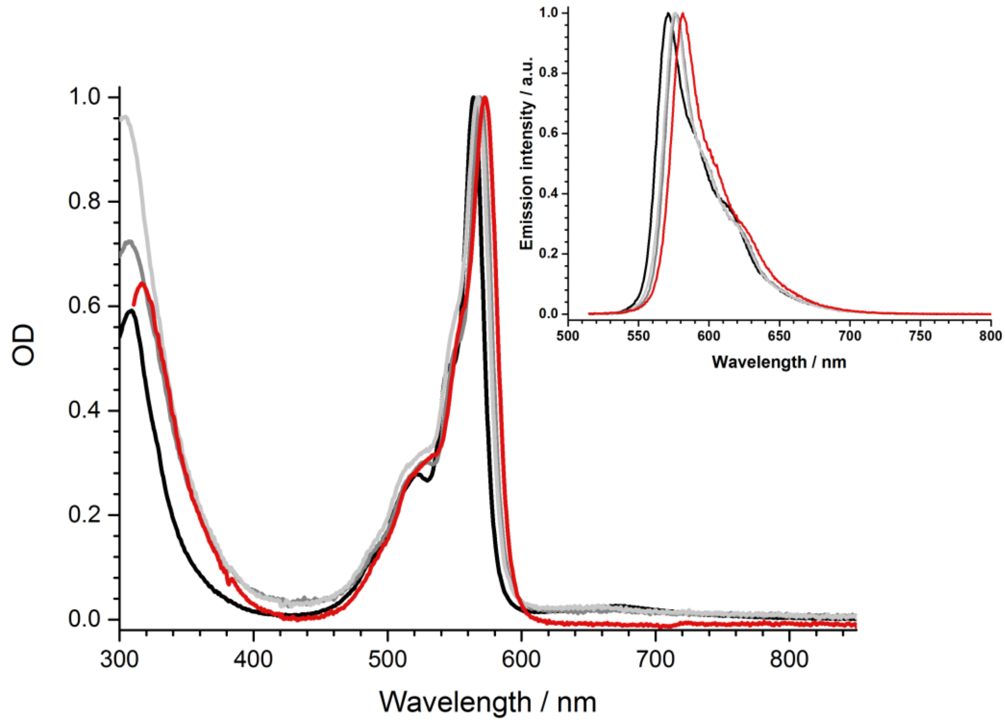

Figure S29. Absorption and fluorescence spectra (inset) upon 500 nm photoexcitation of **6** in cyclohexane (black), toluene (dark grey), THF (light grey), and benzonitrile (red).

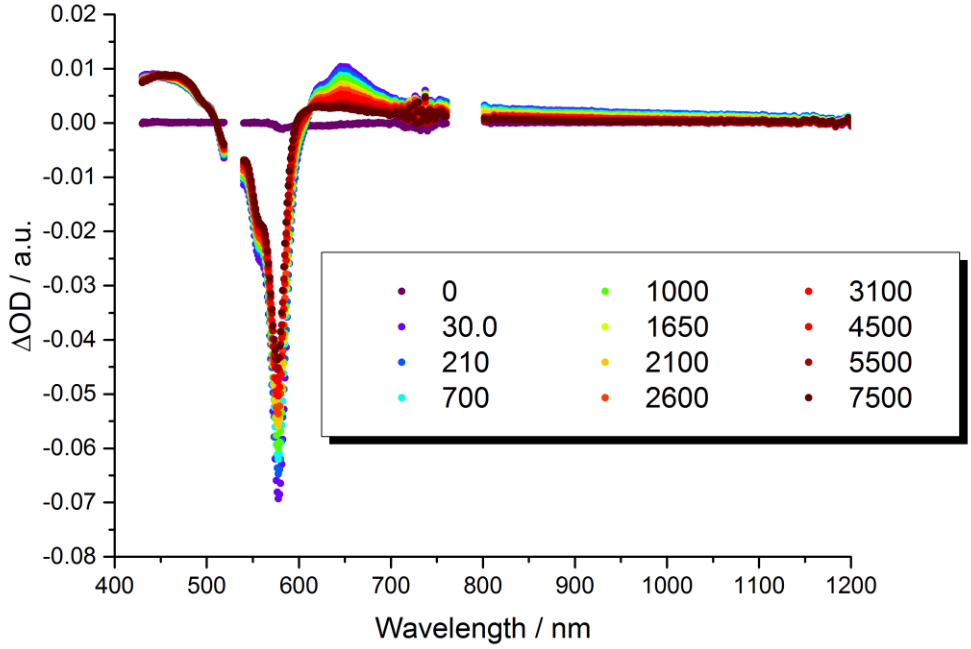

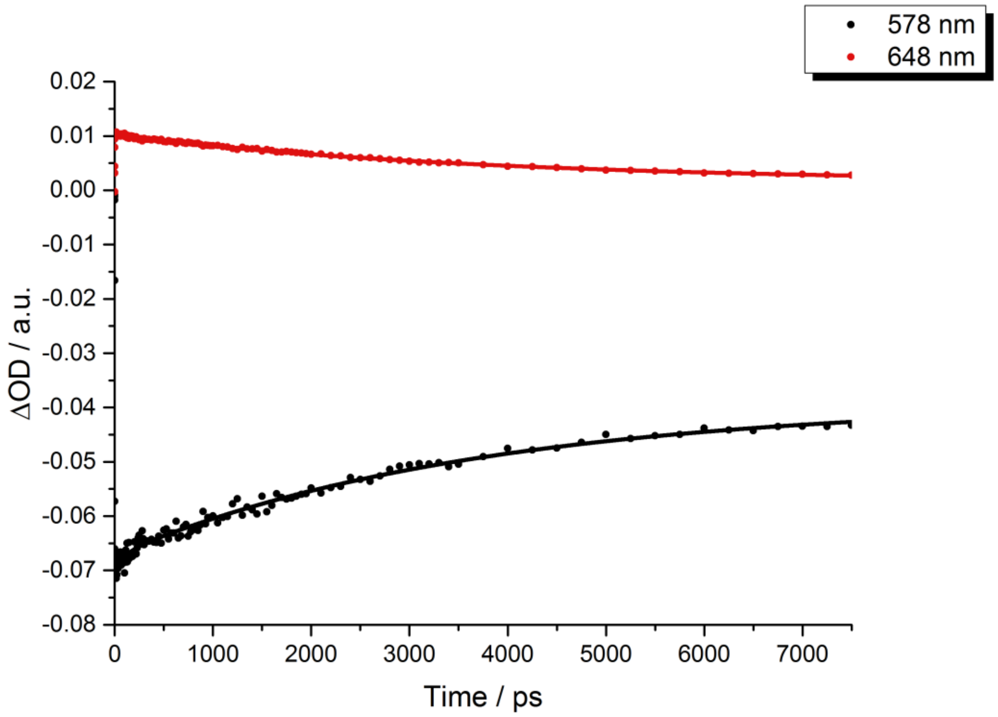

Figure S30. Upper part – differential absorption spectra (visible and near-infrared) obtained upon femtosecond pump probe experiments (530 nm) of chloro-substituted SubPc **5** in toluene with several time delays between 0 and 7500 ps at room temperature – see figure legend for details. Lower part – time absorption profiles of the spectra at 578 (black spectrum) and 648 nm (red spectrum) monitoring the excited state dynamics.

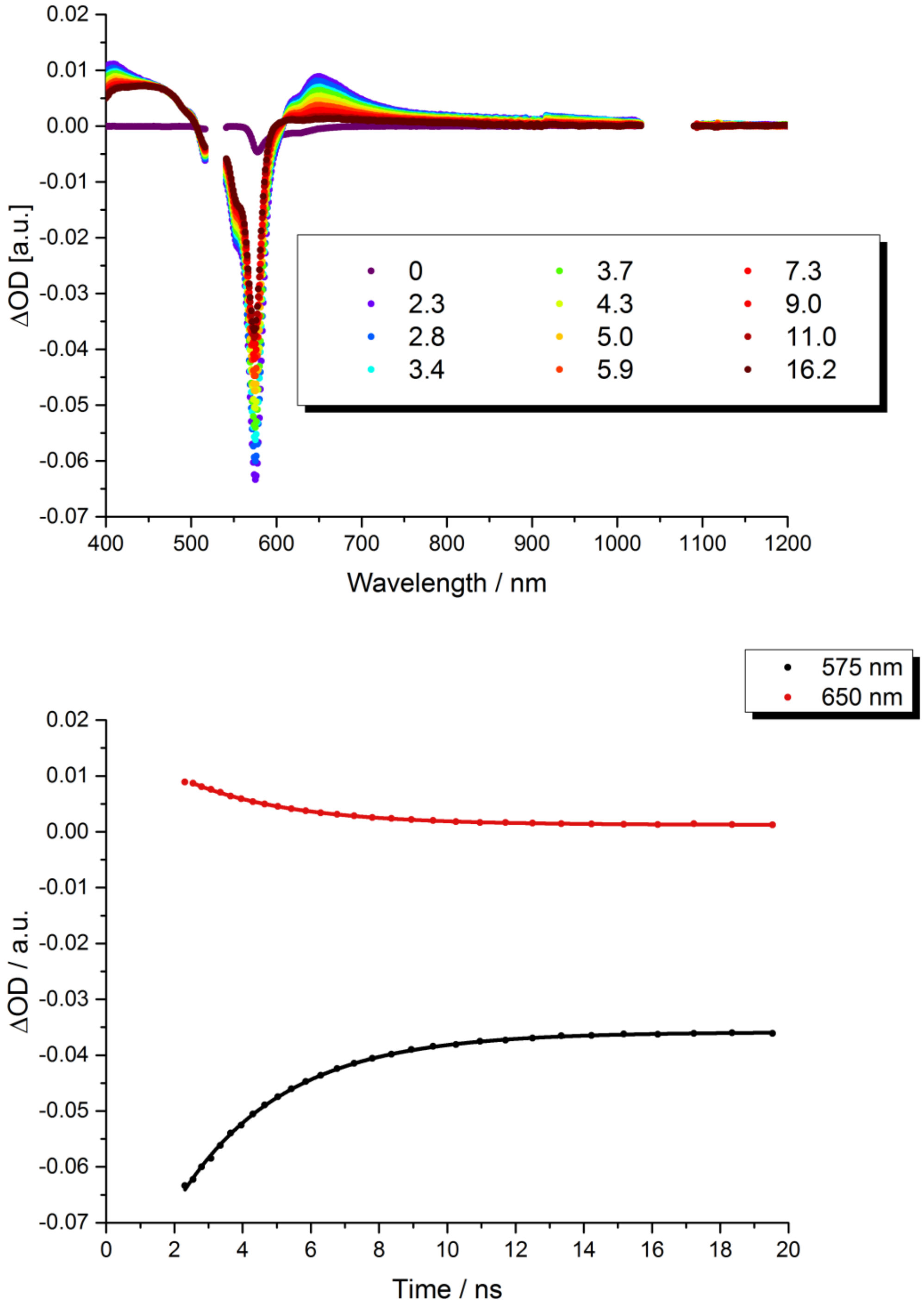

Figure S31. Upper part – differential absorption spectra (visible and near-infrared) obtained upon nanosecond pump probe experiments (530 nm) of chloro-substituted SubPc **5** in toluene with several time delays between 0 and 16.2 ns at room temperature – see figure legend for details. Lower part – time absorption profiles of the spectra at 575 (black spectrum) and 650 nm (red spectrum) monitoring the excited state dynamics.

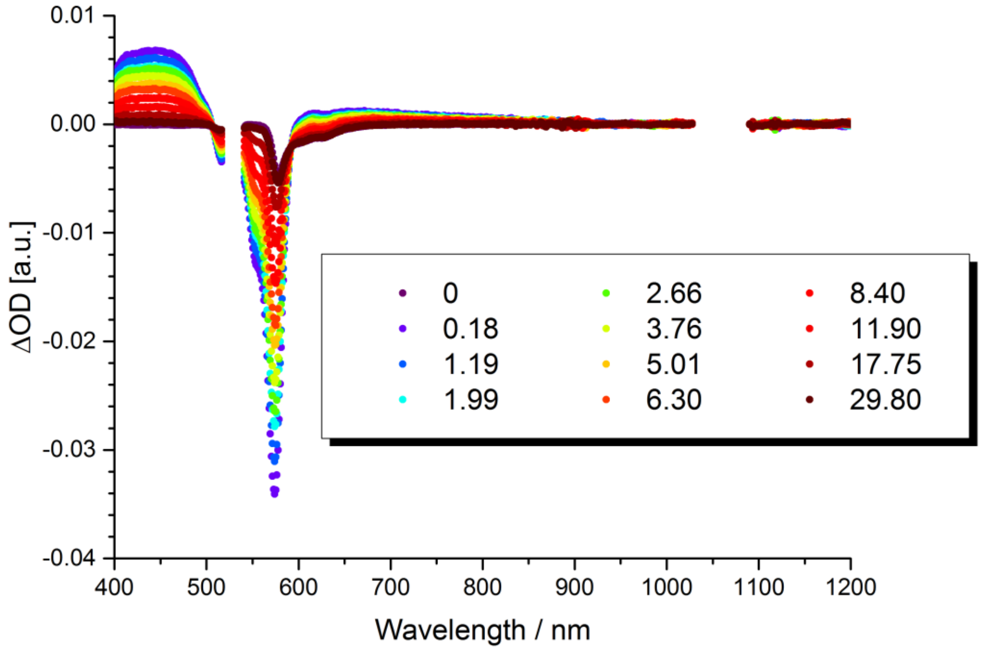

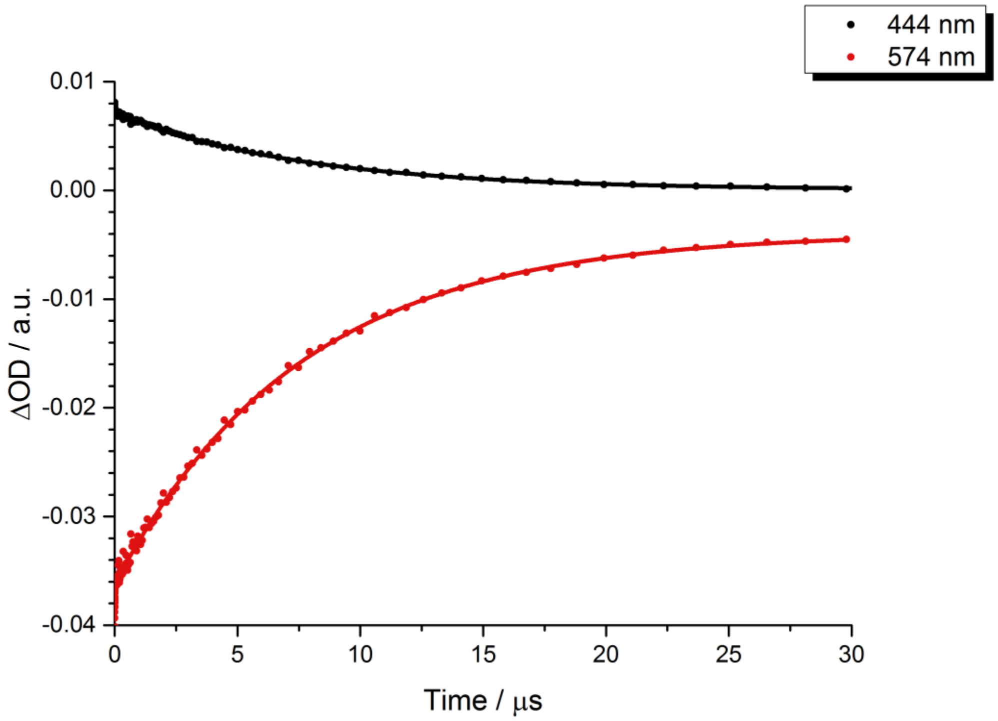

Figure S32. Upper part – differential absorption spectra (visible and near-infrared) obtained upon nanosecond pump probe experiments (530 nm) of chloro-substituted SubPc **5** in toluene with several time delays between 0 and 29.80 μs at room temperature – see figure legend for details. Lower part – time absorption profiles of the spectra at 444 (black spectrum) and 574 nm (red spectrum) monitoring the excited state dynamics.

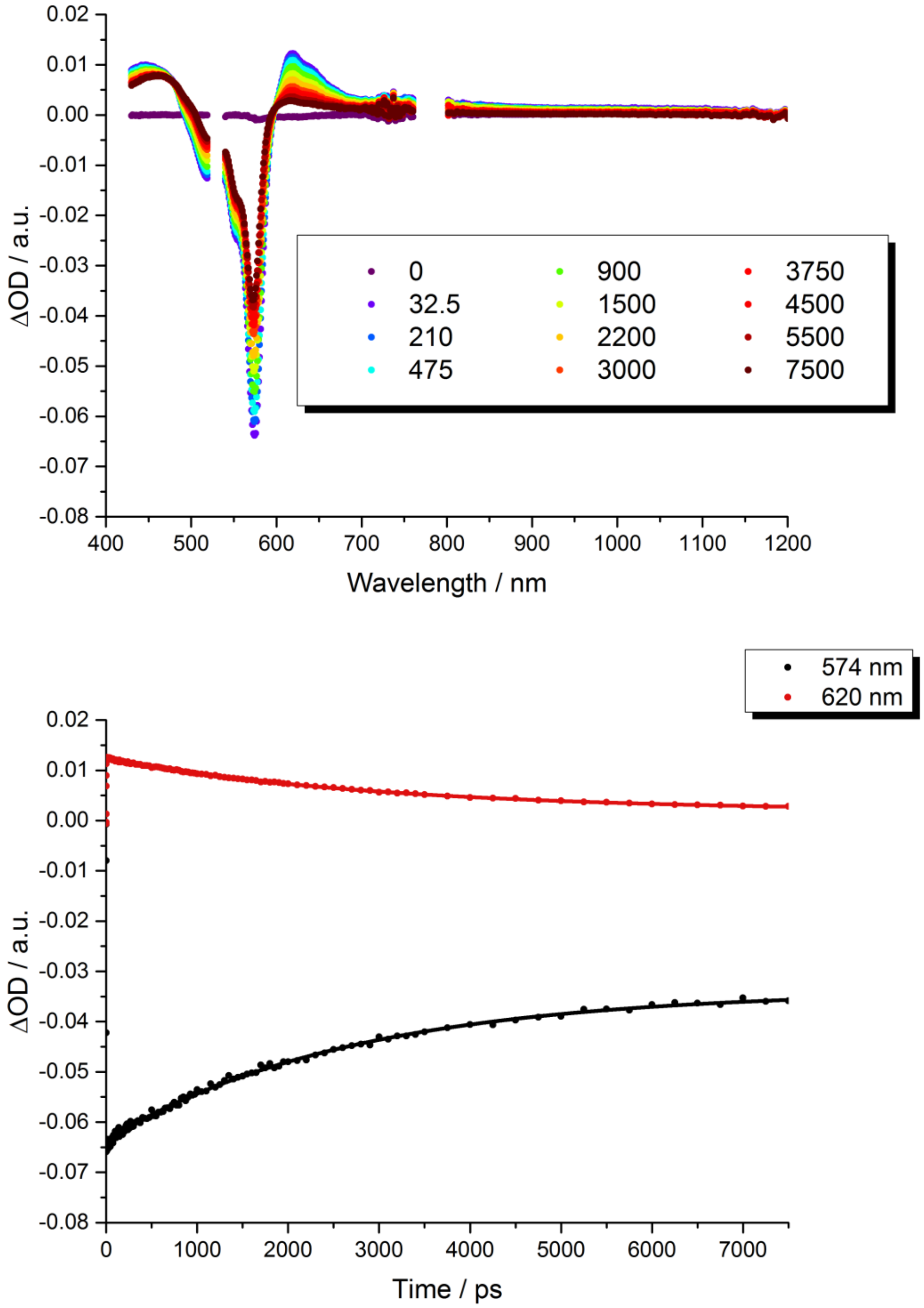

Figure S33. Upper part – differential absorption spectra (visible and near-infrared) obtained upon femtosecond pump probe experiments (530 nm) of *tert*-butyl-substituted SubPc **6** in toluene with several time delays between 0 and 7500 ps at room temperature – see figure legend for details. Lower part – time absorption profiles of the spectra at 574 (black spectrum) and 620 nm (red spectrum) monitoring the excited state dynamics.

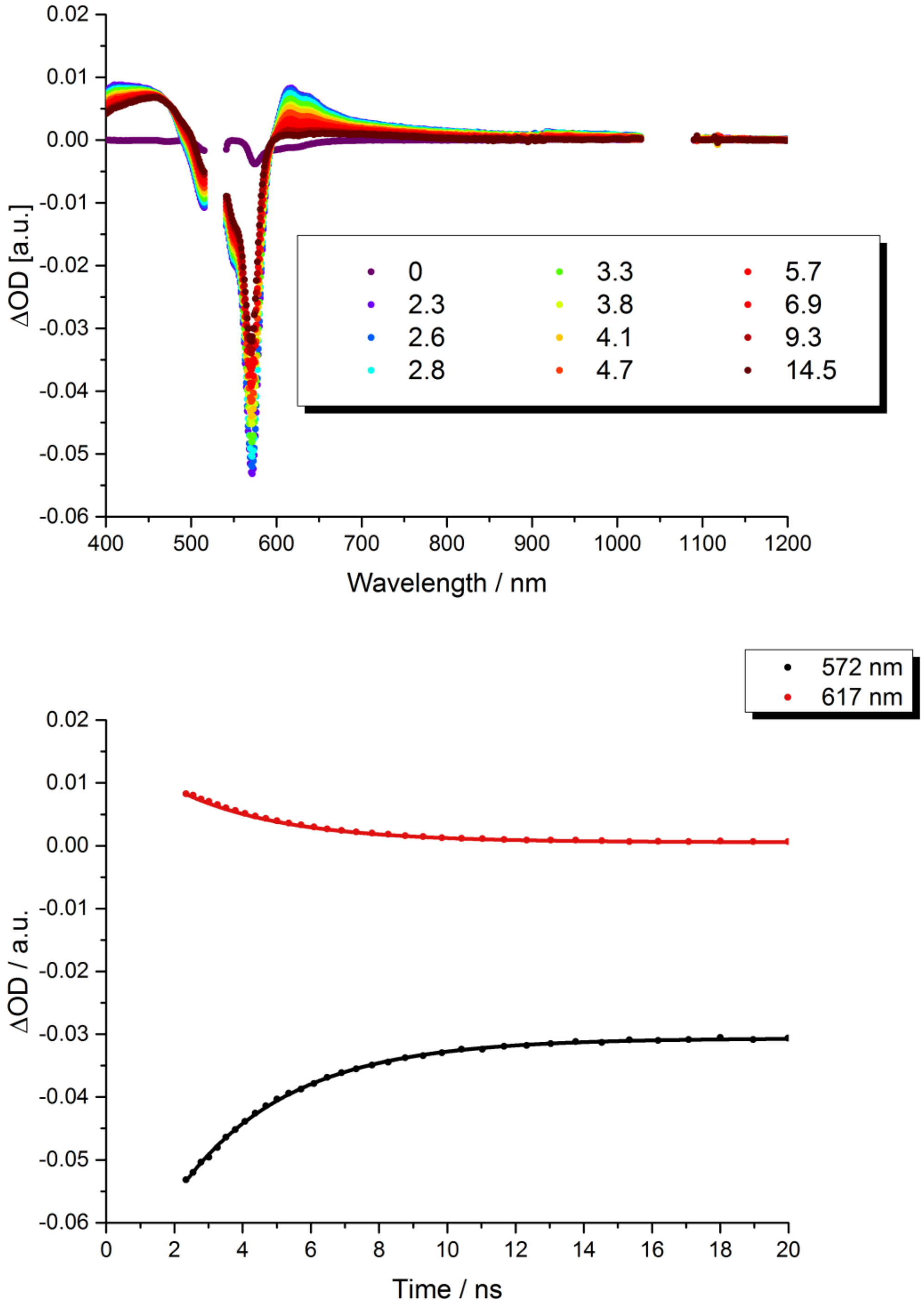

Figure S34. Upper part – differential absorption spectra (visible and near-infrared) obtained upon nanosecond pump probe experiments (530 nm) of *tert*-butyl-substituted SubPc **6** in toluene with several time delays between 0 and 14.5 ns at room temperature – see figure legend for details. Lower part – time absorption profiles of the spectra at 572 (black spectrum) and 617 nm (red spectrum) monitoring the excited state dynamics.

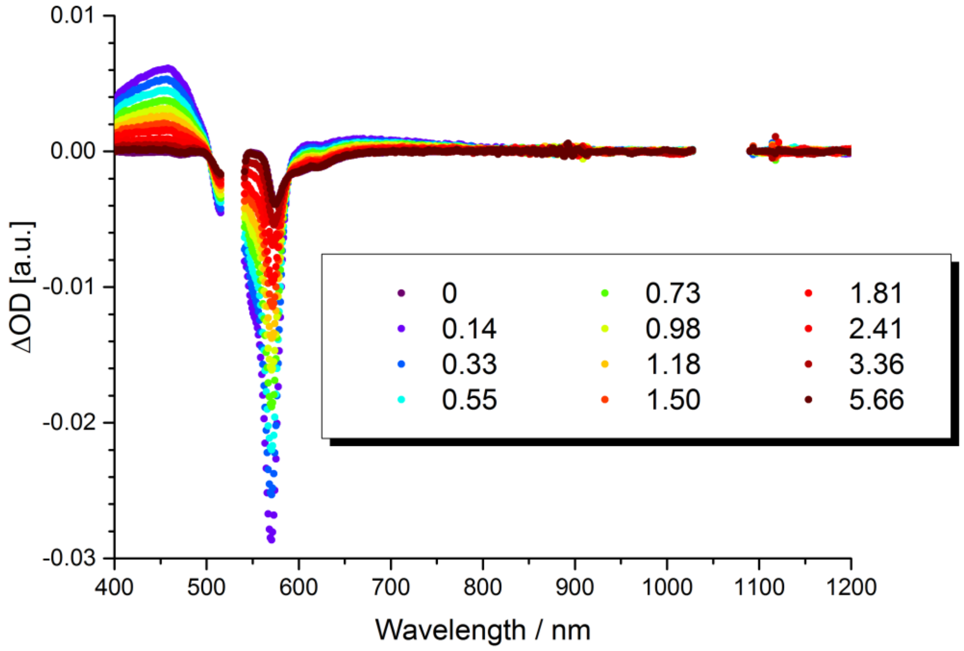

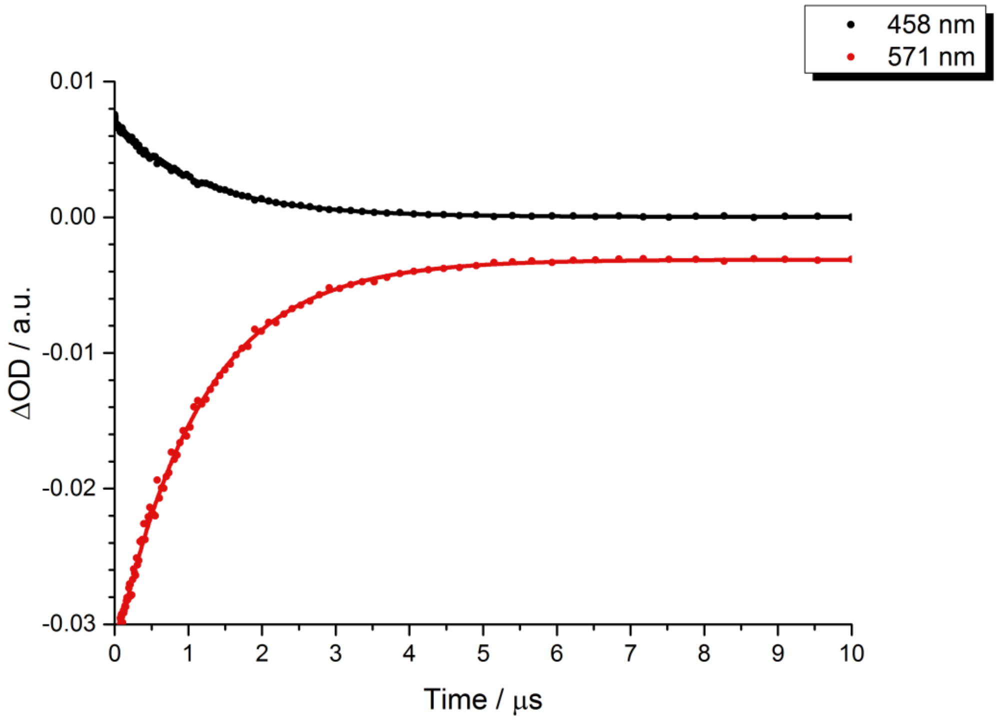

Figure S35. Upper part – differential absorption spectra (visible and near-infrared) obtained upon nanosecond pump probe experiments (530 nm) of *tert*-butyl-substituted SubPc **6** in toluene with several time delays between 0 and 5.66 µs at room temperature – see figure legend for details. Lower part – time absorption profiles of the spectra at 458 (black spectrum) and 571 nm (red spectrum) monitoring the excited state dynamics.

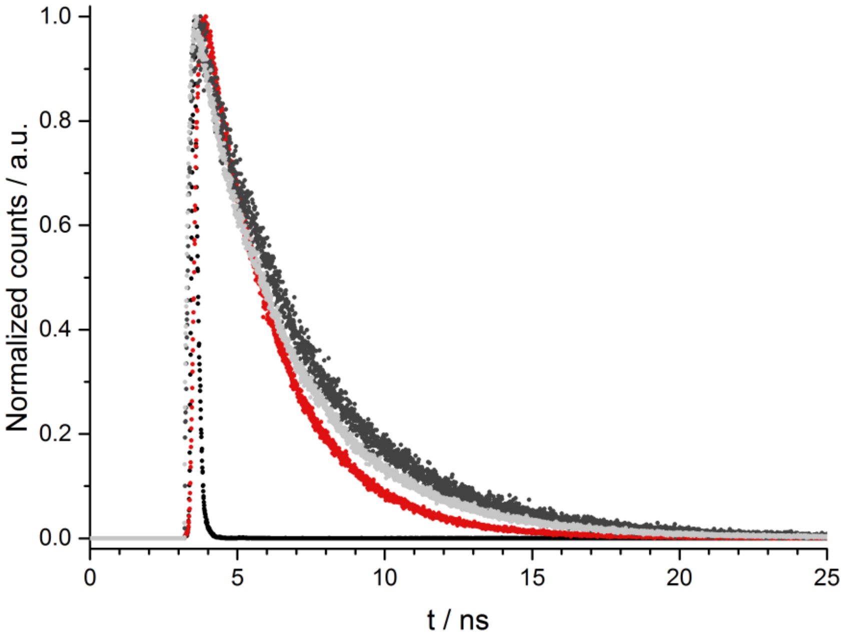

Figure S36. Fluorescence decay profiles of SubPc-SubPc' dimer **1** (red; A_{exc} = 0.15, Exc @ 647 nm, Em @ 720 nm), chloro-substituted SubPc **5** (dark grey; A_{exc} = 0.15, Exc @ 403 nm, Em @ 580 nm), and *tert*-butyl-substituted SubPc **6** (light grey; A_{exc} = 0.15, Exc @ 403 nm, Em @ 580 nm) in toluene with the instrument response function (black; prompt).

6. DFT and Semiempirical Configuration-Interaction Calculations

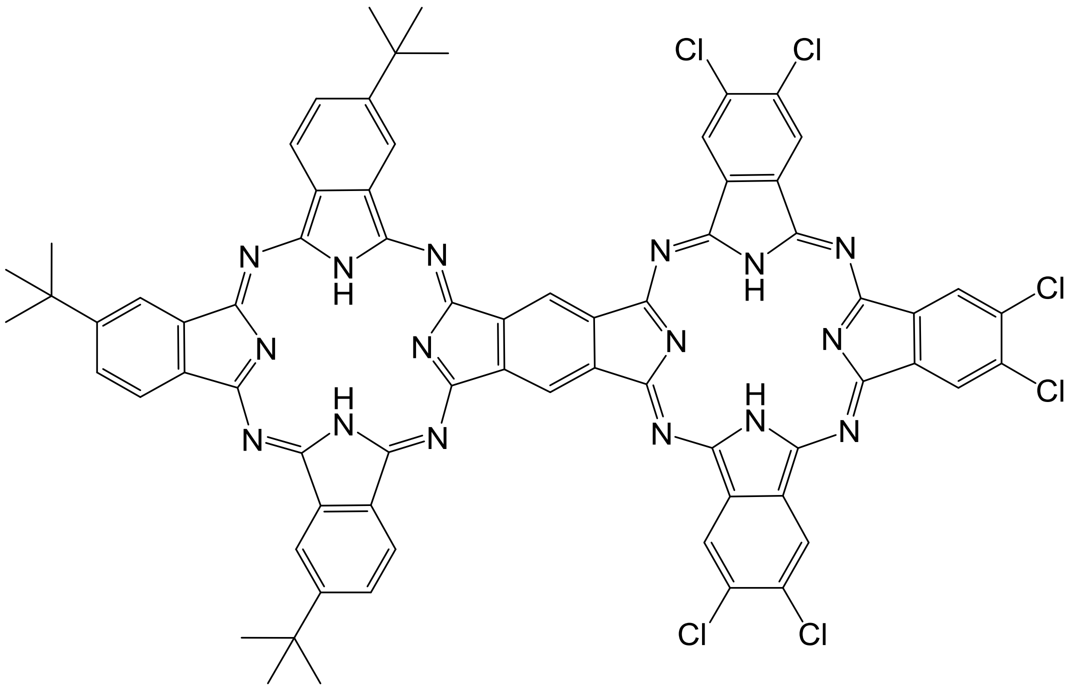

Chart S1. The structure of the reference compound **7**

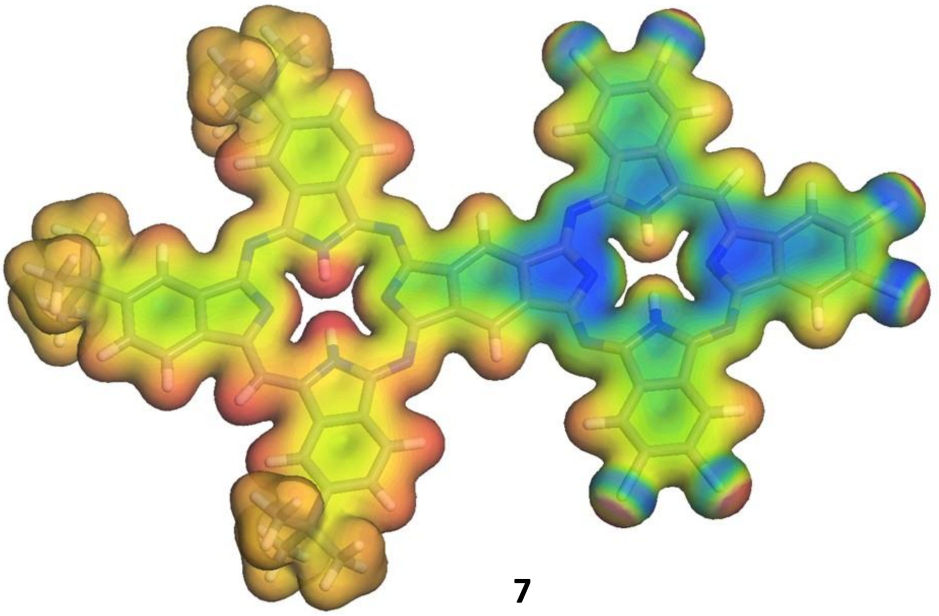

7

Figure S37. The molecular electrostatic potential calculated at the 0.001 a.u. isodensity surfaces of the S5 state of **7**. The color scale ranges from -0.3 a.u. (blue) to 0.2 a.u (red) (approximately -190 to +125 kcal mol^{-1}).

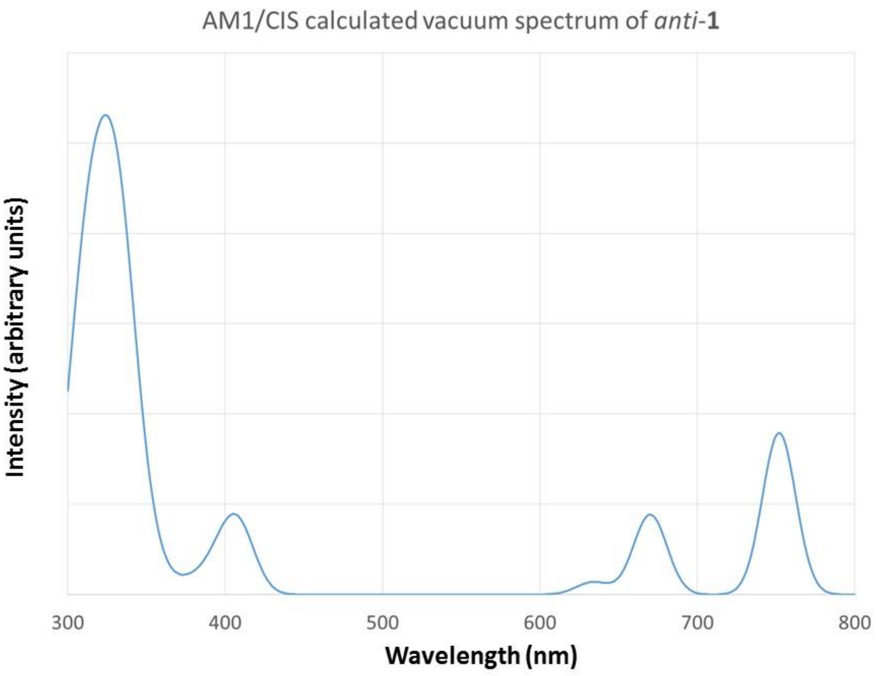

Figure S38. Simulated spectrum of anti-**1** *in vacuo* based on the AM1/CIS-calculated transition energies and oscillator strengths and an assumed Gaussian peak half-width of 25 nm.

Gaussian’09 Archive files defining the B3LYP/6-31G(d)-optimized geometries of **syn-** and **anti-1** and **7**.

<u>Syn-1</u> (Nimag = 0, ZPE = 454.06 kcal mol^{-1})


```
1\1\FAU-CCC-CCDH171\FOpt\RB3LYP\6-31G(d)\C50H30B2Cl6N12\CLARK\09-Mar-2
 015\0\\# b3lyp/6-31G(d) opt name=clark\\syn subphthalocyanine dimer\\0
 ,1\C,-0.6140150843,0.6748024124,-1.8693114931\C,0.0139377889,-0.518088
 7869,-1.5127100134\C,1.3851373913,-0.4597948002,-1.2859205761\C,2.1138
 621813,0.7872947302,-1.3571615802\C,1.4784190606,1.9881276649,-1.65588
 22666\C,0.1149341097,1.9222744695,-1.9405734414\C,3.4879352162,0.49833
 97009,-0.9697525371\N,3.555653395,-0.8645489058,-0.8470420461\C,2.3317
 631414,-1.4802703321,-0.8567435503\C,-2.0098718211,0.9835015314,-2.127
 2255034\N,-2.031444779,2.3070713583,-2.477449293\C,-0.8531403949,2.963
 0585966,-2.2403047861\N,2.1240007472,-2.6776660272,-0.2915486673\C,3.1
 237722778,-3.1942520036,0.4403921546\N,4.371564454,-2.6348612398,0.486
 2319228\B,4.8093776006,-1.5753969263,-0.4663065035\N,5.5619569139,-0.5
 976903679,0.3698712234\C,5.4317148956,0.7554518888,0.2148063854\N,4.45
 43785876,1.3104236103,-0.5193287015\B,-3.2935612125,3.0253529493,-2.81
 39081843\N,-4.3529877224,2.3192968052,-2.0426286402\C,-4.2629650884,0.
 9918231487,-1.7029632805\N,-3.1242521848,0.2979645839,-1.8183147663\N,
 -0.7904642667,4.2918650944,-2.046455465\C,-1.9481910717,4.9531838232,-
 1.9292430632\N,-3.1630509521,4.3556817878,-2.1589509154\C,-5.356659458
 9,2.9832056276,-1.3905535187\N,-5.3330724164,4.3098647225,-1.190237852
 \C,-4.2016404976,4.9598337173,-1.5034617245\C,5.0707691208,-2.93700011
 01,1.627395813\N,6.0466116657,-2.1515419102,2.1040906701\C,6.225314959
 8,-0.9611870731,1.5145393904\C,-5.4814548198,0.703259919,-0.957495426\
 C,-6.1642267598,1.9437972083,-0.7616158188\C,-3.6645270576,6.221621212
 6,-1.0059648975\C,-2.2600830019,6.2161114238,-1.2723996708\C,6.3086759
 642,1.3414223987,1.2177723188\C,6.803434529,0.2718681741,2.0272360264\
 C,4.3091233622,-3.9967748193,2.2710381283\C,3.095900235,-4.15674869,1.
 5318050528\C,-7.330479244,2.0062537928,-0.0038377558\C,-7.8643906545,0
 .8355464888,0.5548938915\C,-7.1719030737,-0.3796192391,0.351071201 8\C,
 -5.9879590316,-0.4589354918,-0.3808944281\C,-1.4706821724,7.2716371068
```

```
,-0.8224816639\C,-2.0859611926,8.3241236369,-0.1461000996\C,-3.4727388
187,8.3511157128,0.1255951395\C,-4.2551642823,7.2691409617,-0.30444989
21\C,7.6069896187,0.5362899816,3.1350497334\C,7.9374444561,1.858816355
3,3.4173672641\C,7.4466390274,2.9188536956,2.6147623951\C,6.6211161758
,2.6648564158,1.5231615776\C,2.114663248,-5.0472874166,1.963631865\C,2
.3544009782,-5.7957903621,3.1124841792\C,3.5570518635,-5.6375810161,3.
8455226519\C,4.5297869738,-4.7298841575,3.4358259872\H,-0.5396165376,-
1.446867447,-1.4234892986\H,2.0175978149,2.9293971066,-1.6734887405\H,
-7.8156593858,2.9661997919,0.1404436117\C,-9.1729219436,0.9131453942,1
.3643550482\H,-7.5649730608,-1.2936538722,0.7799609521\H,-5.4739419345
,-1.4072606063,-0.5037234965\H,-0.3996230909,7.2765973355,-0.999762985
2\H,-1.463746625,9.1475904117,0.1835476708\C,-4.1439524625,9.519312433
1,0.8727696976\H,-5.3210633449,7.2352738538,-0.1033911589\H,7.97472604
62,-0.2645600274,3.7664195789\Cl,8.9661775863,2.1805880135,4.789189307
1\Cl,7.8564849034,4.5761388708,2.9753292249\H,6.2369528345,3.484600910
8,0.9267840968\H,1.1835642799,-5.16355837,1.4207096979\Cl,1.1337100685
,-6.9290062865,3.6324030344\Cl,3.8517877506,-6.5718737586,5.2891105173
\H,5.4381177883,-4.6055727945,4.0143597225\C,-9.6067359368,-0.45625995
34,1.9211274348\C,-10.3055633548,1.4428766529,0.4519370171\C,-8.982785
5112,1.8795847963,2.5582545667\C,-4.8308386104,8.9846003361,2.15255281
24\C,-5.2071880767,10.1679658477,-0.0463435856\C,-3.1396178028,10.6109
722848,1.2887574771\H,-8.8596689648,-0.8774987858,2.6035798963\H,-9.79
21404579,-1.1838079799,1.1227687604\H,-10.5392705275,-0.3431931228,2.4
847095537\H,-10.4688935465,0.7746695232,-0.4011416942\H,-10.0800051579
,2.439106092,0.057584171\H,-11.2442043217,1.5115367409,1.0148116522\H,
-9.9093048403,1.9475201018,3.1408051017\H,-8.7228092712,2.8911453107,2
.229658529\H,-8.1870376577,1.5289686079,3.2251830486\H,-5.5983931923,8
.237814526,1.924546259\H,-5.3168473202,9.8062453012,2.692102677\H,-4.
1012798755,8.5205504379,2.8258583437\H,-5.9816085028,9.4529553273,-0.3
428129858\H,-4.7493084269,10.5630089003,-0.9602427537\H,-5.700805293,1
0.9981241726,0.4730478045\H,-2.6383483317,11.0587952597,0.4231579928\H
,-2.3718483095,10.2249761802,1.9690742509\H,-3.6687743059,11.414221082
5,1.812985953\Cl,-3.6333933733,3.1186999746,-4.6561081617\Cl,5.7805194
025,-2.2262906061,-1.9267895523\\Version=EM64L-G09RevC.01\State=1-A\HF
=-5391.658718\RMSD=6.599e-09\RMSF=2.180e-06\Dipole=-2.2402145,1.428482
8,2.0903954\Quadrupole=12.1476088,14.6389858,-26.7865945,0.8786616,-12
.486414,4.9166977\PG=C01 [X(C50H30B2Cl6N12)]\\@
```


<u>Anti-1</u> (Nimag = 0, ZPE = 453.86 kcal mol^{-1})


```
1\1\FAU-CCC-CCDH173\FOpt\RB3LYP\6-31G(d)\C50H30B2Cl6N12\CLARK\09-Mar-2
 015\0\\# b3lyp/6-31G(d) opt name=clark\\anti subphthalocyanine dimer\\
 0,1\C,-0.4704736796,0.709721244,-0.6480367032\C,0.0778241959,-0.418501
 9056,-0.0388825098\C,1.3532721642,-0.2821630694,0.4993264339\C,2.07706
 68371,0.9680700893,0.429804976\C,1.5325449717,2.0942949961,-0.17858288
 79\C,0.2534391759,1.9601720512,-0.7175539179\C,3.3916542991,0.73005912
 85,1.0102466386\N,3.3417545977,-0.5399043064,1.5219123585\C,2.24319431
 19,-1.2535640216,1.1207120911\C,-1.7837761805,0.9471267723,-1.22166926
 72\N,-1.7371427996,2.2198500658,-1.7247995919\C,-0.6349850877,2.931333
 5024,-1.3321432752\N,2.2266636097,-2.5935045843,1.0918103001\C,3.38845
 50439,-3.2282511637,1.3142992762\N,4.519098077,-2.5763848153,1.7257454
 953\B,4.4981832724,-1.1706319605,2.2188840939\N,5.7011537647,-0.534695
 3123,1.6121112823\C,5.6810833679,0.7316277285,1.0938437448\N,4.5413520
 967,1.4044792705,0.8691968785\B,-2.9059068734,2.8592769745,-2.39372090
 01\N,-4.0987101658,2.218206205,-1.7755778725\C,-4.0759601752,0.9418408
 227,-1.2690042911\N,-2.9354195539,0.2713258313,-1.0666287135\N,-0.6175
 766362,4.2748027226,-1.2894846724\C,-1.776678635,4.9132572255,-1.49005
 03822\N,-2.9167735327,4.2597130547,-1.8891858018\C,-5.2165051324,2.925
 2732445,-1.4235694859\N,-5.2388608542,4.2662858752,-1.3815168167\C,-4.
 069463515,4.9064989578,-1.5337919164\C,5.6818143971,-3.2267597266,1.39
 86822548\N,6.8504323524,-2.5861281798,1.2553820518\C,6.8284081224,-1.2
 463292107,1.2884765333\C,-5.4195358898,0.7088097569,-0.75500154\C,-6.1
 313827903,1.9446960634,-0.8498112244\C,-3.6499982491,6.2306156327,-1.0
 882359563\C,-2.2208620854,6.2336803607,-1.0623981411\C,7.0292367076,0.
 9621964905,0.5957186859\C,7.7439515045,-0.2700006941,0.7175891734\C,5.
 2664783415,-4.5491239343,0.9557033773\C,3.8377961435,-4.5501286526,0.9
 025303312\C,-7.4250739287,2.0650054537,-0.3496851789\C,-8.0552268583,0
```

```
.9551780649,0.2325346477\C,-7.3333674992,-0.2573949564,0.3157195738\C,
-6.0276840681,-0.3909136072,-0.154413274\C,-1.5439377403,7.3535714105,
-0.5853698024\C,-2.2884481302,8.4563757186,-0.1692037122\C,-3.70185085
21,8.4744762527,-0.185900557\C,-4.3756481997,7.33204945,-0.6426954647\
C,9.0471897785,-0.3827402693,0.2364630287\C,9.6438096579,0.7362045567,
-0.3379562064\C,8.9350135246,1.957524843,-0.4591415237\C,7.6234813544,
2.0699168028,-0.0062382929\C,3.1469438246,-5.662063912,0.4241565387\C,
3.8769116823,-6.7789307963,0.027137585\C,5.2931995532,-6.778223571,0.0
801994321\C,5.991249334,-5.660970105,0.5301200935\H,-0.465367327,-1.35
61834704,0.013251772\H,2.0752995667,3.0322212465,-0.2308654583\H,-7.93
1316958,3.022286905,-0.4204389903\C,-9.4949199198,1.0958811205,0.76283
96484\H,-7.8010683358,-1.1245946687,0.7665667082\H,-5.4966284728,-1.33
28717884,-0.057928032\H,-0.4592528824,7.3678565639,-0.5421142781\H,-1.
7498274784,9.3273300468,0.1848812632\C,-4.5165409083,9.6946615048,0.28
43725044\H,-5.4599687431,7.2908218517,-0.6577800177\H,9.5934670201,-1.
316418183,0.3071471553\Cl,11.2853001306,0.5959330858,-0.9121397508\Cl,
9.6829682702,3.3556509236,-1.1867467519\H,7.0844818284,3.0035760207,-0
.12038352\H,2.0646199297,-5.6668667633,0.3623109999\Cl,3.0054352804,-8
.1779390648,-0.5446411488\Cl,6.2061262973,-8.1768720315,-0.4239744897\
H,7.0751269668,-5.6660645547,0.549009215\C,-10.0279972143,-0.211185101
9,1.379830501\C,-10.4320641656,1.4972765191,-0.4014953239\C,-9.5331384
962,2.1926630471,1.8543198737\C,-5.4319288048,9.2761795237,1.460237813
5\C,-5.3876672604,10.2102430793,-0.8862447945\C,-3.621918957,10.853450
0122,0.7640838102\H,-9.4250583644,-0.5358205272,2.2355440644\H,-10.057
2338481,-1.0279890507,0.6497045985\H,-11.0510456247,-0.0552755365,1.73
9291039\H,-10.4275204275,0.7363550408,-1.1901085429\H,-10.1370935905,2
.4494961452,-0.8544415087\H,-11.461442979,1.6052429558,-0.0391269281\H
,-10.5532553099,2.3076722582,2.2400269476\H,-9.2125286811,3.1660049656
,1.4688114193\H,-8.8796398384,1.9335111006,2.6950113827\H,-6.132642045
2,8.4854521392,1.172913091\H,-6.0219710219,10.1339939125,1.8046743631\
H,-4.8410207563,8.9087894798,2.306901085\H,-6.0861346136,9.4465468063,
-1.2437323049\H,-4.7650467335,10.5170404164,-1.7342762461\H,-5.9774023
388,11.0775908608,-0.566070707\H,-2.9649460533,11.2218352115,-0.032020
3825\H,-2.9989218558,10.5634538013,1.617869307\H,-4.2500495729,11.6914
482302,1.0855782579\Cl,-2.8614991481,2.7293868585,-4.264827649\Cl,4.42
56994847,-1.0248577574,4.0837920082\\Version=EM64L-G09RevC.01\State=1-
A\HF=-5391.6587195\RMSD=7.359e-09\RMSF=8.224e-06\Dipole=-2.3615179,1.4
1671,0.8894893\Quadrupole=20.2944546,13.2120659,-33.5065204,-8.09049,-
18.2227709,8.2365454\PG=C01 [X(C50H30B2Cl6N12)]\\@
```


<u>7</u> (Nimag = 0, ZPE = 675.75 kcal mol^{-1})


```
1\1\FAU-CCC-CCDH171\FOpt\RB3LYP\6-31G(d)\C72H50Cl6N14\CLARK\12-Mar-201
5\0\\# b3lyp/6-31G(d) opt name=clark freq\\phthalocyanine dimer\\0,1\C
,-0.5592658049,0.8862080455,0.4526097377\C,0.1644554109,-0.2994053253,
0.5782784671\C,1.5461034345,-0.150678563,0.6666423633\C,2.1746827039,1
.1154514448,0.6315320594\C,1.4535204159,2.2995155316,0.506133569\C,0.0
690358952,2.152086351,0.4174706114\C,3.6098686224,0.8408034696,0.75029
36782\N,3.8483726812,-0.5050158711,0.8517377202\C,2.6351038465,-1.1211
028208,0.8046629733\C,-1.9933507679,1.158400525,0.3340761433\N,-2.2289
897803,2.503036412,0.2329002817\C,-1.0178559167,3.1233116281,0.2795209
876\N,2.3813832823,-2.4344408065,0.8693462682\C,3.314001627,-3.3525060
204,0.9931109324\N,4.6783861888,-3.1938917221,1.0797405416\N,6.5158861
892,0.4754336854,0.9789845933\C,5.8089170276,1.6455433734,0.8553410437
\N,4.5034046608,1.8256275619,0.7516404204\N,-4.8937102517,1.5166714986
,0.1061643684\C,-4.1886277597,0.3415135101,0.2300903794\N,-2.883397299
3,0.1683968342,0.3333155355\N,-0.7598213637,4.4357613237,0.215292633\
C,-1.688034647,5.3586651472,0.0915739244\N,-3.0549222485,5.1952035638,
0.0051519078\C,-6.2502940166,1.3135058546,0.022959949\N,-5.0126761982,
6.60841693,-0.2223437462\C,-3.7219408446,6.3984041619,-0.1177345467\C,
5.3469915458,-4.3993177852,1.2028167824\N,6.6343689786,-4.6129211101,1
.3073321033\C,7.8755832974,0.6785478628,1.0624390578\C,-5.1751984465,-
0.7179643736,0.2271204923\C,-6.4516732024,-0.1218771527,0.0992349521\C
,-2.6956626374,7.4318014666,-0.1099196691\C,-1.4465793925,6.7902706328
,0.0188117079\C,6.7940728902,2.7061085033,0.8581797251\C,8.0708368353,
2.1115036493,0.9859608048\C,4.318097399,-5.4274011763,1.194669275\C,3
.0707702741,-4.7848419813,1.0659481174\C,-7.6044551608,-0.9121661253,0
```

.0674541688\C,-7.4989906602,-2.3042842583,0.1627168441\C,-6.2079080522
,-2.8745231478,0.290142448\C,-5.0509983171,-2.1041466683,0.3234169154\
C,-0.2743751706,7.5425466683,0.0546494671\C,-0.379677701,8.9285793157,
-0.0398560921\C,-1.6227454007,9.5930806145,-0.169711464\C,-2.790748712
5,8.8179759774,-0.2037556601\C,9.2182854152,2.9107858151,1.0167556399\
C,9.0701950656,4.2890002381,0.9194665536\C,7.7852106945,4.8796015786,0
.7913656363\C,6.6433564762,4.0898653794,0.7602674906\C,1.8845236889,-5
.5144779205,1.0278157542\C,1.9658876988,-6.9007140579,1.1207876223\C,3
.2210401334,-7.5482977372,1.2503782957\C,4.403831036,-6.8149268355,1.2
880041587\H,-0.318055368,-1.2704633924,0.605174734\H,1.933305755,3.271
9226388,0.4789428255\H,-8.5797222879,-0.4443740146,-0.0310616096\C,-8.
7773694026,-3.1653337967,0.1266951384\H,-6.1069826142,-3.9506648081,0.
3651821885\H,-4.0721398655,-2.5628954304,0.4216043726\H,0.6918208589,7
.057814966,0.1534276088\H,0.5339567763,9.5108172388,-0.0116277913\C,-1
.7281321144,11.1267150927,-0.273543792\H,-3.7707043519,9.2738587838,-0
.3018705784\H,10.2125215231,2.4884708081,1.1135510449\Cl,10.5072665085
,5.2802591447,0.9588604995\Cl,7.6074275978,6.6118725353,0.6698894084\H
,5.6610903923,4.5378172084,0.6624666121\H,0.9246290956,-5.0209847786,0
.928781757\Cl,0.4851637322,-7.8245294762,1.0742544158\Cl,3.3198791274,
-9.2867877768,1.3669132849\H,5.3605758412,-7.3145549594,1.3871280754\C
,-8.4778268717,-4.672572147,0.2431071954\C,-9.5205648955,-2.9263560426
,-1.2094899494\C,-9.6984171753,-2.7674989833,1.3049312129\C,-2.5833945
175,11.6613437414,0.900412795\C,-2.4053000156,11.5034046716,-1.6133294
89\C,-0.3534427249,11.8200025619,-0.2197108348\H,-7.8524066686,-5.0313
181879,-0.5821668171\H,-9.4170886908,-5.2358184844,0.212221442\H,-7.97
75530593,-4.9196017276,1.1863075463\H,-10.4358628287,-3.5292041328,-1.
248056811\H,-8.8932612092,-3.2061806812,-2.0633882877\H,-9.807747008,-
1.877173257,-1.3358661984\H,-10.6154166557,-3.3688062578,1.2899343585\
H,-9.9911211535,-1.7133166322,1.2574367978\H,-9.1995600054,-2.93268473
3,2.2666014432\H,-2.1253516837,11.4139002857,1.8647508825\H,-3.5944695
736,11.2414170649,0.8928712784\H,-2.6749742359,12.7524352976,0.8372559
514\H,-3.4110976474,11.0788266402,-1.6955958025\H,-1.8185958599,11.141
7908712,-2.4653815968\H,-2.4951641288,12.5930179368,-1.7000717063\H,0.
2952148879,11.5084448884,-1.0463490444\H,0.1698899379,11.6196275014,0.
7221623145\H,-0.4868657826,12.9046186319,-0.2973515059\C,-5.9355764039
,5.6344207498,-0.2265219489\N,-5.7367301025,4.2935813206,-0.128189467\
C,-6.9696284306,3.7000101306,-0.1782107621\C,-7.1973726573,2.322336400
5,-0.1076560306\C,-7.3610631657,5.9453247558,-0.3469960459\C,-8.024240
2837,4.7096270291,-0.3163766088\C,-8.0502189205,7.1502971516,-0.471450
2386\C,-9.4475197406,7.1322393853,-0.5691630966\C,-10.1026384216,5.877
3735901,-0.536822232\C,-9.41651557,4.6715438778,-0.4125944311\H,-4.462
2434714,2.4352130854,0.0787491403\H,-3.5196307282,4.293811718,0.028874
5644\H,-8.2318200978,1.9959273584,-0.1602210952\H,-7.4890181116,8.0768
972587,-0.4898375119\C,-10.2843922958,8.4176248428,-0.7090329947\H,-11
.1858789268,5.8455419993,-0.6114858894\H,-9.9669132705,3.7340686925,-0
.392722528\C,-11.2652645891,8.5278576209,0.4829281251\C,-11.0875178412
,8.3699454054,-2.0310447407\C,-9.4081103708,9.6840377134,-0.7266246995
\H,-11.9496741307,7.6743985961,0.5303035628\H,-11.8725187609,9.4367880
098,0.3927833077\H,-10.7226191243,8.5745170677,1.4339423523\H,-11.7659
109066,7.5111470958,-2.0672847052\H,-10.4165501093,8.3027464411,-2.894
9623339\H,-11.6934989245,9.2775841679,-2.140701989\H,-8.7067234796,9.6
823800518,-1.5685609505\H,-8.8318051533,9.7934963326,0.198952064\H,-10
.0434646918,10.5711416805,-0.8273523199\C,7.5599760423,-3.6408595707,1
.3117716569\N,7.366961879,-2.3001948108,1.2139001634\C,8.5977041214,-1
.705155982,1.2635642364\C,8.8242585442,-0.3263994918,1.1929208382\C,9.
6501118829,-2.7152558509,1.4014623083\C,8.9818883896,-3.9520501698,1.4
319400382\C,11.0395400739,-2.6606769272,1.4962889039\C,11.7432660325,-
3.8587596252,1.6213612888\C,11.0654596676,-5.09904592,1.6514577849\C,9
.675478225,-5.1511665973,1.5563637458\H,5.1416720416,-2.2917975647,1.0
558570235\H,6.0837400251,-0.4426773712,1.0062156751\H,9.8579453426,0.0
024301944,1.2453590426\H,11.5892690314,-1.7254522047,1.4764121719\Cl,1
3.4859526093,-3.7848112559,1.7402306109\Cl,11.9470042651,-6.5997851884
,1.8081645055\H,9.1573802066,-6.1031690981,1.5795276328\\Version=EM64L
-G09RevC.01\State=1-A\HF=-6301.6545487\RMSD=3.478e-09\RMSF=3.417e-06\D
ipole=-2.8405482,1.4795138,-0.2938466\Quadrupole=19.0836014,-2.5083657
,-16.5752358,14.0231243,1.6468307,0.1014841\PG=C01 [X(C72H50Cl6N14)]\\
@

7. References

1. D. S. Terekhov, K. J. M. Nolan, C. R. McArthur, C. C. Leznoff, *J. Org. Chem.* **1996**, *61*, 3034.

2. D. Wöhrle, M. Eskes, K. Shigehara, A. Yamada, *Synthesis* **1993**, 194.
